# Efficacy of neurostimulation across mental disorders: systematic review and meta-analysis of 208 randomized controlled trials

**DOI:** 10.1038/s41380-022-01524-8

**Published:** 2022-04-01

**Authors:** Joshua Hyde, Hannah Carr, Nicholas Kelley, Rose Seneviratne, Claire Reed, Valeria Parlatini, Matthew Garner, Marco Solmi, Stella Rosson, Samuele Cortese, Valerie Brandt

**Affiliations:** 1 Centre for Innovation in Mental Health, School of Psychology, University of Southampton, Southampton, UK; 2 Centre for Research on Self and Identity, School of Psychology, University of Southampton, Southampton, UK; 3 Department of Child and Adolescent Psychiatry, Institute of Psychiatry, Psychology and Neuroscience, King’s College London, London, UK; 4 Clinical and Experimental Sciences (CNS and Psychiatry), Faculty of Medicine, University of Southampton, Southampton, UK; 5 Department of Psychiatry, University of Ottawa, Ottawa, ON Canada; 6 Department of Mental Health, The Ottawa Hospital, Ottawa, ON Canada; 7 Department of Mental Health, Azienda AULSS 3 Serenissima, Venice, Italy; 8 Solent NHS Trust, Southampton, UK; 9 Division of Psychiatry and Applied Psychology, School of Medicine, University of Nottingham, Nottingham, UK; 10 Hassenfeld Children’s Hospital at NYU Langone, New York University Child Study Center, New York, NY USA

**Keywords:** Addiction, ADHD, Depression, Schizophrenia, Psychiatric disorders

## Abstract

Non-invasive brain stimulation (NIBS), including transcranial magnetic stimulation (TMS), and transcranial direct current stimulation (tDCS), is a potentially effective treatment strategy for a number of mental conditions. However, no quantitative evidence synthesis of randomized controlled trials (RCTs) of TMS or tDCS using the same criteria including several mental conditions is available. Based on 208 RCTs identified in a systematic review, we conducted a series of random effects meta-analyses to assess the efficacy of NIBS, compared to sham, for core symptoms and cognitive functioning within a broad range of mental conditions. Outcomes included changes in core symptom severity and cognitive functioning from pre- to post-treatment. We found significant positive effects for several outcomes without significant heterogeneity including TMS for symptoms of generalized anxiety disorder (SMD = −1.8 (95% CI: −2.6 to −1), and tDCS for symptoms of substance use disorder (−0.73, −1.00 to −0.46). There was also significant effects for TMS in obsessive-compulsive disorder (−0.66, −0.91 to −0.41) and unipolar depression symptoms (−0.60, −0.78 to −0.42) but with significant heterogeneity. However, subgroup analyses based on stimulation site and number of treatment sessions revealed evidence of positive effects, without significant heterogeneity, for specific TMS stimulation protocols. For neurocognitive outcomes, there was only significant evidence, without significant heterogeneity, for tDCS for improving attention (−0.3, −0.55 to −0.05) and working memory (−0.38, −0.74 to −0.03) in individuals with schizophrenia. We concluded that TMS and tDCS can benefit individuals with a variety of mental conditions, significantly improving clinical dimensions, including cognitive deficits in schizophrenia which are poorly responsive to pharmacotherapy.

## Introduction

Mental ill-health affects more than 1 billion people globally and causes ~19% of years lived with disability [1], with numbers rising following the outbreak of Covid-19 [2–4]. Non-invasive brain stimulation (NIBS) has been proposed as an intervention strategy for mental disorders. NIBS has immediate effects on neural excitability but also after effects [5], which makes it a potentially suitable therapeutic tool for mental disorders. NIBS encompasses transcranial magnetic stimulation (TMS) and transcranial direct current stimulation (tDCS). During TMS, a brief electrical current flows through a wire coil, creating a magnet field that passes through the skull and induces a current on the surface of the cortex, depolarizing neurons or their axons [6]. This leads to alterations in the activation patterns of neural populations and can be most effectively achieved using repetitive TMS (rTMS) or theta-burst stimulation (TBS). tDCS is another non-invasive neurostimulation method that uses direct electrical currents to stimulate a targeted cortical area. The neurobiological basis for the longer lasting effects of tDCS is similar to the one found in TMS [7–9], and likely involves inducing long-term potentiation (LTP)-like plasticity [10, 11].

As many mental disorders are associated with imbalances in excitability [12, 13], NIBS is a potentially effective treatment strategy for a number of mental conditons. While meta-analyses of randomized controlled trials (RCTs) of TMS or tDCS for individual mental disorders are available, to date no meta-analytic synthesis using the same criteria across a large number of mental disorders has been published. Therefore, we conducted a series of meta-analyses of RCTs of TMS and/or tDCS using the same criteria across a broad range of mental conditions. Additionally, one key issue in assessing the effectiveness of NIBS is that their size and durability depends on the stimulation site, frequency, intensity, the number of stimulation sessions, and the shape of the magnetic pulse [14]. Therefore, we conducted additional analyses according to stimulation site, frequency, and number of stimulation sessions.

## Methods

The study was registered with PROSPERO (number CRD42021250057), and followed PRISMA guidelines [15].

### Search strategy and selection process

PubMed, OVID, and Web of Knowledge databases were systematically searched, from inception until April 26th 2021, with no language/document type restrictions. We used the Pubmed search syntax “(random*) AND (“TMS” OR rTMS OR tDCS OR TMS)” combined with a list of ICD-11 mental health conditions, adapted for each database (see Supplementary Materials for full list of search terms). References of each relevant retrieved meta-analysis were hand-searched for additional eligible studies. All reports were screened for eligibility by two independent screeners. Conflicts were resolved by discussion with a senior author.

### Inclusion criteria

Studies were included if they met the following criteria: (1) randomized, sham-controlled trials using TMS and/or tDCS, (2) including children and/or adults with a primary diagnosis of a mental health condition using standardized diagnostic criteria (DSM-III/IV/5, ICD-9/10/11 or based on other standardized diagnostic tools), and (3) using standardized scales assessing core symptom severity and/or tasks measuring cognitive functioning (executive function, attention/vigilance, processing speed, and working memory).

Studies were excluded if: (1) data for core symptom severity or cognitive functioning were unavailable, (2) patients were in remission, (3) there was another concomitant intervention (e.g., pharmacotherapy/cognitive training), and (4) a crossover design was used and data for the first phase was not available.

### Outcomes

Change in core symptom severity in each mental disorder was the primary outcome. Secondary outcomes were score changes in standard cognitive functioning tasks. Change was defined as the difference in scores between baseline and after the last treatment session. Four neurocognitive domains (attention/vigilance, executive functioning, processing speed, and working memory) were chosen, and associated tasks/constructs were defined based on guidelines from the MATRICS cognitive test battery for schizophrenia [16], as in previous analyses of neurostimulation for cognitive enhancement [17, 18]. Data for follow-up assessments were beyond the scope of the present paper.

### Data extraction

Data were extracted by JH and independently checked by HC, RS, and VP Where outcome data were not available, corresponding authors were systematically contacted. Wherever available, data were extracted as baseline and endpoint means and standard deviations, or mean change scores and standard deviations. Where continuous outcome data were not available, response rates (≥50% score improvement) were extracted and pooled separately. Other extracted information included: participant demographics/baseline characteristics and intervention parameters, i.e., stimulation site, intensity/frequency, and number of sessions.

### Risk of bias assessment

Risk of bias was assessed independently by two investigators with the Cochrane risk of bias for randomized trials version 2 (RoB2) assessment tool [19]. Items include whether the allocation sequence was random, whether participants or experimenters were aware of their assigned intervention, whether an appropriate analysis was planned and used, and whether the results may have been biased by missing data.

### Statistical analysis

All analyses were conducted using Comprehensive Meta-Analysis software, version 3 [20], when two or more eligible TMS/tDCS RCTs on the same outcome were available. Data were grouped by disorder, stimulation technique (TMS/tDCS), and outcome (symptoms/cognitive domain); and pooled using random effects models based on standardized mean difference (SMD). SMD values of 0.2–0.5 were considered small, values of 0.5–0.8 medium, and values >0.8 were considered large, according to the commonly reported thresholds by Cohen (even though Cohen himself urged caution in this interpretation) [21]. Heterogeneity was assessed with Cochran’s Q test and the *I*^2^ statistic, which estimate the presence of significant heterogeneity, and the proportion of total variability due to between-study heterogeneity, respectively. Publication bias was assessed visually via funnel plots and quantitatively with the Egger’s test where at least ten studies were available. To examine sources of heterogeneity in core symptom severity outcomes among TMS trials, subgroup analyses were conducted based on: (a) stimulation technique, (b) stimulation site, (c) stimulation frequency, and (d) number of sessions. For subgroup analyses, rTMS at frequencies ≤1 Hz was defined as low frequency (LF), and rTMS at frequencies ≥5 Hz was defined as high frequency (HF). Where applicable, cTBS was grouped with LF rTMS and iTBS was grouped with HF rTMS due to the small number of available TBS trials. Sensitivity analyses were conducted excluding studies with a risk of bias assessment rated as “high”.

Post hoc analyses and changes to the pre-registred protocol are reported in the Supplementary Materials.

## Results

**Fig. 1:**
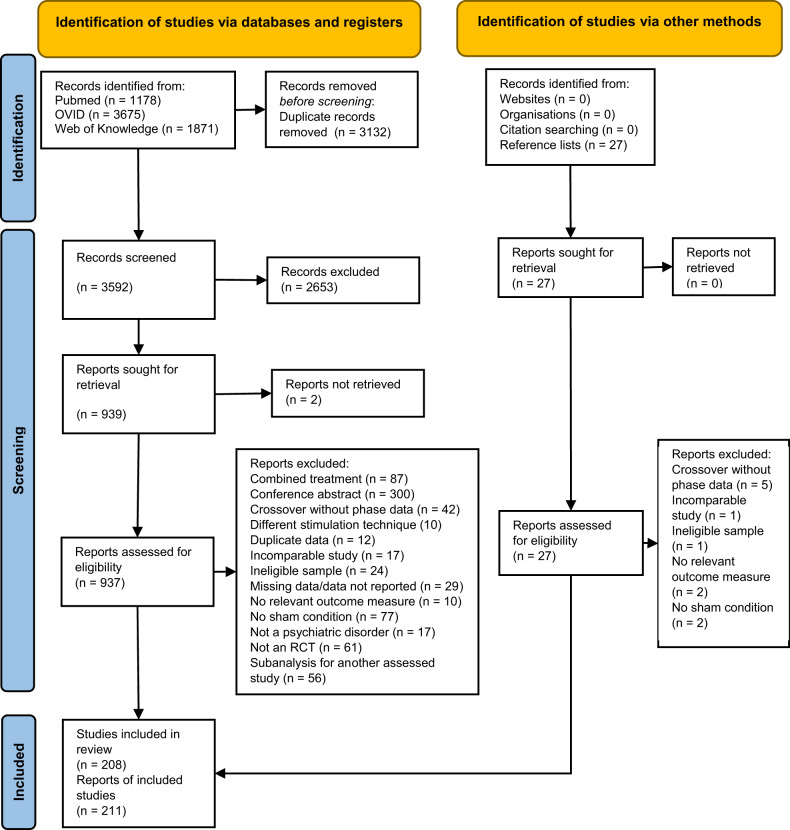
PRISMA flow diagram. The chart illustrates literature search process and how many studies were excluded at each stage.

The systematic search yielded 3592 references from databases and 27 articles from bibliographies. After screening, 208 RCTs reported in 211 articles were included (Fig. 1; study characteristics in Supplementary Table 1). The majority investigated current depressive episodes in patients with major depressive disorder (MDD) or bipolar disorder (*n* = 99), and schizophrenia or schizoaffective disorder (*n* = 59), followed by obsessive-compulsive disorder (OCD, *n* = 27), substance use disorder (SUD, *n* = 10), posttraumatic stress disorder (PTSD, *n* = 8), generalized anxiety disorder (GAD, *n* = 5), attention-deficit/hyperactivity disorder (ADHD, *n* = 2), and tourettes/tic disorders (*n* = 2). For schizophrenia, data were available to assess the efficacy on positive, negative, and total core symptoms, as well as auditory hallucinations. The full list of included and excluded references (with reasons) are reported in the Supplementary Materials.

### Risk of bias

Around 23% RCTs were considered overall high risk of bias, most commonly due to inappropriate analysis (15%) and/or reporting of missing data (16%). Overall, the risk of bias was typically of some concerns (69%), or low (10%; Supplementary Table 2).

### Meta-analyses results-efficacy on core symptoms-continuous outcomes

Active TMS was significantly superior to sham for the treatment of symptoms of depression, GAD, OCD, PTSD, total symptoms, negative symptoms and auditory hallucinations in schizophrenia but not for symptoms of ADHD, SUD, and overall positive symptoms in schizophrenia (Table 1; Supplementary Figs. 1–12, Funnel plots in Supplementary Figs. 13–20). Regarding tDCS, active stimulation was significantly bettter than sham for symptoms of depression, SUD, total, negative symptoms and auditory hallucinations in schizophrenia but not for symptoms of GAD, OCD, and overall positive symptoms in schizophrenia (Table 1; Supplementary Figs. 21–28).

**Table 1:** Summary of the meta-analyses results: core symptoms severity—continuous outcomes.

						Heterogeneity			Egger’s test	
	K	N	SMD (95% CI)	Z	*p* values	Q	*p* values	*I*^2^	*t*	*P* values
*Core symptom severity*										
TMS										
ADHD	2	51	−0.50 (−1.33 to 0.33)	1.18	0.237	2.11	0.146	52		
Depression	76	3366	−0.45 (−0.57 to −0.33)	7.16	<0.001	197.91	<0.001	62	1.95	0.055
Unipolar	42	2336	−0.60 (−0.78 to −0.42)	6.45	<0.001	154.91	<0.001	74	2.85	0.007
Bipolar	4	145	−0.20 (−0.52 to 0.11)	1.26	0.209	1.84	0.606	0		
GAD	3	111	−1.80 (−2.60 to −1.00)	4.40	<0.001	5.37	0.068	63		
OCD	26	760	−0.66 (−0.91 to −0.41)	5.10	<0.001	72.18	<0.001	65	3.31	0.003
PTSD	10	255	−1.09 (−1.61 to −0.57)	4.10	<0.001	42.44	<0.001	79	0.59	0.572
Schizophrenia										
Positive symptoms	33	1474	−0.11 (−0.33 to 0.11)	0.96	0.338	153.20	<0.001	77	2.27	0.029
Negative symptoms	31	1266	−0.49 (−0.73 to −0.26)	4.07	<0.001	133.98	<0.001	78	2.45	0.020
Total symptoms	29	1334	−0.50 (−0.66 to −0.33)	5.81	<0.001	58.67	<0.001	52	2.42	0.022
Auditory hallucinations	16	545	−0.19 (−0.36 to −0.02)	2.19	0.029	12.62	0.632	0	2.64	0.020
SUD	4	100	−1.46 (−3.35 to 0.42)	1.52	0.128	49.44	<0.001	92		
TDCS										
Depression	9	419	−0.87 (−1.51 to −0.24)	2.70	0.007	67.89	<0.001	88		
Unipolar	5	148	−1.04 (−2.17 to 0.08)	1.82	0.069	40.39	<0.001	90		
GAD	2	42	−0.55 (−1.17 to 0.07)	1.74	0.083	0.55	0.457	0		
OCD	2	46	−0.37 (−0.95 to 0.22)	1.23	0.218	0.003	0.953	0		
Schizophrenia										
Positive symptoms	8	367	−0.12 (−0.33 to 0.08)	1.18	0.237	3.59	0.826	0		
Negative symptoms	7	267	−0.54 (−0.95 to −0.14)	2.61	0.009	14.98	0.020	60		
Total symptoms	9	386	−0.63 (−1.03 to −0.23)	3.10	0.002	26.14	0.001	69		
Auditory hallucinations	7	312	−0.42 (−0.81 to −0.02)	2.06	0.040	16.50	0.011	64		
SUD	7	224	−0.73 (−1.00 to −0.46)	5.29	<0.001	2.95	0.815	0		
*Cognitive functioning*										
TMS										
Attention										
Depression	3	146	−0.10 (−0.44 to 0.23)	0.67	0.538	0.97	0.617	0		
Schizophrenia	3	126	−0.18 (−0.64 to 0.29)	0.74	0.457	3.26	0.196	39		
Executive functioning										
Depression	8	292	−0.41 (−0.39 to 0.08)	1.35	0.176	7.46	0.383	6		
Schizophrenia	5	142	−0.28 (−0.74 to 0.18)	1.19	0.233	6.82	0.146	41		
Processing speed										
Depression	7	276	0.07 (−0.17 to 0.31)	0.59	0.553	4.71	0.582	0		
Schizophrenia	5	168	−0.26 (−0.57 to 0.04)	1.70	0.090	1.84	0.765	0		
Working memory										
Depression	7	306	0.02 (−0.21 to 0.25)	0.19	0.848	3.88	0.694	0		
Schizophrenia	10	313	−0.65 (−0.39 to 0.06)	1.42	0.156	9.18	0.421	2	1.86	
SUD	2	69	−0.66 (−1.87 to 0.55)	1.07	0.285	5.95	0.015	83		
TDCS										
Attention										
Schizophrenia	6	247	−0.30 (−0.55 to −0.05)	−2.31	0.021	6.15	0.292	19		
Executive functioning										
Depression	3	154	−0.19 (−0.51 to 0.12)	1.20	0.231	0.12	0.942	0		
Schizophrenia	7	261	−0.13 (−0.37 to 0.12)	1.00	0.317	3.76	0.710	0		
Processing speed										
Depression	3	123	0.05 (−0.31 to 0.41)	0.29	0.771	1.34	0.512	0		
Schizophrenia	7	261	−0.38 (−0.78 to 0.18)	1.87	0.061	14.23	0.027	58		
Working memory										
Depression	5	198	−0.11 (−0.42 to 0.19)	0.73	0.465	4.54	0.338	12		
Schizophrenia	7	279	−0.38 (−0.74 to −0.03)	2.14	0.032	12.24	0.057	51		

*K* number of studies included, *N* overall number of participants, *SMD* standardized mean difference, *ADHD* attention deficit hyperactivity disorder, *GAD* Generalized Anxiety Disorder, *OCD* obsessive-compulsive disorder, *PTSD* Posttraumatic stress disorder, *SUD* substance use disorders, *TMS* transcranial magnetic stimulation, *tDCS* transcranial direct current stimulation.

### Meta-analyses results-efficacy on core symptoms-dicothomous outcomes

Pooled odds ratios for depression response rates showed that there were significantly more responders to active TMS than sham (Table 2).

**Table 2:** Summary of dichotomous outcomes TMS depression meta-analyses results.

						Heterogeneity			Egger’s test	
	K	N	OR (95% CI)	Z	*p* values	Q	*P* values	*I*^2^	*t*	*P* values
Overall	25	1326	3.66 (2.38 to 5.63)	5.90	<0.001	42.94	0.010	44	2.66	0.014
Stimulation type										
BLDLPFC	5	161	3.43 (1.20 to 9.80)	2.30	0.022	11.47	0.022	65		
HF-LDLPFC	6	101	6.03 (1.80 to 20.16)	2.92	0.004	10.81	0.055	54		
HF-VC	2	97	1.68 (0.52 to 5.42)	0.87	0.386	2.02	0.156	50		
iTBS-LDLPFC	4	151	3.84 (1.02 to 14.49)	2.00	0.047	8.16	0.043	63		
TBS-BLDLPFC	2	53	9.15 (3.29 to 25.41)	4.25	<0.001	0.20	0.651	0		
Treatment period										
10	15	754	5.08 (2.97 to 8.71)	5.92	<0.001	22.70	0.065	38	1.50	0.157
15	4	195	4.85 (1.90 to 12.35)	3.31	0.001	1.10	0.777	0		
20	3	190	1.29 (0.35 to 4.79)	0.39	0.700	3.70	0.157	46		
30	3	187	1.75 (0.75 to 4.10)	1.30	0.194	2.62	0.270	24		

*K* number of studies included, *N* overall number of participants, *OR* odds ratio, *BL* bilateral DLPFC stimulation, *HF-L* high frequency left DLPFC stimulation, *HF-VC* high frequency stimulation over the visual cortex, *iTBS-L* intermittent theta-burst stimulation over the left DLPFC, *TBS-BLDLPFC* bilateral DLPFC theta-burst stimulation.

### Meta-analyses results-efficacy on cognitive functioning

We found that active TMS was not superior to sham for cognitive enhancement in any mental condition. tDCS significantly enhanced attention and working memory in patients with schizophrenia (Table 1; Supplementary Figs. 29–44).

A number of analyses were characterized by significant heterogeneity (significant Q test, Tables 1, 2).

### Subgroup analyses for TMS trials of core symptoms

#### Stimulation site and frequency

Across disorders, the most common TMS stimulation site was the unilateral left (L) or right (R) dorsolateral prefrontal cortex (DLPFC) or bilateral DLPFC. For unipolar depression, BLDLPFC and HF-LDLPFC TMS were superior to sham. For OCD, BLDLPFC, LF-RDLPFC and LF supplementary motor area (LF-SMA) TMS were each superior to sham. For PTSD, HF-RDLPFC, and LF-RDLPFC TMS were superior to sham. For schizophrenia negative symptoms HF-LDLPFC was superior to sham (Table 3).

**Table 3:** Summary subgroup meta-analyses: core symptoms, continuous outcome, TMS.

							Heterogeneity			Egger’s test	
	N Sess	K	N	SMD (95% CI)	Z	*p* values	Q	*P* values	*I*^2^	t	*P* values
Depression unipolar											
Stimulation type											
BLDLPFC	15–20	4	206	−0.27 (−0.55 to 0.01)	1.92	0.055	0.63	0.889	0		
HF-LDLPFC	5–30	26	1908	−0.66 (−0.91 to −0.40)	5.01	<0.001	124.85	<0.001	80	2.35	0.028
	5	3	42	−0.23 (−0.80 to 0.33)	0.81	0.418	0.27	0.875	0		
	10	7	228	−0.90 (−1.54 to −0.27)	2.78	0.006	21.24	0.002	72		
	12–15	6	450	−0.46 (−1.01 to 0.09)	1.63	0.103	30.31	<0.001	83		
	20	7	705	−0.46 (−0.78 to −0.15)	2.86	0.004	18.46	0.005	67		
	30	2	105	−2.14 (−4.21 to −0.08)	2.03	0.042	13.94	<0.001	93		
LF-LDLPFC		2	37	−0.01 (−0.68 to 0.66)	0.02	0.985	0.64	0.425	0		
LF-RDLPFC		3	92	−1.35 (−2.47 to −0.22)	2.33	0.020	12.28	0.002	84		
sTMS		2	165	−0.55 (−1.13 to 0.02)	1.89	0.059	3.41	0.061	71		
Bipolar											
HF-LDLPFC		3	99	−0.28 (−0.67 to 0.11)	1.43	0.154	1.37	0.504	0		
OCD											
Stimulation type											
BLDLPFC	10–20	4	87	−1.10 (−1.54 to −0.65)	4.80	<0.001	1.93	0.059	0		
HF-LDLPFC	10–15	2	58	−0.13 (−0.62 to 0.36)	0.52	0.604	0.00	0.955	0		
HF-RDLPFC	10–30	3	99	−0.65 (−1.48 to 0.18)	1.54	0.124	8.50	0.014	76		
LF-RDLPFC	10–20	4	95	−0.54 (−1.00 to −0.18)	2.83	0.005	3.23	0.352	8		
LF-SMA	10–25	6	183	−1.24 (−2.23 to −0.25)	2.46	0.014	43.92	<0.001	89		
PTSD											
Stimulation type											
HF-RDLPFC	10	3	73	−1.32 (−2.50 to −0.14)	2.20	0.028	10.04	0.007	80		
LF-RDLPFC	10–15	4	70	−0.83 (−1.43 to −0.23)	2.72	0.006	4.90	0.179	38		
Schizophrenia negative symptoms											
Stimulation type											
BLDLPFC		3	77	0.06 (−0.39 to 0.50)	0.24	0.811	1.36	0.507	0		
HF-LDLPFC	5–40	19	1029	−0.61 (−0.92 to −0.30)	3.86	<0.001	96.74	<0.001	81	3.06	0.007
	5	3	107	−0.23 (−0.61 to 0.16)	1.16	0.244	1.00	0.605	0		
	10	2	139	−0.11 (−0.46 to 0.24)	0.62	0.537	1.00	0.318	0		
	15	5	284	−0.83 (−1.42 to −0.25)	2.79	0.005	17.07	0.002	77		
	20	6	319	−1.12 (−1.99 to −0.25)	2.52	0.012	60.44	<0.001	92		
	40	3	185	−0.10 (−0.39 to 0.19)	0.68	0.495	0.23	0.892	0		
LF-LDLPFC	10	2	43	−0.32 (0.92 to 0.29)	1.02	0.307	0.81	0.368	0		

*K* number of studies included, *N* overall number of participants, *SMD* standardized mean difference, *OCD* obsessive-compulsive disorder, *PTSD* Posttraumatic stress disorder, *TMS* transcranial magnetic stimulation, *sTMS* synchronized TMS, *BLDLPFC* dorsolateral prefrontal cortex, *LDLPFC* left dorsolateral prefrontal cortex, *RDLPFC* right dorsolateral prefrontal cortex, *SMA* supplementary motor area, *HF* high-frequency stimulation (≥5 Hz), *LF* low-frequency stimulation (≤1 Hz).

#### Number of treatment sessions

For unipolar depression, HF-LDLPFC trials of 10, 20 and 30 sessions were superior to sham. For OCD, BLDLPFC, and LF-RDLPFC trials of 10–20 treatment sessions and LF-SMA trials of 10–25 sessions were superior to sham. For PTSD, 10–20 sessions of LF-RDLPFC were superior to sham. For schizophrenia, HF-LDLPFC trials of 10, 15, and 20 sessions were superior to sham (Table 3).

#### Sensitivity analyses of core symptoms

Sensitivity analyses excluding high risk of bias RCTs did not show substantial differences for the core symptom domains. The only change was the finding of TMS inducing significant improvements in executive functioning in depression (Table 4).

**Table 4:** Sensitivity analyses of continuous outcome primary meta-analyses results.

						Heterogeneity			Egger’s test	
	K	N	SMD (95% CI)	Z	*p* values	Q	*p* values	*I*^2^	*t*	*P* values
*Core symptom severity*										
TMS										
Depression^a^	56	1877	−0.44 (−0.56 to −0.31)	6.94	<0.001	204.04	<0.001	63	1.73	0.088
GAD^a^	2	86	−1.95 (−3.11 to −0.80)	3.31	0.001	4.53	0.033	78		
OCD^a^	18	536	−0.65 (−0.95 to −0.36)	4.28	<0.001	48.18	<0.001	65	1.89	0.078
PTSD^a^	8	225	−1.03 (−1.61 to −0.45)	3.47	0.001	40.00	<0.001	82		
Schizophrenia										
Positive symptoms^a^	33	1242	−0.24 (−0.51 to 0.03)	1.75	0.080	147.45	<0.001	81	2.49	0.019
Negative symptoms^a^	24	1034	−0.54 (−0.84 to −0.24)	3.48	0.001	125.85	<0.001	82	2.24	0.036
Total symptoms^a^	29	1024	−0.52 (−0.72 to −0.32)	5.15	<0.001	50.41	0.001	56	1.96	0.063
Hallucinations^a^	13	459	−0.21 (−0.39 to −0.03)	2.32	0.021	10.75	0.550	0	3.71	0.003
SUD	4	100	−1.46 (−3.35 to 0.42)	1.52	0.128	49.44	<0.001	92		
TDCS										
Depression^a^	8	355	−0.59 (−1.32 to 0.15)	1.56	0.118	72.33	<0.001	90		
GAD	2	42	−0.55 (−1.17 to 0.07)	1.74	0.083	0.55	0.457	0		
OCD	2	46	−0.37 (−0.95 to 0.22)	1.23	0.218	0.003	0.953	0		
Schizophrenia										
Positive symptoms	8	367	−0.12 (−0.33 to 0.08)	1.18	0.237	3.59	0.826	0		
Negative symptoms	6	252	−0.44 (−0.82 to −0.06)	2.26	0.024	11.11	0.049	55		
Total symptoms^a^	8	371	−0.55 (−0.94 to −0.15)	2.73	0.006	22.94	0.002	69		
Hallucinations	7	312	−0.42 (−0.81 to −0.02)	2.06	0.040	16.50	0.011	64		
SUD^a^	5	174	−0.44 (−0.79 to −0.10)	2.55	0.011	2.95	0.815	0		
*Cognitive functioning*										
TMS										
Attention										
Depression^a^	2	62	−0.05 (−0.45 to 0.56)	0.21	0.837	0.37	0.545	0		
Schizophrenia^a^	2	101	0.03 (−0.39 to 0.44)	0.12	0.906	1.13	0.288	11		
Executive functioning										
Depression^b^	7	229	−0.27 (−0.53 to 0.01)	2.05	0.040	4.18	0.652	0		
Schizophrenia^a^	3	96	0.08 (−0.31 to 0.47)	0.40	0.688	0.68	0.713	0		
Processing speed										
Depression^a^	6	213	0.04 (−0.18 to 0.26)	0.39	0.700	5.75	0.570	0		
Schizophrenia^a^	4	143	−0.25 (−0.59 to 0.08)	1.51	0.132	1.96	0.580	0		
Working memory										
Depression^a^	5	159	0.002 (−0.33 to 0.33)	0.01	0.991	4.29	0.368	7		
Schizophrenia^a^	8	267	−0.12 (−0.38 to 0.15)	0.88	0.381	8.07	0.326	13		
SUD	2	69	−0.66 (−1.87 to 0.55)	1.07	0.285	5.95	0.015	83		
TDCS										
Attention										
Schizophrenia	6	247	−0.30 (−0.55 to −0.05)	−2.31	0.021	6.15	0.292	19		
Executive functioning										
Depression	3	154	−0.19 (−0.51 to 0.12)	1.20	0.231	0.12	0.942	0		
Schizophrenia	7	261	−0.13 (−0.37 to 0.12)	1.00	0.317	3.76	0.710	0		
Processing speed										
Depression	3	123	0.05 (−0.31 to 0.41)	0.29	0.771	1.34	0.512	0		
Schizophrenia	7	261	−0.38 (−0.78 to −0.18)	1.87	0.061	14.23	0.027	58		
Working memory										
Depression	5	198	−0.11 (−0.42 to 0.19)	0.73	0.465	4.54	0.338	12		
Schizophrenia	7	279	−0.38 (−0.74 to −0.03)	2.14	0.032	12.24	0.057	51		

*GAD* generalized anxiety disorder, *OCD* obsessive-compulsive disorder, *PTSD* posttraumatic stress disorder, *SUD* substance use disorders, *TMS* transcranial magnetic stimulation, *tDCS* transcranial direct current stimulation.

^a^Re-calculated with papers including at least one “high risk” category removed.

^b^Change to significance.

Again a number of results were limited by significant heterogeneity (Tables 1, 2).

## Discussion

We conducted the first series of meta-analyses of RCTs investigating the efficacy of NIBS for the treatment of core symptoms and improvement of cognitive functioning using the same criteria within a broad range of mental disorders. We found that TMS and tDCS had significant effects on the core symptom severity of several disorders, although significant heterogeneity limits the confidence of some results. We discuss here the effects of NIBS grouped by mental disorder.

In line with previous evidence synthesis [22], TMS significantly reduced GAD symptoms, with a large effect size and no significant heterogeneity. However, only three RCTs were available for analysis. Each study utilized a different stimulation protocol, with high-frequency right DLPFC (HF-R), low-frequency right DLPFC (LF-R), and low-frequency right parietal stimulation, all producing significant positive effects. Therefore, despite this positive finding, more studies are needed to better understand the therapeutic mechanisms and optimal treatment parameters of NIBS for GAD.

PTSD symptom severity significantly decreased following rTMS, yielding large effect sizes and significant improvements in the majority of RCTs, albeit with significant heterogeneity. As in previous reports [23], LF-RDLPFC rTMS yielded a significant effect without heterogeneity, indicating robust symptom improvement.

OCD symptoms were significantly reduced with TMS, with a medium effect size. Although heterogeneity for the overall findings was high, seven RCTs showed significant improvements of OCD symptoms. When taking into account different stimulation parameters, BLDLPFC rTMS produced the largest effect size without significant heterogeneity, indicating robust symptom improvement. Consistent with a previous analysis [24], LF-RDLPFC rTMS was also independently effective, whereas HF-RDLPFC stimulation was not. However, the low number of available RCTs suggests that these results should be considered cautiously. In line with previous literature, low-frequency SMA stimulation yielded large and significant effects but no robust conclusions can be drawn due to high heterogeneity [24].

Overall, TMS was effective in treating depressive episodes with a medium effect size, but high heterogeneity. Notably, when depressive episodes were split by polarity, TMS was effective in treating unipolar but not bipolar depression although for the latter there were only four available RCTs. Considering stimulation parameters as a possible source of variability across unipolar depression TMS studies, our subgroup analysis on BLDLPFC stimulation yielded a small but consistent effect, with no significant heterogeneity. A subgroup analysis on LF-RDLPFC stimulation also yielded a positive effect, although this was characterized by significant heterogeneity and comprised only three studies. In addition, we also found a positive effect of HF-LDLPFC stimulation, which was the most commonly implemented stimulation in RCTs for depression overall (*n* = 46). HF-LDLPFC rTMS has been shown to increase activity in the left PFC [25], an area that shows abnormal activity in patients with MDD [26]. Accordingly, large HF-LDLPFC TMS RCTs have found significant antidepressant effects [27, 28], and this stimulation type is currently recommended by treatment guidelines [29]. However, in our subgroup analysis on HF-LDLPFC stimulation for unipolar depression, heterogeneity remained significant, suggesting that the efficacy cannot be assumed to be robust across studies or participants, although heterogeneous findings should be expected with a large number of studies. Overall, the results of the current meta-analysis suggest that BLDLPFC rTMS might have a more consistent, small effect on symptoms of unipolar depression, while HF-LDLPFC stimulation could achieve larger effects but with more variability. Notably, effect sizes for tDCS overall depression symptom improvement were higher than those reported in previous studies [30–32], and are comparable to those recently reported for psycho- and pharmacotherapies [33, 34]. However, while the effect sizes are promising, the results should be interpreted with caution due to high heterogeneity.

In patients with schizophrenia, NIBS did not appear effective for reducing overall positive symptoms. Negative symptoms were significantly improved by both TMS and tDCS protocols with a medium effect size. However, these results were characterized by high heterogeneity and therefore some caution is warranted when interpreting the results of NIBS on negative symptoms in schizophrenia. For auditory hallucinations, tDCS yielded a small positive effect but with high heterogeneity and TMS yielded a negligible but homogenous positive effect. These findings were similar to a previous study found that TMS was effective for auditory hallucinations but not overall positive symptoms, which could even worsen [35]. The most common cortical targets for schizophrenia are the left DLPFC and the left temporoparietal junction (TPJ). Dysfunctional PFC activation has been associated with negative symptoms and cognitive deficits [36], and auditory hallucinations are thought to originate from spontaneous activity in hyperactive temporal regions, which is not adequately inhibited due to prefrontal hypoactivity [37]. Accordingly, our subgroup analyses showed that TMS protocols targeting hallucinations typically employed low-frequency stimulation over the left TPJ [38–45], while those targeting negative symptoms commonly used HF-LDLPFC stimulation [46–50]. Typically, tDCS protocols positioned the anode to excite neural activity in the left DLPFC and the cathode to inhibit neural activity in the left TPJ, which allowed both negative symptoms and hallucinations to be targeted [51–55]. In summary, it appears that neurostimulation could be used in patients with schizophrenia, especially for negative symptoms.

In the current analysis, TMS was not effective for the treatment of SUD symptoms but tDCS yielded a large effect size without significant heterogeneity. This is in contrast with a previous study that found both tDCS and TMS to be effective [56]. However, in the previous study, TMS and tDCS were grouped, and several TMS RCTs were excluded from the current analysis due to lacking a formal diagnosis [57], absent sham condition [58], non-standardized symptom measurement [59] and crossover designs without report of first phase data [60–62]. The reliable finding for tDCS as a treatment for SUD shows promise but was based on seven RCTs, so more trials are needed to draw robust conclusions.

We did not find TMS to be superior to sham for ADHD core symptoms. This is in line with a recent meta-analysis that found no effect of TMS or tDCS on ADHD clinical and cognitive symptoms [63]. The present analysis of ADHD was limited by RCT availability, and the exclusion of studies combining NIBS with cognitive training (CT). As per protocol, studies combining NIBS with CT were excluded from our analysis, as the focus was specifically on NIBS, and disentangling the specific contribution of NIBS when combined with CT would be challenging. However, while there is some evidence that the positive effects of tDCS can be enhanced when combined with CT by priming the brain regions that mediate the cognitive function being trained [64, 65], recent well-designed RCTs have not replicated these findings in ADHD [66]. Further RCTs combining tDCS and CT for ADHD should be conducted, to allow future meta-analyses assessing the potential of combining tDCS with CT for individuals with ADHD.

Finally, two RCTs investigating the efficacy of TMS for tic disorders were included in the systematic review but not the meta-analysis. One RCT was in adults [67], the other in children and young adults [68]. Both trials used inhibitory TMS over the supplementary motor area (SMA) but neither found positive effects of TMS on tic symptoms. Several small open label studies have found positive effects of low fequency TMS over the SMA for tic symptoms [69–72], but these findings have not yet been replicated in RCTs.

In terms of the impact of the duration of treatment, within disorder subgroup analyses of stimulation protocols with sufficient studies suggested that 10–20 sessions of TMS was typically most effective for reducing symptoms. Longer trial durations did not appear to increase effect sizes, although within most disorders the number of trials with more than ten sessions was limited.

Regarding improvement in cognitive functioning, small to moderate effect sizes without significant heterogeneity were found for tDCS on attention and working memory performance in patients with schizophrenia, and executive functioning performance in patients with depression after sensitivity analyses. RCTs for schizophrenia are of particular importance, as a previous evidence synthesis of pharmacological treatments for cognitive deficits in schizophrenia showed limited efficacy [73]. The effects for all other cognitive domains across disorders were either non-significant or could not be calculated due to lack of data in the studies. Overall, these results are consistent with a previous meta-analysis investigating the effects of NIBS on several cognitive functioning domains across several mental health disorders [18], with limited evidence for the overall effectiveness of NIBS on cognitive functioning but potential for positive effects on attention and working memory. A possible explanation for this finding may be provided by recent empirical evidence demonstrating that bifrontal tDCS can increase dopamine release in the ventral striatum [74]. It is hypothesized that dopamine activity in the striatum has associations with prefrontal functioning and more specifically with higher-order cognition including working memory updating and attention shifting [75]. Thus, increased dopamine activity in this area could be a potential mechanism behind these positive tDCS effects. However, we did not find any positive effect of tDCS for other cognitive domains across mental disorders, suggesting that tDCS could only be used to target specific cognitive functions in specific patient groups.

## Strengths and limitations

Strengths of the current meta-analysis are the comprehensive search strategy, no limitations in language or type of document, the inclusion of unpublished infomation/data gathered by study authors, and inclusion of the most rigorous study design (RCT) only. The overall quality of included RCTs was also good. Although many RCTs were of “some concerns” according to the RoB2, this was due largely to a lack of a prespecified analysis plan rather than issues with RCT design. Another strength is the exploration of subgroups based on stimulation site and number of sessions, which allowed for identification of particularly strong treatment paradigms. Furthermore, only studies with formally diagnosed patients and those involving NIBS as a monotherapy or augmentation of stable treatment were retained, thus avoiding heterogeneity related to different diagnostic methods and confounding effects of additional therapies. However, although the effects of each treatment are difficult to disentangle in combined trials, previous studies have shown that NIBS can be more effective when co-initiated with other treatments such as pharmacotherapy [76], cognitive therapies [77, 78], exposure-based therapy [79] or cognitive training [64, 65]. Thus, our analysis was also limited by the exclusion of combined trials. We suggest that future RCTs should consider neurotherapies combined with other strategies, particularly in individuals with anxiety-related disorders, who can benefit from exposure-based techniques [80] and in those with ADHD, who can benefit from cognitive training in terms of improvement in some executive functions [81]. Furthermore, it should be noted that, given the nature of available data, we could not control for the effect of concomitant medication. Therefore, our results should be interpreted with caution, especially in relation to trials in individuals with schizophrenia, in which the majority of patients were medicated during the course of neurotherapy. Our meta-analysis was also limited by the small number of available RCTs for some disorders and stimulation types. For example, no subgroup analyses could be run within ADHD or GAD due to a lack of studies, and fewer than 10 tDCS RCTs were available for any mental disorder. Furthermore, as it is recommended to conduct meta-regression analyses with at least ten studies per regressor [82], and data on potential regressors were not consistently reported across studies, it was not possible to explore the planned regressors. We also planned to include RCTs in children/adolescents however, all the retained studies were in adults, which prevented us from assessing possible developmental differences in efficacy. We also could not examine the longer term effects of NIBS as data from follow-up periods were not analyzed. Additionally, funnel plots showed the possibility of some publication bias in the results regarding TMS in patients with OCD and schizophrenia. Finally, for the present report, our primary analysis was based on standardized mean difference as this was calculable from data provided by the majority of included studies. We did not plan to extract any data pertaining to NIBS safety, tolerability or individual patient responses as this was beyond the scope of the present study. Both TMS and tDCS have been widely reported as safe techniques with minimal adverse effects [83, 84], and as such the focus of this study was on efficacy. However, future studies should consider analysis of individual patient data as this would provide a reliable assessment of the percentage of responders and the acceptability of NIBS across mental disorders.

## Conclusions

Overall, TMS was found to be superior to sham for GAD, and tDCS to be superior to sham for cravings in SUD. We also found significant medium to large effect sizes for TMS for reducing symptom severity of unipolar and overall depressive episodes, OCD, PTSD, and negative symptoms in patients with schizophrenia and TDCS for reducing symptom severity of overall depressive episodes, auditory hallucinations, and negative symptoms of schizophrenia. However, these results were characterized by significant heterogeneity, so must be interpreted with caution. In contrast to TMS, tDCS was effective for the enhancement of attention and working memory in patients with schizophrenia. In order to be most effective, TMS should entail 10–20 sessions. Further high quality NIBS trials are needed within understudied disorders and novel stimulation techniques. Additionally, further exploration of heterogeneity among trials within well-researched disorders is warranted to identify sources of variability in treatment effects.

## Supplementary information

Supplementary Material
