# Supplementary material for: Efficacy of neurostimulation across mental disorders: systematic review and meta-analysis of 208 randomized controlled trials
Source: Mol Psychiatry. 2022 Apr 1;27(6):2709–19. doi: 10.1038/s41380-022-01524-8 (PMC8973679; doi:10.1038/s41380-022-01524-8)
Supplement: Supplementary file 1 — Supplementary Material [file 41380_2022_1524_MOESM1_ESM.docx]

**Supplementary Information**

**Supplementary material**

1. Search Terms
2. Full list of included references
3. Full list of excluded references with reasons
4. Changes to pre-registered protocol and post-hoc analyses

**Supplementary Table 1.** Study characteristics of included randomized controlled trials

**Supplementary Table 2.** Risk of bias assessments

**Supplementary Figures 1-12.** Forest plots showing effect sizes of TMS for symptom severity

**Supplementary Figures 13-20.** Funnel plots showing publication bias in TMS trials of symptom severity

**Supplementary Figures 21-28.** Forest plots showing effect sizes of tDCS for symptom severity

**Supplementary Figures 29-44.** Forest plots showing effect sizes of NIBS for cognitive functioning

**Supplementary material A.** Search Terms

**PUBMED (MEDLINE)**

- Search terms:

(ADHD [tiab] OR Attention-Deficit [tiab] OR attention deficit [tiab] OR depress* [tiab] OR anxiety [tiab] OR anxious [tiab] OR obsessive compulsive [tiab] OR OCD [tiab] OR panic disorder* [tiab] OR psychotic [tiab] OR psychosis [tiab] OR schizophren* [tiab] OR tic [tiab] OR tics [tiab] OR Tourette [tiab] OR Tourette’s [tiab]) OR (Intellectual Disability [tiab] or intellectual developmental disorder [tiab] or global developmental delay [tiab] or autis* [tiab] or aperger* [tiab] or schizotypal or delusional disorder* [tiab] or bipolar [tiab] or cyclothymic [tiab] or mood disorder* [tiab] or mood dysregulation disorder [tiab] or Agoraphobia [tiab] or Body Dysmorphic Disorder* [tiab] or Hoarding Disorder [tiab] or Trichotillomania [tiab] or Hair-Pulling Disorder [tiab] or Hair-Pulling Disorder* [tiab] or Excoriation disorder* [tiab] or Skin-Picking Disorder* [tiab] or Posttraumatic Stress Disorder* [tiab] or PTSD [tiab] or Acute Stress Disorder* [tiab] or Dissociative Identity Disorder* [tiab] or Somatic Symptom Disorder [tiab] or Illness Anxiety Disorder* [tiab] or Conversion Disorder* [tiab] or Functional Neurological Symptom Disorder[tiab] or Anorexia Nervosa [tiab] or Bulimia Nervosa[tiab] or Binge-Eating Disorder* [tiab] or Binge Eating Disorder* [tiab] or enuresis [tiab] or encopresis [tiab] or sleep disorder* [tiab] or insomnia [tiab] or restless legs syndrome [tiab] or RLS [tiab] or personality disorder* [tiab] or gender dysphoria [tiab] or neurocognitive disorder* or paraphilic disorder* [tiab] or Voyeuristic Disorder* [tiab] or Exhibitionistic Disorder* [tiab] or Frotteuristic Disorder [tiab] or Sexual Masochism Disorder* [tiab] or Sexual Sadism Disorder* [tiab] or Pedophilic Disorder* [tiab] or Fetishistic Disorder* [tiab] or language disorder [tiab] or speech and sound disorder [tiab] or Childhood-Onset Fluency Disorder [tiab] or stuttering [tiab] or Social Communication Disorder [tiab] or Pragmatic Communication Disorder [tiab] or Specific learning disorder* [tiab] or dyslexia [tiab] or dyscalculia [tiab] or Motor skills disorder* or Developmental coordination disorder* [tiab] or schizoaffective [tiab] or selective mutism [tiab] or phobia [tiab] or Reactive Attachment Disorder [tiab] or Disinhibited Social Engagement Disorder [tiab] or pica [tiab] or Rumination Disorder [tiab] or Avoidant/Restrictive Food Intake Disorder [tiab] or parasomnia* [tiab] or sleepwalking [tiab] or nightmare disorder [tiab] or sleep terror* [tiab] or Oppositional defiant disorder or conduct disorder [tiab] or Intermittent Explosive Disorder* [tiab] or substance use disorder [tiab] or addictive disorder [tiab]) AND (random* [tiab])  AND (rTMS [tiab] OR repetitive transcranial magnetic stimulation [tiab] or tDCS[tiab] OR transcranial direct current stimulation [tiab])

Limits: **none**

**OVID databases: PsycInfo, EMBASE+EMBASE classic, OVID Medline**

Search terms:

(ADHD OR Attention-Deficit OR attention deficit OR depression OR depressed OR anxiety OR anxious OR obsessive compulsive OR OCD OR panic disorder OR psychotic OR psychosis OR schizophrenia OR schizophrenic OR tic OR tics OR Tourette) OR (Intellectual Disability or intellectual developmental disorder or global developmental delay or autis* or aperger* or schizotypal or delusional disorder* or bipolar or cyclothymic or mood disorder* or mood dysregulation disorder or Agoraphobia or Body Dysmorphic Disorder* or Hoarding Disorder or Trichotillomania or Hair Pulling Disorder or Excoriation disorder* or Skin Picking Disorder* or Posttraumatic Stress Disorder* or PTSD or Acute Stress Disorder* or Dissociative Identity Disorder* or Somatic Symptom Disorder or Illness Anxiety Disorder* or Conversion Disorder* or Functional Neurological Symptom Disorder or Anorexia Nervosa or Bulimia Nervosa or Binge Eating Disorder* or enuresis or encopresis or sleep disorder* or insomnia or restless legs syndrome or RLS or personality disorder* or gender dysphoria or neurocognitive disorder* or paraphilic disorder* or Voyeuristic Disorder* or Exhibitionistic Disorder* or Frotteuristic Disorder or Sexual Masochism Disorder* or Sexual Sadism Disorder* or Pedophilic Disorder* or Fetishistic Disorder* or language disorder or speech and sound disorder or Childhood Onset Fluency Disorder or stuttering or Social Communication Disorder or Pragmatic Communication Disorder or Specific learning disorder* or dyslexia or dyscalculia or Motor skills disorder* or Developmental coordination disorder* or schizoaffective or selective mutism or phobia or Reactive Attachment Disorder or Disinhibited Social Engagement Disorder or pica or Rumination Disorder or Avoidant Restrictive Food Intake Disorder or parasomnia* or sleepwalking or nightmare disorder or sleep terror* or Oppositional defiant disorder or conduct disorder or Intermittent Explosive Disorder* or substance disorder or addictive disorder ) AND (random OR randomized OR randomised) AND (rTMS  OR repetitive transcranial magnetic stimulation OR tDCS OR transcranial direct current stimulation) OR

Limits: **none**

**WEB OF KNOWLEDGE: (Web of science (science citation index expanded), Biological abstracts, Biosis, Food science and technology abstracts)**

Search terms:

ADHD OR Attention-Deficit OR attention deficit OR depression OR depressed OR anxiety OR anxious OR obsessive compulsive OR OCD OR panic disorder OR psychotic OR psychosis OR schizophrenia OR schizophrenic OR tic OR tics OR Tourette OR Intellectual Disability or intellectual developmental disorder or global developmental delay or autis* or aperger* or schizotypal or delusional disorder* or bipolar or cyclothymic or mood disorder* or mood dysregulation disorder or Agoraphobia or Body Dysmorphic Disorder* or Hoarding Disorder or Trichotillomania or Hair Pulling Disorder or Excoriation disorder* or Skin Picking Disorder* or Posttraumatic Stress Disorder* or PTSD or Acute Stress Disorder* or Dissociative Identity Disorder* or Somatic Symptom Disorder or Illness Anxiety Disorder* or Conversion Disorder* or Functional Neurological Symptom Disorder or Anorexia Nervosa or Bulimia Nervosa or Binge Eating Disorder* or enuresis or encopresis or sleep disorder* or insomnia or restless legs syndrome or RLS or personality disorder* or gender dysphoria or neurocognitive disorder* or paraphilic disorder* or Voyeuristic Disorder* or Exhibitionistic Disorder* or Frotteuristic Disorder or Sexual Masochism Disorder* or Sexual Sadism Disorder* or Pedophilic Disorder* or Fetishistic Disorder* or language disorder or speech and sound disorder or Childhood Onset Fluency Disorder or stuttering or Social Communication Disorder or Pragmatic Communication Disorder or Specific learning disorder* or dyslexia or dyscalculia or Motor skills disorder* or Developmental coordination disorder* or schizoaffective or selective mutism or phobia or Reactive Attachment Disorder or Disinhibited Social Engagement Disorder or pica or Rumination Disorder or Avoidant Restrictive Food Intake Disorder or parasomnia* or sleepwalking or nightmare disorder or sleep terror* or Oppositional defiant disorder or conduct disorder or Intermittent Explosive Disorder* or substance disorder or addictive disorder

AND

rTMS  OR repetitive transcranial magnetic stimulation OR tDCS OR transcranial direct current stimulation

random*

AND

rTMS OR repetitive transcranial magnetic stimulation or tDCS OR transcranial direct current stimulation

AND

random OR randomized OR randomised

rTMS  OR repetitive transcranial magnetic stimulation OR tDCS OR transcranial direct current stimulation

Limits: **none**

**Supplementary material B.** Full list of included references

1. Ahmadizadeh MJ, Rezaei M. Unilateral right and bilateral dorsolateral prefrontal cortex transcranial magnetic stimulation in treatment post-traumatic stress disorder: A randomized controlled study. *Brain Res Bull* 2018; **140:** 334-340.

2. Ahmadizadeh MJ, Rezaei M, Fitzgerald PB. Transcranial direct current stimulation (tDCS) for post-traumatic stress disorder (PTSD): A randomized, double-blinded, controlled trial. *Brain Res Bull* 2019; **153:** 273-278.

3. Alizadehgoradel J, Nejati V, Movahed FS, Imani S, Taherifard M, Mosayebi-Samani M *et al.* Repeated stimulation of the dorsolateral-prefrontal cortex improves executive dysfunctions and craving in drug addiction: A randomized, double-blind, parallel-group study. *Brain Stimulation* 2020; **13**(3)**:** 582-593.

4. Alonso P, Pujol J, Cardoner N, Benlloch L, Deus J, Menchon JM *et al.* Right prefrontal repetitive transcranial magnetic stimulation in obsessive-compulsive disorder: a double-blind, placebo-controlled study. *American Journal of Psychiatry* 2001; **158**(7)**:** 1143-1145.

5. Alyagon U, Shahar H, Hadar A, Barnea-Ygael N, Lazarovits A, Shalev H *et al.* Alleviation of ADHD symptoms by non-invasive right prefrontal stimulation is correlated with EEG activity. *Neuroimage Clin* 2020; **26:** 102206.

6. Anderson IM, Delvai NA, Ashim B, Ashim S, Lewin C, Singh V *et al.* Adjunctive fast repetitive transcranial magnetic stimulation in depression. *British Journal of Psychiatry* 2007; **190:** 533-534.

7. Arumugham SS, Subhasini VS, Madhuri HN, Vinay B, Ravi M, Sharma E *et al.* Augmentation Effect of Low-Frequency Repetitive Transcranial Magnetic Stimulation Over Presupplementary Motor Area in Obsessive-Compulsive Disorder A Randomized Controlled Trial. *J Ect* 2018; **34**(4)**:** 253-257.

8. Avery DH, Claypoole K, Robinson L, Neumaier JF, Dunner DL, Scheele L *et al.* Repetitive transcranial magnetic stimulation in the treatment of medication-resistant depression: preliminary data. *Journal of Nervous & Mental Disease* 1999; **187**(2)**:** 114-117.

9. Avery DH, Holtzheimer IPE, Fawaz W, Russo J, Neumaier J, Dunner DL *et al.* A controlled study of repetitive transcranial magnetic stimulation in medication-resistant major depression. *Biological Psychiatry* 2006; **59**(2)**:** 187-194.

10. Badawy AA, El Sawy H, El Hay M. Efficacy of repetitive transcranial magnetic stimulation in the management of obsessive compulsive disorder. *Egyptian Journal of Neurology, Psychiatry and Neurosurgery* 2010; **47:** 393-397.

11. Baeken C, Vanderhasselt M-A, Remue J, Herremans S, Vanderbruggen N, Zeeuws D *et al.* Intensive HF-rTMS treatment in refractory medication-resistant unipolar depressed patients. *Journal of Affective Disorders* 2013; **151**(2)**:** 625-631.

12. Bais L, Vercammen A, Stewart R, van Es F, Visser B, Aleman A *et al.* Short and long term effects of left and bilateral repetitive transcranial magnetic stimulation in schizophrenia patients with auditory verbal hallucinations: a randomized controlled trial. *PLoS One* 2014; **9**(10)**:** e108828.

13. Bakim B, Uzun UE, Karamustafalioglu O, Ozcelik B, Alpak G, Tankaya O *et al.* The Combination of Antidepressant Drug Therapy and High-Frequency Repetitive Transcranial Magnetic Stimulation in Medication-Resistant Depression. *Klin Psikofarmakol Bul* 2012; **22**(3)**:** 244-253.

14. Barr MS, Farzan F, Arenovich T, Chen R, Fitzgerald PB, Daskalakis ZJ. The effect of repetitive transcranial magnetic stimulation on gamma oscillatory activity in schizophrenia. *PLoS ONE [Electronic Resource]* 2011; **6**(7)**:** e22627.

15. Barr MS, Farzan F, Rajji TK, Voineskos AN, Blumberger DM, Arenovich T *et al.* Can repetitive magnetic stimulation improve cognition in schizophrenia? Pilot data from a randomized controlled trial. *Biol Psychiatry* 2013; **73**(6)**:** 510-517.

16. Barr MS, Farzan F, Tran LC, Fitzgerald PB, Daskalakis ZJ. A randomized controlled trial of sequentially bilateral prefrontal cortex repetitive transcranial magnetic stimulation in the treatment of negative symptoms in schizophrenia. *Brain Stimul* 2012; **5**(3)**:** 337-346.

17. Bation R, Mondino M, Le Camus F, Saoud M, Brunelin J. Transcranial direct current stimulation in patients with obsessive compulsive disorder: A randomized controlled trial. *Eur Psychiatry* 2019; **62:** 38-44.

18. Batista EK, Klauss J, Fregni F, Nitsche MA, Nakamura-Palacios EM. A Randomized Placebo-Controlled Trial of Targeted Prefrontal Cortex Modulation with Bilateral tDCS in Patients with Crack-Cocaine Dependence. *Int J Neuropsychopharmacol* 2015; **18**(12).

19. Berman RM, Narasimhan M, Sanacora G, Miano AP, Hoffman RE, Hu XS *et al.* A randomized clinical trial of repetitive transcranial magnetic stimulation in the treatment of major depression. *Biol Psychiatry* 2000; **47**(4)**:** 332-337.

20. Beynel L, Chauvin A, Guyader N, Harquel S, Bougerol T, Marendaz C *et al.* What saccadic eye movements tell us about TMS-induced neuromodulation of the DLPFC and mood changes: A pilot study in bipolar disorders. *Frontiers in Integrative Neuroscience* 2014; **8**(AUG).

21. Blumberger D, Tran L, Fitzgerald P, Hoy KB, Daskalakis ZJ. A randomized double-blind sham-controlled study of transcranial direct current stimulation for treatment-resistant major depression. *Front Psychiatry* 2012; **3:** 74.

22. Blumberger DM, Christensen BK, Zipursky RB, Moller B, Chen R, Fitzgerald PB *et al.* MRI-targeted repetitive transcranial magnetic stimulation of Heschl's gyrus for refractory auditory hallucinations. *Brain Stimul* 2012; **5**(4)**:** 577-585.

23. Blumberger DM, Maller JJ, Thomson L, Mulsant BH, Rajji TK, Maher M *et al.* Unilateral and bilateral MRI-targeted repetitive transcranial magnetic stimulation for treatment-resistant depression: a randomized controlled study. *J Psychiatry Neurosci* 2016; **41**(4)**:** E58-66.

24. Blumberger DM, Mulsant BH, Fitzgerald PB, Rajji TK, Ravindran AV, Young LT *et al.* A randomized double-blind sham-controlled comparison of unilateral and bilateral repetitive transcranial magnetic stimulation for treatment-resistant major depression. *World J Biol Psychiatry* 2012; **13**(6)**:** 423-435.

25. Bortolomasi M, Minelli A, Fuggetta G, Perini M, Comencini S, Fiaschi A *et al.* Long-lasting effects of high frequency repetitive transcranial magnetic stimulation in major depressed patients. *Psychiatry Res* 2007; **150**(2)**:** 181-186.

26. Bose A, Shivakumar V, Agarwal SM, Kalmady SV, Shenoy S, Sreeraj VS *et al.* Efficacy of fronto-temporal transcranial direct current stimulation for refractory auditory verbal hallucinations in schizophrenia: A randomized, double-blind, sham-controlled study. *Schizophr Res* 2018; **195:** 475-480.

27. Boutros NN, Gueorguieva R, Hoffman RE, Oren DA, Feingold A, Berman RM. Lack of a therapeutic effect of a 2-week sub-threshold transcranial magnetic stimulation course for treatment-resistant depression. *Psychiatry Res* 2002; **113**(3)**:** 245-254.

28. Brunelin J, Mondino M, Gassab L, Haesebaert F, Gaha L, Suaud-Chagny MF *et al.* Examining transcranial direct-current stimulation (tDCS) as a treatment for hallucinations in schizophrenia. *Am J Psychiatry* 2012; **169**(7)**:** 719-724.

29. Brunelin J, Poulet E, Bediou B, Kallel L, Dalery J, D'Amato T *et al.* Low frequency repetitive transcranial magnetic stimulation improves source monitoring deficit in hallucinating patients with schizophrenia. *Schizophr Res* 2006; **81**(1)**:** 41-45.

30. Bulteau S, Beynel L, Marendaz C, Dall'Igna G, Peré M, Harquel S *et al.* Twice-daily neuronavigated intermittent theta burst stimulation for bipolar depression: A Randomized Sham-Controlled Pilot Study. *Neurophysiol Clin* 2019; **49**(5)**:** 371-375.

31. Carmi L, Tendler A, Bystritsky A, Hollander E, Blumberger DM, Daskalakis J *et al.* Efficacy and Safety of Deep Transcranial Magnetic Stimulation for Obsessive-Compulsive Disorder: A Prospective Multicenter Randomized Double-Blind Placebo-Controlled Trial. *American Journal of Psychiatry* 2019; **176**(11)**:** 931-938.

32. Carpenter LL, Aaronson ST, Clarke GN, Holtzheimer PE, Johnson CW, McDonald WM *et al.* rTMS with a two-coil array: Safety and efficacy for treatment resistant major depressive disorder. *Brain Stimul* 2017; **10**(5)**:** 926-933.

33. Chang C-C, Kao Y-C, Chao C-Y, Chang H-A. Enhancement of cognitive insight and higher-order neurocognitive function by fronto-temporal transcranial direct current stimulation (tDCS) in patients with schizophrenia. *Schizophrenia Research* 2019; **208:** 430-438.

34. Chang C-C, Kao Y-C, Chao C-Y, Tzeng N-S, Chang H-A. Examining bi-anodal transcranial direct current stimulation (tDCS) over bilateral dorsolateral prefrontal cortex coupled with bilateral extracephalic references as a treatment for negative symptoms in non-acute schizophrenia patients: A randomized, double-blind, sham-controlled trial. *Progress in Neuro Psychopharmacology & Biological Psychiatry* 2020; **96:** 109715.

35. Chang C-C, Tzeng N-S, Chao C-Y, Yeh C-B, Chang H-A. The effects of add-on fronto-temporal transcranial direct current stimulation (tDCS) on auditory verbal hallucinations, other psychopathological symptoms, and insight in schizophrenia: A randomized, double-blind, sham-controlled trial. *International Journal of Neuropsychopharmacology* 2018; **21**(11)**:** 979-987.

36. Chauhan P, Garg S, Tikka SK, Khattri S. Efficacy of Intensive Cerebellar Intermittent Theta Burst Stimulation (iCiTBS) in Treatment-Resistant Schizophrenia: a Randomized Placebo-Controlled Study. *Cerebellum* 2020**:** 1-8.

37. Chen SJ, Chang CH, Tsai HC, Chen ST, Lin CC. Superior antidepressant effect occurring 1 month after rTMS: Add-on rTMS for subjects with medication-resistant depression. *Neuropsychiatric Disease and Treatment* 2013; **9**(pp 397-401).

38. Chistyakov AV, Kreinin B, Marmor S, Kaplan B, Khatib A, Darawsheh N *et al.* Preliminary assessment of the therapeutic efficacy of continuous theta-burst magnetic stimulation (cTBS) in major depression: a double-blind sham-controlled study. *J Affect Disord* 2015; **170:** 225-229.

39. Chou PH, Lu MK, Tsai CH, Hsieh WT, Lai HC, Shityakov S *et al.* Antidepressant efficacy and immune effects of bilateral theta burst stimulation monotherapy in major depression: A randomized, double-blind, sham-controlled study. *Brain Behav Immun* 2020; **88:** 144-150.

40. Cohen H, Kaplan Z, Kotler M, Kouperman I, Moisa R, Grisaru N. Repetitive transcranial magnetic stimulation of the right dorsolateral prefrontal cortex in posttraumatic stress disorder: a double-blind, placebo-controlled study. *Am J Psychiatry* 2004; **161**(3)**:** 515-524.

41. Concerto C, Lanza G, Cantone M, Ferri R, Pennisi G, Bella R *et al.* Repetitive transcranial magnetic stimulation in patients with drug-resistant major depression: A six-month clinical follow-up study. *Int J Psychiatry Clin Pract* 2015; **19**(4)**:** 252-258.

42. Cordes J, Thünker J, Agelink MW, Arends M, Mobascher A, Wobrock T *et al.* Effects of 10 Hz repetitive transcranial magnetic stimulation (rTMS) on clinical global impression in chronic schizophrenia. *Psychiatry Res* 2010; **177**(1-2)**:** 32-36.

43. de Jesus DR, Gil A, Barbosa L, Lobato MI, Magalhães PV, Favalli GP *et al.* A pilot double-blind sham-controlled trial of repetitive transcranial magnetic stimulation for patients with refractory schizophrenia treated with clozapine. *Psychiatry Res* 2011; **188**(2)**:** 203-207.

44. de Lima AL, Braga FMA, da Costa RMM, Gomes EP, Brunoni AR, Pegado R. Transcranial direct current stimulation for the treatment of generalized anxiety disorder: A randomized clinical trial. *J Affect Disord* 2019; **259:** 31-37.

45. Diefenbach GJ, Bragdon LB, Zertuche L, Hyatt CJ, Hallion LS, Tolin DF *et al.* Repetitive transcranial magnetic stimulation for generalised anxiety disorder: a pilot randomised, double-blind, sham-controlled trial. *Br J Psychiatry* 2016; **209**(3)**:** 222-228.

46. Dilkov D, Hawken ER, Kaludiev E, Milev R. Repetitive transcranial magnetic stimulation of the right dorsal lateral prefrontal cortex in the treatment of generalized anxiety disorder: A randomized, double-blind sham controlled clinical trial. *Prog Neuropsychopharmacol Biol Psychiatry* 2017; **78:** 61-65.

47. Dlabac-de Lange JJ, Bais L, van Es FD, Visser BG, Reinink E, Bakker B *et al.* Efficacy of bilateral repetitive transcranial magnetic stimulation for negative symptoms of schizophrenia: results of a multicenter double-blind randomized controlled trial. *Psychol Med* 2015; **45**(6)**:** 1263-1275.

48. Dollfus S, Jaafari N, Guillin O, Trojak B, Plaze M, Saba G *et al.* High-Frequency Neuronavigated rTMS in Auditory Verbal Hallucinations: A Pilot Double-Blind Controlled Study in Patients With Schizophrenia. *Schizophrenia Bulletin* 2018; **44**(3)**:** 505-514.

49. Dunlop K, Sheen J, Schulze L, Fettes P, Mansouri F, Feffer K *et al.* Dorsomedial prefrontal cortex repetitive transcranial magnetic stimulation for treatment-refractory major depressive disorder: A three-arm, blinded, randomized controlled trial. *Brain Stimul* 2020; **13**(2)**:** 337-340.

50. Duprat R, Desmyter S, De Raedt R, van Heeringen K, Van den Abbeele D, Tandt H *et al.* Accelerated intermittent theta burst stimulation treatment in medication-resistant major depression: A fast road to remission? *Journal of Affective Disorders* 2016; **200:** 6-14.

51. Dutta P, Dhyani M, Garg S, Tikka SK, Khattri S, Mehta S *et al.* Efficacy of intensive orbitofrontal continuous Theta Burst Stimulation (iOFcTBS) in Obsessive Compulsive Disorder: A Randomized Placebo Controlled Study. *Psychiatry Res* 2021; **298:** 113784.

52. Elbeh KAM, Elserogy YMB, Khalifa HE, Ahmed MA, Hafez MH, Khedr EM. Repetitive transcranial magnetic stimulation in the treatment of obsessive-compulsive disorders: Double blind randomized clinical trial. *Psychiatry Res* 2016; **238:** 264-269.

53. Eschweiler GW, Wegerer C, Schlotter W, Spandl C, Stevens A, Bartels M *et al.* Left prefrontal activation predicts therapeutic effects of repetitive transcranial magnetic stimulation (rTMS) in major depression. *Psychiatry Res* 2000; **99**(3)**:** 161-172.

54. Eshel N, Keller CJ, Wu W, Jiang J, Mills-Finnerty C, Huemer J *et al.* Global connectivity and local excitability changes underlie antidepressant effects of repetitive transcranial magnetic stimulation. *Neuropsychopharmacology* 2020; **45**(6)**:** 1018-1025.

55. Fitzgerald PB. A randomized-controlled trial of bilateral rTMS for treatment-resistant depression. *Progress in Neurotherapeutics and Neuropsychopharmacology* 2008; **3**(1)**:** 211-226.

56. Fitzgerald PB, Benitez J, Castella Ad, Daskalakis ZJ, Brown TL, Kulkarni J. A Randomized, Controlled Trial of Sequential Bilateral Repetitive Transcranial Magnetic Stimulation for Treatment-Resistant Depression. *The American Journal of Psychiatry* 2006; **163**(1)**:** 88-94.

57. Fitzgerald PB, Benitez J, Daskalakis JZ, Brown TL, Marston NA, de Castella A *et al.* A double-blind sham-controlled trial of repetitive transcranial magnetic stimulation in the treatment of refractory auditory hallucinations. *J Clin Psychopharmacol* 2005; **25**(4)**:** 358-362.

58. Fitzgerald PB, Brown TL, Marston NA, Daskalakis ZJ, De Castella A, Kulkarni J. Transcranial magnetic stimulation in the treatment of depression: a double-blind, placebo-controlled trial. *Arch Gen Psychiatry* 2003; **60**(10)**:** 1002-1008.

59. Fitzgerald PB, Herring S, Hoy K, McQueen S, Segrave R, Kulkarni J *et al.* A study of the effectiveness of bilateral transcranial magnetic stimulation in the treatment of the negative symptoms of schizophrenia. *Brain Stimul* 2008; **1**(1)**:** 27-32.

60. Fitzgerald PB, Hoy KE, Elliot D, McQueen S, Wambeek LE, Daskalakis ZJ. A negative double-blind controlled trial of sequential bilateral rTMS in the treatment of bipolar depression. *J Affect Disord* 2016; **198:** 158-162.

61. Fitzgerald PB, Hoy KE, Herring SE, McQueen S, Peachey AVJ, Segrave RA *et al.* A double blind randomized trial of unilateral left and bilateral prefrontal cortex transcranial magnetic stimulation in treatment resistant major depression. *Journal of Affective Disorders* 2012; **139**(2)**:** 193-198.

62. Francis MM, Hummer TA, Vohs JL, Yung MG, Visco AC, Mehdiyoun NF *et al.* Cognitive effects of bilateral high frequency repetitive transcranial magnetic stimulation in early phase psychosis: a pilot study. *Brain Imaging Behav* 2019; **13**(3)**:** 852-861.

63. Fregni F, Boggio PS, Nitsche MA, Rigonatti SP, Pascual-Leone A. Cognitive effects of repeated sessions of transcranial direct current stimulation in patients with depression. *Depression & Anxiety* 2006; **23**(8)**:** 482-484.

64. Fröhlich F, Burrello TN, Mellin JM, Cordle AL, Lustenberger CM, Gilmore JH *et al.* Exploratory study of once-daily transcranial direct current stimulation (tDCS) as a treatment for auditory hallucinations in schizophrenia. *Eur Psychiatry* 2016; **33:** 54-60.

65. Gan J, Duan H, Chen Z, Shi Z, Gao C, Zhu X *et al.* Effectiveness and safety of high dose transcranial magnetic stimulation in schizophrenia with refractory negative symptoms: a randomized controlled study. *Zhonghua Yi Xue Za Zhi* 2015; **95**(47)**:** 3808-3812.

66. Garcia-Toro M, Mayol A, Arnillas H, Capllonch I, Ibarra O, Crespí M *et al.* Modest adjunctive benefit with transcranial magnetic stimulation in medication-resistant depression. *J Affect Disord* 2001; **64**(2-3)**:** 271-275.

67. Garcia-Toro M, Salva J, Daumal J, Andres J, Romera M, Lafau O *et al.* High (20-Hz) and low (1-Hz) frequency transcranial magnetic stimulation as adjuvant treatment in medication-resistant depression. *Psychiatry Res* 2006; **146**(1)**:** 53-57.

68. Garg S, Sinha VK, Tikka SK, Mishra P, Goyal N. The efficacy of cerebellar vermal deep high frequency (theta range) repetitive transcranial magnetic stimulation (rTMS) in schizophrenia: A randomized rater blind-sham controlled study. *Psychiatry Res* 2016; **243:** 413-420.

69. Gaudreault PO, Sharma A, Datta A, Nakamura-Palacios EM, King S, Malaker P *et al.* A double-blind sham-controlled phase 1 clinical trial of tDCS of the dorsolateral prefrontal cortex in cocaine inpatients: Craving, sleepiness, and contemplation to change. *Eur J Neurosci* 2021.

70. George MS, Lisanby SH, Avery D, McDonald WM, Durkalski V, Pavlicova M *et al.* Daily left prefrontal transcranial magnetic stimulation therapy for major depressive disorder: A sham-controlled randomized trial. *Archives of General Psychiatry* 2010; **67**(5)**:** 507-516.

71. George MS, Nahas Z, Molloy M, Speer AM, Oliver NC, Li XB *et al.* A controlled trial of daily left prefrontal cortex TMS for treating depression. *Biol Psychiatry* 2000; **48**(10)**:** 962-970.

72. George MS, Wassermann EM, Kimbrell TA, Little JT, Williams WE, Danielson AL *et al.* Mood improvement following daily left prefrontal repetitive transcranial magnetic stimulation in patients with depression: a placebo-controlled crossover trial. *Am J Psychiatry* 1997; **154**(12)**:** 1752-1756.

73. Gögler N, Willacker L, Funk J, Strube W, Langgartner S, Napiórkowski N *et al.* Single-session transcranial direct current stimulation induces enduring enhancement of visual processing speed in patients with major depression. *Eur Arch Psychiatry Clin Neurosci* 2017; **267**(7)**:** 671-686.

74. Gomes JS, Shiozawa P, Dias AM, Ducos DV, Akiba H, Trevizol AP *et al.* Left dorsolateral prefrontal cortex anodal tDCS effects on negative symptoms in schizophrenia. *Brain Stimulation* 2015; **8**(5)**:** 989-991.

75. Gomes JS, Trevizol AP, Ducos DV, Gadelha A, Ortiz BB, Fonseca AO *et al.* Effects of transcranial direct current stimulation on working memory and negative symptoms in schizophrenia: a phase II randomized sham-controlled trial. *Schizophr Res Cogn* 2018; **12:** 20-28.

76. Gomes PV, Brasil-Neto JP, Allam N, Rodrigues de Souza E. A randomized, double-blind trial of repetitive transcranial magnetic stimulation in obsessive-compulsive disorder with three-month follow-up. *J Neuropsychiatry Clin Neurosci* 2012; **24**(4)**:** 437-443.

77. Gowda SM, Narayanaswamy JC, Hazari N, Bose A, Chhabra H, Balachander S *et al.* Efficacy of pre-supplementary motor area transcranial direct current stimulation for treatment resistant obsessive compulsive disorder: A randomized, double blinded, sham controlled trial. *Brain Stimul* 2019; **12**(4)**:** 922-929.

78. Guan HY, Zhao JM, Wang KQ, Su XR, Pan YF, Guo JM *et al.* High-frequency neuronavigated rTMS effect on clinical symptoms and cognitive dysfunction: a pilot double-blind, randomized controlled study in Veterans with schizophrenia. *Transl Psychiatry* 2020; **10**(1)**:** 79.

79. Guleken MD, Akbas T, Erden SC, Akansel V, Al ZC, Ozer OA. The effect of bilateral high frequency repetitive transcranial magnetic stimulation on cognitive functions in schizophrenia. *Schizophrenia Research: Cognition* 2020; **22**(no pagination).

80. Guse B, Falkai P, Gruber O, Whalley H, Gibson L, Hasan A *et al.* The effect of long-term high frequency repetitive transcranial magnetic stimulation on working memory in schizophrenia and healthy controls--a randomized placebo-controlled, double-blind fMRI study. *Behav Brain Res* 2013; **237:** 300-307.

81. Haghighi M, Shayganfard M, Jahangard L, Ahmadpanah M, Bajoghli H, Pirdehghan A *et al.* Repetitive Transcranial Magnetic Stimulation (rTMS) improves symptoms and reduces clinical illness in patients suffering from OCD--Results from a single-blind, randomized clinical trial with sham cross-over condition. *J Psychiatr Res* 2015; **68:** 238-244.

82. Harika-Germaneau G, Rachid F, Chatard A, Lafay-Chebassier C, Solinas M, Thirioux B *et al.* Continuous theta burst stimulation over the supplementary motor area in refractory obsessive-compulsive disorder treatment: A randomized sham-controlled trial. *Brain Stimulation* 2019; **12**(6)**:** 1565-1571.

83. Hawken ER, Dilkov D, Kaludiev E, Simek S, Zhang F, Milev R. Transcranial Magnetic Stimulation of the Supplementary Motor Area in the Treatment of Obsessive-Compulsive Disorder: A Multi-Site Study. *Int J Mol Sci* 2016; **17**(3)**:** 420.

84. He ML, Gu ZT, Wang XY, Shi HP. Treatment of depression using sleep electroencephalogram modulated repetitive transcranial magnetic stimulation. *Chin Med J (Engl)* 2011; **124**(12)**:** 1779-1783.

85. Hernández-Ribas R, Deus J, Pujol J, Segalàs C, Vallejo J, Menchón JM *et al.* Identifying brain imaging correlates of clinical response to repetitive transcranial magnetic stimulation (rTMS) in major depression. *Brain Stimul* 2013; **6**(1)**:** 54-61.

86. Holi MM, Eronen M, Toivonen K, Toivonen P, Marttunen M, Naukkarinen H. Left prefrontal repetitive transcranial magnetic stimulation in schizophrenia. *Schizophr Bull* 2004; **30**(2)**:** 429-434.

87. Holla B, Biswal J, Ramesh V, Shivakumar V, Bharath RD, Benegal V *et al.* Effect of prefrontal tDCS on resting brain fMRI graph measures in Alcohol Use Disorders: A randomized, double-blind, sham-controlled study. *Progress in Neuro-Psychopharmacology & Biological Psychiatry* 2020; **102:** 9.

88. Holtzheimer IPE, Russo J, Claypoole KH, Roy-Byrne P, Avery DH. Shorter duration of depressive episode may predict response to repetitive transcranial magnetic stimulation. *Depression and Anxiety* 2004; **19**(1)**:** 24-30.

89. Hoppner J, Schulz M, Irmisch G, Mau R, Schlafke D, Richter J. Antidepressant efficacy of two different rTMS procedures. High frequency over left versus low frequency over right prefrontal cortex compared with sham stimulation. *Eur Arch Psychiatry Clin Neurosci* 2003; **253**(2)**:** 103-109.

90. Huang W, Shen F, Zhang J, Xing B. Effect of Repetitive Transcranial Magnetic Stimulation on Cigarette Smoking in Patients with Schizophrenia. *Shanghai Arch Psychiatry* 2016; **28**(6)**:** 309-317.

91. Huang Z, Li Y, Bianchi MT, Zhan S, Jiang F, Li N *et al.* Repetitive transcranial magnetic stimulation of the right parietal cortex for comorbid generalized anxiety disorder and insomnia: A randomized, double-blind, sham-controlled pilot study. *Brain Stimul* 2018; **11**(5)**:** 1103-1109.

92. Jahangard L, Haghighi M, Shyayganfard M, Ahmadpanah M, Bahmani DS, Bajoghli H *et al.* Repetitive Transcranial Magnetic Stimulation Improved Symptoms of Obsessive-Compulsive Disorder, but Also Cognitive Performance: Results from a Randomized Clinical Trial with a Cross-Over Design and Sham Condition. *Neuropsychobiology* 2016; **73**(4)**:** 224-232.

93. Januel D, Dumortier G, Verdon C-M, Stamatiadis L, Saba G, Cabaret W *et al.* A double-blind sham controlled study of right prefrontal repetitive transcranial magnetic stimulation (rTMS): Therapeutic and cognitive effect in medication free unipolar depression during 4 weeks. *Progress in Neuro Psychopharmacology & Biological Psychiatry* 2006; **30**(1)**:** 126-130.

94. Jeon D-W, Jung D-U, Kim S-J, Shim J-C, Moon J-J, Seo Y-S *et al.* Adjunct transcranial direct current stimulation improves cognitive function in patients with schizophrenia: A double-blind 12-week study. *Schizophrenia Research* 2018; **197:** 378-385.

95. Jin Y, Phillips B. A pilot study of the use of EEG-based synchronized Transcranial Magnetic Stimulation (sTMS) for treatment of Major Depression. *BMC Psychiatry* 2014; **14:** 13.

96. Kang JI, Kim C-H, Namkoong K, Lee C-i, Kim SJ. A randomized controlled study of sequentially applied repetitive transcranial magnetic stimulation in obsessive-compulsive disorder. *The Journal of Clinical Psychiatry* 2009; **70**(12)**:** 1645-1651.

97. Kang JI, Lee H, Jhung K, Kim KR, An SK, Yoon KJ *et al.* Frontostriatal Connectivity Changes in Major Depressive Disorder After Repetitive Transcranial Magnetic Stimulation: A Randomized Sham-Controlled Study. *J Clin Psychiatry* 2016; **77**(9)**:** e1137-e1143.

98. Kantrowitz JT, Sehatpour P, Avissar M, Horga G, Gwak A, Hoptman MJ *et al.* Significant improvement in treatment resistant auditory verbal hallucinations after 5 days of double-blind, randomized, sham controlled, fronto-temporal, transcranial direct current stimulation (tDCS): A replication/extension study. *Brain Stimul* 2019; **12**(4)**:** 981-991.

99. Kaster TS, Daskalakis ZJ, Noda Y, Knyahnytska Y, Downar J, Rajji TK *et al.* Efficacy, tolerability, and cognitive effects of deep transcranial magnetic stimulation for late-life depression: a prospective randomized controlled trial. *Neuropsychopharmacology* 2018; **43**(11)**:** 2231-2238.

100. Kauffmann CD, Cheema MA, Miller BE. Slow right prefrontal transcranial magnetic stimulation as a treatment for medication-resistant depression: a double-blind, placebo-controlled study. *Depression & Anxiety* 2004; **19**(1)**:** 59-62.

101. Kavanaugh BC, Aaronson ST, Clarke GN, Holtzheimer PE, Johnson CW, McDonald WM *et al.* Neurocognitive Effects of Repetitive Transcranial Magnetic Stimulation With a 2-Coil Device in Treatment-Resistant Major Depressive Disorder. *J ect* 2018; **34**(4)**:** 258-265.

102. Kimbrell TA, Little JT, Dunn RT, Frye MA, Greenberg BD, Wassermann EM *et al.* Frequency dependence of antidepressant response to left prefrontal repetitive transcranial magnetic stimulation (rTMS) as a function of baseline cerebral glucose metabolism. *Biological Psychiatry* 1999; **46**(12)**:** 1603-1613.

103. Kimura H, Kanahara N, Takase M, Yoshida T, Watanabe H, Iyo M. A randomized, sham-controlled study of high frequency rTMS for auditory hallucination in schizophrenia. *Psychiatry Res* 2016; **241:** 190-194.

104. Klauss J, Anders QS, Felippe LV, Ferreira LVB, Cruz MA, Nitsche MA *et al.* Lack of Effects of Extended Sessions of Transcranial Direct Current Stimulation (tDCS) Over Dorsolateral Prefrontal Cortex on Craving and Relapses in Crack-Cocaine Users. *Front Pharmacol* 2018; **9:** 11.

105. Klauss J, Anders QS, Felippe LV, Nitsche MA, Nakamura-Palacios EM. Multiple Sessions of Transcranial Direct Current Stimulation (tDCS) Reduced Craving and Relapses for Alcohol Use: A Randomized Placebo-Controlled Trial in Alcohol Use Disorder. *Front Pharmacol* 2018; **9:** 11.

106. Klein E, Kolsky Y, Puyerovsky M, Koren D, Chistyakov A, Feinsod M. Right prefrontal slow repetitive transcranial magnetic stimulation in schizophrenia: a double-blind sham-controlled pilot study. *Biol Psychiatry* 1999; **46**(10)**:** 1451-1454.

107. Klein E, Kreinin I, Chistyakov A, Koren D, Mecz L, Marmur S *et al.* Therapeutic efficacy of right prefrontal slow repetitive transcranial magnetic stimulation in major depression: a double-blind controlled study. *Arch Gen Psychiatry* 1999; **56**(4)**:** 315-320.

108. Koerselman F, Laman M, van Duijn H, van Duijn MAJ, Willems MAM. A 3-month, follow-up, randomized, placebo-controlled study of repetitive transcranial magnetic stimulation in depression. *J Clin Psychiatry* 2004; **65**(10)**:** 1323-1328.

109. Koops S, Blom JD, Bouachmir O, Slot MI, Neggers B, Sommer IE. Treating auditory hallucinations with transcranial direct current stimulation in a double-blind, randomized trial. *Schizophr Res* 2018; **201:** 329-336.

110. Koops S, Dellen EV, Schutte MJL, Nieuwdorp W, Neggers SFW, Sommer IEC. Theta Burst Transcranial Magnetic Stimulation for Auditory Verbal Hallucinations: Negative Findings from a Double-Blind-Randomized Trial. *Schizophrenia Bulletin* 2016; **42**(1)**:** 250-257.

111. Kreuzer PM, Schecklmann M, Lehner A, Wetter TC, Poeppl TB, Rupprecht R *et al.* The ACDC pilot trial: targeting the anterior cingulate by double cone coil rTMS for the treatment of depression. *Brain Stimul* 2015; **8**(2)**:** 240-246.

112. Kumar N, Vishnubhatla S, Wadhawan AN, Minhas S, Gupta P. A randomized, double blind, sham-controlled trial of repetitive transcranial magnetic stimulation (rTMS) in the treatment of negative symptoms in schizophrenia. *Brain Stimul* 2020; **13**(3)**:** 840-849.

113. Landeros-Weisenberger A, Mantovani A, Motlagh MG, de Alvarenga PG, Katsovich L, Leckman JF *et al.* Randomized Sham Controlled Double-blind Trial of Repetitive Transcranial Magnetic Stimulation for Adults With Severe Tourette Syndrome. *Brain Stimul* 2015; **8**(3)**:** 574-581.

114. Lee S, Jang K-I, Chae J-H. Association of the loudness dependence of auditory evoked potentials with clinical changes to repetitive transcranial magnetic stimulation in patients with depression. *Journal of Affective Disorders* 2018; **238:** 451-457.

115. Lee S, Jang KI, Yoon S, Chae JH. The Efficacy of Miniaturized Repetitive Transcranial Magnetic Stimulation in Patients with Depression. *Clin Psychopharmacol Neurosci* 2019; **17**(3)**:** 409-414.

116. Lee SH, Kim W, Chung YC, Jung KH, Bahk WM, Jun TY *et al.* A double blind study showing that two weeks of daily repetitive TMS over the left or right temporoparietal cortex reduces symptoms in patients with schizophrenia who are having treatment-refractory auditory hallucinations. *Neurosci Lett* 2005; **376**(3)**:** 177-181.

117. Leong K, Chan P, Ong L, Zwicker A, Willan S, Lam RW *et al.* A Randomized Sham-controlled Trial of 1-Hz and 10-Hz Repetitive Transcranial Magnetic Stimulation (rTMS) of the Right Dorsolateral Prefrontal Cortex in Civilian Post-traumatic Stress Disorder: Un essai randomisé contrôlé simulé de stimulation magnétique transcrânienne repetitive (SMTr) de 1 Hz et 10 Hz du cortex préfrontal dorsolatéral droit dans le trouble de stress post-traumatique chez des civils. *Can J Psychiatry* 2020; **65**(11)**:** 770-778.

118. Leuchter AF, Cook IA, Feifel D, Goethe JW, Husain M, Carpenter LL *et al.* Efficacy and Safety of Low-field Synchronized Transcranial Magnetic Stimulation (sTMS) for Treatment of Major Depression. *Brain Stimul* 2015; **8**(4)**:** 787-794.

119. Levkovitz Y, Isserles M, Padberg F, Lisanby SH, Bystritsky A, Xia G *et al.* Efficacy and safety of deep transcranial magnetic stimulation for major depression: a prospective multicenter randomized controlled trial. *World Psychiatry* 2015; **14**(1)**:** 64-73.

120. Li C-T, Chen M-H, Juan C-H, Huang H-H, Chen L-F, Hsieh J-C *et al.* Efficacy of prefrontal theta-burst stimulation in refractory depression: A randomized sham-controlled study. *Brain: A Journal of Neurology* 2014; **137**(7)**:** 2088-2098.

121. Li CT, Cheng CM, Chen MH, Juan CH, Tu PC, Bai YM *et al.* Antidepressant Efficacy of Prolonged Intermittent Theta Burst Stimulation Monotherapy for Recurrent Depression and Comparison of Methods for Coil Positioning: A Randomized, Double-Blind, Sham-Controlled Study. *Biol Psychiatry* 2020; **87**(5)**:** 443-450.

122. Liang Y, Wang L, Yuan TF. Targeting Withdrawal Symptoms in Men Addicted to Methamphetamine with Transcranial Magnetic Stimulation: A Randomized Clinical Trial. *JAMA Psychiatry* 2018; **75**(11)**:** 1199-1201.

123. Lindenmayer JP, Kulsa MKC, Sultana T, Kaur A, Yang R, Ljuri I *et al.* Transcranial direct-current stimulation in ultra-treatment-resistant schizophrenia. *Brain Stimul* 2019; **12**(1)**:** 54-61.

124. Lingeswaran A. Repetitive Transcranial Magnetic Stimulation in the Treatment of depression: A Randomized, Double-blind, Placebo-controlled Trial. *Indian J Psychol Med* 2011; **33**(1)**:** 35-44.

125. Loo C, Sachdev P, Elsayed H, McDarmont B, Mitchell P, Wilkinson M *et al.* Effects of a 2- to 4-week course of repetitive transcranial magnetic stimulation (rTMS) on neuropsychologic functioning, electroencephalogram, and auditory threshold in depressed patients. *Biological Psychiatry* 2001; **49**(7)**:** 615-623.

126. Loo CK, Alonzo A, Martin D, Mitchell PB, Galvez V, Sachdev P. Transcranial direct current stimulation for depression: 3-week, randomised, sham-controlled trial. *Br J Psychiatry* 2012; **200**(1)**:** 52-59.

127. Loo CK, Husain MM, McDonald WM, Aaronson S, O'Reardon JP, Alonzo A *et al.* International randomized-controlled trial of transcranial Direct Current Stimulation in depression. *Brain Stimulation* 2018; **11**(1)**:** 125-133.

128. Loo CK, Mitchell PB, Croker VM, Malhi GS, Wen W, Gandevia SC *et al.* Double-blind controlled investigation of bilateral prefrontal transcranial magnetic stimulation for the treatment of resistant major depression. *Psychol Med* 2003; **33**(1)**:** 33-40.

129. Loo CK, Mitchell PB, McFarquhar TF, Malhi GS, Sachdev PS. A sham-controlled trial of the efficacy and safety of twice-daily rTMS in major depression. *Psychol Med* 2007; **37**(3)**:** 341-349.

130. Loo CK, Sachdev P, Martin D, Pigot M, Alonzo A, Malhi GS *et al.* A double-blind, sham-controlled trial of transcranial direct current stimulation for the treatment of depression. *Int J Neuropsychopharmacol* 2010; **13**(1)**:** 61-69.

131. Ma X, Huang Y, Liao L, Jin Y. A randomized double-blinded sham-controlled trial of alpha electroencephalogram-guided transcranial magnetic stimulation for obsessive-compulsive disorder. *Chinese Medical Journal* 2014; **127**(4)**:** 601-606.

132. Manes F, Jorge R, Morcuende M, Yamada T, Paradiso S, Robinson RG. A controlled study of repetitive transcranial magnetic stimulation as a treatment of depression in the elderly. *International Psychogeriatrics* 2001; **13**(2)**:** 225-231.

133. Mansur CG, Myczkowki ML, Cabral SD, Sartorelli MDB, Bellini BB, Dias AM *et al.* Placebo effect after prefrontal magnetic stimulation in the treatment of resistant obsessive-compulsive disorder: a randomized controlled trial. *International Journal of Neuropsychopharmacology* 2011; **14**(10)**:** 1389-1397.

134. Mantovani A, Simpson HB, Fallon BA, Rossi S, Lisanby SH. Randomized sham-controlled trial of repetitive transcranial magnetic stimulation in treatment-resistant obsessive-compulsive disorder. *Int J Neuropsychopharmacol* 2010; **13**(2)**:** 217-227.

135. Martinez D, Urban N, Grassetti A, Chang D, Hu MC, Zangen A *et al.* Transcranial Magnetic Stimulation of Medial Prefrontal and Cingulate Cortices Reduces Cocaine Self-Administration: A Pilot Study. *Front Psychiatry* 2018; **9:** 80.

136. Martinotti G, Lupi M, Montemitro C, Miuli A, Di Natale C, Spano MC *et al.* Transcranial Direct Current Stimulation Reduces Craving in Substance Use Disorders: A Double-blind, Placebo-Controlled Study. *J ect* 2019; **35**(3)**:** 207-211.

137. Matsuda Y, Kito S, Igarashi Y, Shigeta M. Efficacy and Safety of Deep Transcranial Magnetic Stimulation in Office Workers with Treatment-Resistant Depression: A Randomized, Double-Blind, Sham-Controlled Trial. *Neuropsychobiology* 2020; **79**(3)**:** 208-213.

138. McDonald WM, Easley K, Byrd EH, Holtzheimer P, Tuohy S, Woodard JL *et al.* Combination rapid transcranial magnetic stimulation in treatment refractory depression. *Neuropsychiatr Dis Treat* 2006; **2**(1)**:** 85-94.

139. McGirr A, Vila-Rodriguez F, Cole J, Torres IJ, Arumugham SS, Keramatian K *et al.* Efficacy of Active vs Sham Intermittent Theta Burst Transcranial Magnetic Stimulation for Patients With Bipolar Depression: A Randomized Clinical Trial. *JAMA Netw Open* 2021; **4**(3)**:** e210963.

140. Meiron O, David J, Yaniv A. Left prefrontal transcranial direct-current stimulation reduces symptom-severity and acutely enhances working memory in schizophrenia. *Neurosci Lett* 2021**:** 135912.

141. Mogg A, Pluck G, Eranti SV, Landau S, Purvis R, Brown RG *et al.* A randomized controlled trial with 4-month follow-up of adjunctive repetitive transcranial magnetic stimulation of the left prefrontal cortex for depression. *Psychol Med* 2008; **38**(3)**:** 323-333.

142. Mogg A, Purvis R, Eranti S, Contell F, Taylor JP, Nicholson T *et al.* Repetitive transcranial magnetic stimulation for negative symptoms of schizophrenia: a randomized controlled pilot study. *Schizophr Res* 2007; **93**(1-3)**:** 221-228.

143. Moser DJ, Jorge RE, Manes F, Paradiso S, Benjamin ML, Robinson RG. Improved executive functioning following repetitive transcranial magnetic stimulation. *Neurology* 2002; **58**(8)**:** 1288-1290.

144. Mosimann UP, Schmitt W, Greenberg BD, Kosel M, Müri RM, Berkhoff M *et al.* Repetitive transcranial magnetic stimulation: a putative add-on treatment for major depression in elderly patients. *Psychiatry Res* 2004; **126**(2)**:** 123-133.

145. Movahed FS, Goradel JA, Pouresmali A, Mowlaie M. Effectiveness of Transcranial Direct Current Stimulation on Worry, Anxiety, and Depression in Generalized Anxiety Disorder: A Randomized, Single-Blind Pharmacotherapy and Sham-Controlled Clinical Trial. *Iran J Psychiatry Behav Sci* 2018; **12**(2)**:** 5.

146. Myczkowski ML, Fernandes A, Moreno M, Valiengo L, Lafer B, Moreno RA *et al.* Cognitive outcomes of TMS treatment in bipolar depression: Safety data from a randomized controlled trial. *J Affect Disord* 2018; **235:** 20-26.

147. Nahas Z, Kozel FA, Li X, Anderson B, George MS. Left prefrontal transcranial magnetic stimulation (TMS) treatment of depression in bipolar affective disorder: a pilot study of acute safety and efficacy. *Bipolar Disord* 2003; **5**(1)**:** 40-47.

148. Nam DH, Pae CU, Chae JH. Low-frequency, Repetitive Transcranial Magnetic Stimulation for the Treatment of Patients with Posttraumatic Stress Disorder: a Double-blind, Sham-controlled Study. *Clin Psychopharmacol Neurosci* 2013; **11**(2)**:** 96-102.

149. Naro A, Billeri L, Cannavo A, De Luca R, Portaro S, Bramanti P *et al.* Theta burst stimulation for the treatment of obsessive-compulsive disorder: a pilot study. *J Neural Transm (Vienna)* 2019; **126**(12)**:** 1667-1677.

150. Nauczyciel C, Le Jeune F, Naudet F, Douabin S, Esquevin A, Vérin M *et al.* Repetitive transcranial magnetic stimulation over the orbitofrontal cortex for obsessive-compulsive disorder: a double-blind, crossover study. *Transl Psychiatry* 2014; **4**(9)**:** e436.

151. O'Reardon JP, Solvason HB, Janicak PG, Sampson S, Isenberg KE, Nahas Z *et al.* Efficacy and safety of transcranial magnetic stimulation in the acute treatment of major depression: A multisite randomized controlled trial. *Biological Psychiatry* 2007; **62**(11)**:** 1208-1216.

152. Padberg F, Zwanzger P, Keck ME, Kathmann N, Mikhaiel P, Ella R *et al.* Repetitive transcranial magnetic stimulation (rTMS) in major depression: relation between efficacy and stimulation intensity. *Neuropsychopharmacology* 2002; **27**(4)**:** 638-645.

153. Padberg F, Zwanzger P, Thoma H, Kathmann N, Haag C, B DG *et al.* Repetitive transcranial magnetic stimulation (rTMS) in pharmacotherapy-refractory major depression: Comparative study of fast, slow and sham rTMS. *Psychiatry Research* 1999; **88**(3)**:** 163-171.

154. Paillère-Martinot M-L, Galinowski A, Ringuenet D, Gallarda T, Lefaucheur J-P, Bellivier F *et al.* Influence of prefrontal target region on the efficacy of repetitive transcranial magnetic stimulation in patients with medication-resistant depression: A [18F]-fluorodeoxyglucose PET and MRI study. *International Journal of Neuropsychopharmacology* 2010; **13**(1)**:** 45-59.

155. Paillère-Martinot ML, Galinowski A, Plaze M, Andoh J, Bartrés-Faz D, Bellivier F *et al.* Active and placebo transcranial magnetic stimulation effects on external and internal auditory hallucinations of schizophrenia. *Acta Psychiatr Scand* 2017; **135**(3)**:** 228-238.

156. Pallanti S, Bernardi S, Di Rollo A, Antonini S, Quercioli L. Unilateral low frequency versus sequential bilateral repetitive transcranial magnetic stimulation: is simpler better for treatment of resistant depression? *Neuroscience* 2010; **167**(2)**:** 323-328.

157. Palm U, Keeser D, Hasan A, Kupka MJ, Blautzik J, Sarubin N *et al.* Prefrontal Transcranial Direct Current Stimulation for Treatment of Schizophrenia With Predominant Negative Symptoms: A Double-Blind, Sham-Controlled Proof-of-Concept Study. *Schizophr Bull* 2016; **42**(5)**:** 1253-1261.

158. Paz Y, Friedwald K, Levkovitz Y, Zangen A, Alyagon U, Nitzan U *et al.* Randomised sham-controlled study of high-frequency bilateral deep transcranial magnetic stimulation (dTMS) to treat adult attention hyperactive disorder (ADHD): Negative results. *World J Biol Psychiatry* 2018; **19**(7)**:** 561-566.

159. Pelissolo A, Harika-Germaneau G, Rachid F, Gaudeau-Bosma C, Tanguy M-L, BenAdhira R *et al.* Repetitive transcranial magnetic stimulation to supplementary motor area in refractory obsessive-compulsive disorder treatment: A sham-controlled trial. *International Journal of Neuropsychopharmacology* 2016; **19**(8)**:** 1-6.

160. Philip NS, Aiken EE, Kelley ME, Burch W, Waterman L, Holtzheimer PE. Synchronized transcranial magnetic stimulation for posttraumatic stress disorder and comorbid major depression. *Brain Stimul* 2019; **12**(5)**:** 1335-1337.

161. Philip NS, Barredo J, Aiken E, Larson V, Jones RN, Shea MT *et al.* Theta-Burst Transcranial Magnetic Stimulation for Posttraumatic Stress Disorder. *Am J Psychiatry* 2019; **176**(11)**:** 939-948.

162. Prasko J, Pasková B, Záleský R, Novák T, Kopecek M, Bares M *et al.* The effect of repetitive transcranial magnetic stimulation (rTMS) on symptoms in obsessive compulsive disorder. A randomized, double blind, sham controlled study. *Neuro Endocrinol Lett* 2006; **27**(3)**:** 327-332.

163. Prasser J, Schecklmann M, Poeppl TB, Frank E, Kreuzer PM, Hajak G *et al.* Bilateral prefrontal rTMS and theta burst TMS as an add-on treatment for depression: a randomized placebo controlled trial. *World J Biol Psychiatry* 2015; **16**(1)**:** 57-65.

164. Prikryl R, Kasparek T, Skotakova S, Ustohal L, Kucerova H, Ceskova E. Treatment of negative symptoms of schizophrenia using repetitive transcranial magnetic stimulation in a double-blind, randomized controlled study. *Schizophr Res* 2007; **95**(1-3)**:** 151-157.

165. Prikryl R, Mikl M, Prikrylova Kucerova H, Ustohal L, Kasparek T, Marecek R *et al.* Does repetitive transcranial magnetic stimulation have a positive effect on working memory and neuronal activation in treatment of negative symptoms of schizophrenia? *Neuroendocrinology Letters* 2012; **33**(1)**:** 90-97.

166. Prikryl R, Ustohal L, Kucerova HP, Kasparek T, Jarkovsky J, Hublova V *et al.* Repetitive transcranial magnetic stimulation reduces cigarette consumption in schizophrenia patients. *Progress in Neuro-Psychopharmacology & Biological Psychiatry* 2014; **49:** 30-35.

167. Prikryl R, Ustohal L, Prikrylova Kucerova H, Kasparek T, Venclikova S, Vrzalova M *et al.* A detailed analysis of the effect of repetitive transcranial magnetic stimulation on negative symptoms of schizophrenia: a double-blind trial. *Schizophrenia Research* 2013; **149**(1-3)**:** 167-173.

168. Quan WX, Zhu XL, Qiao H, Zhang WF, Tan SP, Zhou DF *et al.* The effects of high-frequency repetitive transcranial magnetic stimulation (rTMS) on negative symptoms of schizophrenia and the follow-up study. *Neurosci Lett* 2015; **584:** 197-201.

169. Rabany L, Deutsch L, Levkovitz Y. Double-blind, randomized sham controlled study of deep-TMS add-on treatment for negative symptoms and cognitive deficits in schizophrenia. *J Psychopharmacol* 2014; **28**(7)**:** 686-690.

170. Ren Y-P, Zhou D-F, Cai Z-J, Huang Q, Lu L, Chen Q. Treatment with 1 Hz repetitive transcranial magnetic stimulation in auditory hallucination of schizophrenia: A double blind, randomized controlled trial. *Chinese Mental Health Journal* 2010; **24**(3)**:** 195-197.

171. Rossini D, Lucca A, Zanardi R, Magri L, Smeraldi E. Transcranial magnetic stimulation in treatment-resistant depressed patients: a double-blind, placebo-controlled trial. *Psychiatry Res* 2005; **137**(1-2)**:** 1-10.

172. Ruffini C, Locatelli M, Lucca A, Benedetti F, Insacco C, Smeraldi E. Augmentation effect of repetitive transcranial magnetic stimulation over the orbitofrontal cortex in drug-resistant obsessive-compulsive disorder patients: a controlled investigation. *Prim Care Companion J Clin Psychiatry* 2009; **11**(5)**:** 226-230.

173. Saba G, Verdon CM, Kalalou K, Rocamora JF, Dumortier G, Benadhira R *et al.* Transcranial magnetic stimulation in the treatment of schizophrenic symptoms: a double blind sham controlled study. *J Psychiatr Res* 2006; **40**(2)**:** 147-152.

174. Sachdev PS, Loo CK, Mitchell PB, McFarquhar TF, Malhi GS. Repetitive transcranial magnetic stimulation for the treatment of obsessive compulsive disorder: a double-blind controlled investigation. *Psychol Med* 2007; **37**(11)**:** 1645-1649.

175. Salehinejad MA, Ghanavai E, Rostami R, Nejati V. Cognitive control dysfunction in emotion dysregulation and psychopathology of major depression (MD): Evidence from transcranial brain stimulation of the dorsolateral prefrontal cortex (DLPFC). *Journal of Affective Disorders* 2017; **210:** 241-248.

176. Salehinejad MA, Rostami R, Ghanavati E. Transcranial direct current stimulation of dorsolateral prefrontal cortex of major depression: improving visual working memory, reducing depressive symptoms. *NeuroRegulation* 2015; **2**(1)**:** 37-37.

177. Sampaio B, Tortella G, Borrione L, Moffa AH, Machado-Vieira R, Cretaz E *et al.* Efficacy and Safety of Transcranial Direct Current Stimulation as an Add-on Treatment for Bipolar Depression A Randomized Clinical Trial. *Jama Psychiatry* 2018; **75**(2)**:** 158-166.

178. Sarkhel S, Sinha VK, Praharaj SK. Adjunctive high-frequency right prefrontal repetitive transcranial magnetic stimulation (rTMS) was not effective in obsessive-compulsive disorder but improved secondary depression. *J Anxiety Disord* 2010; **24**(5)**:** 535-539.

179. Schutter DJ, Laman DM, van Honk J, Vergouwen AC, Koerselman GF. Partial clinical response to 2 weeks of 2 Hz repetitive transcranial magnetic stimulation to the right parietal cortex in depression. *International Journal of Neuropsychopharmacology* 2009; **12**(5)**:** 643-650.

180. Seo H-J, Jung Y-E, Lim HK, Um Y-H, Lee CU, Chae J-H. Adjunctive low-frequency repetitive transcranial magnetic stimulation over the right dorsolateral prefrontal cortex in patients with treatment-resistant obsessive-compulsive disorder: A randomized controlled trial. *Clinical Psychopharmacology and Neuroscience* 2016; **14**(2)**:** 153-160.

181. Sharafi E, Taghva A, Arbabi M, Dadarkhah A, Ghaderi J. Transcranial Direct Current Stimulation for Treatment-Resistant Major Depression: A Double-Blind Randomized Sham-Controlled Trial. *Clin EEG Neurosci* 2019; **50**(6)**:** 375-382.

182. Shayganfard M, Jahangard L, Nazaribadie M, Haghighi M, Ahmadpanah M, Bahmani DS *et al.* Repetitive Transcranial Magnetic Stimulation Improved Symptoms of Obsessive-Compulsive Disorders but Not Executive Functions: Results from a Randomized Clinical Trial with Crossover Design and Sham Condition. *Neuropsychobiology* 2016; **74**(2)**:** 115-124.

183. Singh S, Kumar N, Verma R, Nehra A. The safety and efficacy of adjunctive 20-Hz repetitive transcranial magnetic stimulation for treatment of negative symptoms in patients with schizophrenia: A double-blinded, randomized, sham-controlled study. *Indian J Psychiatry* 2020; **62**(1)**:** 21-29.

184. Slotema CW, Blom JD, de Weijer AD, Diederen KM, Goekoop R, Looijestijn J *et al.* Can low-frequency repetitive transcranial magnetic stimulation really relieve medication-resistant auditory verbal hallucinations? Negative results from a large randomized controlled trial. *Biological Psychiatry* 2011; **69**(5)**:** 450-456.

185. Smith RC, Boules S, Mattiuz S, Youssef M, Tobe RH, Sershen H *et al.* Effects of transcranial direct current stimulation (tDCS) on cognition, symptoms, and smoking in schizophrenia: A randomized controlled study. *Schizophr Res* 2015; **168**(1-2)**:** 260-266.

186. Song R, Bai Y, Li X, Zhu J, Shi Y, Li K *et al.* Plasma Circular RNA DYM Related to Major Depressive Disorder and Rapid Antidepressant Effect Treated by Visual Cortical Repetitive Transcranial Magnetic Stimulation. *Journal of Affective Disorders* 2020; **274**(pp 486-493).

187. Speer AM, Wassermann EM, Benson BE, Herscovitch P, Post RM. Antidepressant efficacy of high and low frequency rTMS at 110% of motor threshold versus sham stimulation over left prefrontal cortex. *Brain Stimul* 2014; **7**(1)**:** 36-41.

188. Stern WM, Tormos JM, Press DZ, Pearlman C, Pascual-Leone A. Antidepressant effects of high and low frequency repetitive transcranial magnetic stimulation to the dorsolateral prefrontal cortex: a double-blind, randomized, placebo-controlled trial. *J Neuropsychiatry Clin Neurosci* 2007; **19**(2)**:** 179-186.

189. Su H, Zhong N, Gan H, Wang J, Han H, Chen T *et al.* High frequency repetitive transcranial magnetic stimulation of the left dorsolateral prefrontal cortex for methamphetamine use disorders: A randomised clinical trial. *Drug Alcohol Depend* 2017; **175:** 84-91.

190. Su T-P, Huang C-C, Wei IH. Add-On rTMS for Medication-Resistant Depression: A Randomized, Double-Blind, Sham-Controlled Trial in Chinese Patients. *The Journal of Clinical Psychiatry* 2005; **66**(7)**:** 930-937.

191. Tavares DF, Myczkowski ML, Alberto RL, Valiengo L, Rios RM, Gordon P *et al.* Treatment of Bipolar Depression with Deep TMS: Results from a Double-Blind, Randomized, Parallel Group, Sham-Controlled Clinical Trial. *Neuropsychopharmacology* 2017; **42**(13)**:** 2593-2601.

192. Taylor SF, Ho SS, Abagis T, Angstadt M, Maixner DF, Welsh RC *et al.* Changes in brain connectivity during a sham-controlled, transcranial magnetic stimulation trial for depression. *J Affect Disord* 2018; **232:** 143-151.

193. Theleritis C, Sakkas P, Paparrigopoulos T, Vitoratou S, Tzavara C, Bonaccorso S *et al.* Two Versus One High-Frequency Repetitive Transcranial Magnetic Stimulation Session per Day for Treatment-Resistant Depression: A Randomized Sham-Controlled Trial. *J ect* 2017; **33**(3)**:** 190-197.

194. Tortella G, Sampaio-Junior B, Moreno ML, Moffa AH, da Silva AF, Lafer B *et al.* Cognitive outcomes of the bipolar depression electrical treatment trial (BETTER): a randomized, double-blind, sham-controlled study. *Eur Arch Psychiatry Clin Neurosci* 2020.

195. Triggs WJ, Ricciuti N, Ward HE, Cheng J, Bowers D, Goodman WK *et al.* Right and left dorsolateral pre-frontal rTMS treatment of refractory depression: a randomized, sham-controlled trial. *Psychiatry Res* 2010; **178**(3)**:** 467-474.

196. Valiengo LDCL, Goerigk S, Gordon PC, Padberg F, Serpa MH, Koebe S *et al.* Efficacy and Safety of Transcranial Direct Current Stimulation for Treating Negative Symptoms in Schizophrenia: A Randomized Clinical Trial. *JAMA Psychiatry* 2020; **77**(2)**:** 121-129.

197. Valkonen-Korhonen M, Leinola H, Könönen M, Niskanen E, Purhonen M, Pakarinen M *et al.* Bifrontal active and sham rTMS in treatment-resistant unipolar major depression. *Nord J Psychiatry* 2018; **72**(8)**:** 586-592.

198. van Eijndhoven PFP, Bartholomeus J, Möbius M, de Bruijn A, Ferrari GRA, Mulders P *et al.* A randomized controlled trial of a standard 4-week protocol of repetitive transcranial magnetic stimulation in severe treatment resistant depression. *J Affect Disord* 2020; **274:** 444-449.

199. Wajdik C, Claypoole KH, Fawaz W, Holtzheimer PE, 3rd, Neumaier J, Dunner DL *et al.* No change in neuropsychological functioning after receiving repetitive transcranial magnetic stimulation treatment for major depression. *J ect* 2014; **30**(4)**:** 320-324.

200. Watts BV, Landon B, Groft A, Young-Xu Y. A sham controlled study of repetitive transcranial magnetic stimulation for posttraumatic stress disorder. *Brain Stimul* 2012; **5**(1)**:** 38-43.

201. Wobrock T, Guse B, Cordes J, Wölwer W, Winterer G, Gaebel W *et al.* Left prefrontal high-frequency repetitive transcranial magnetic stimulation for the treatment of schizophrenia with predominant negative symptoms: a sham-controlled, randomized multicenter trial. *Biol Psychiatry* 2015; **77**(11)**:** 979-988.

202. Wu SW, Maloney T, Gilbert DL, Dixon SG, Horn PS, Huddleston DA *et al.* Functional MRI-navigated repetitive transcranial magnetic stimulation over supplementary motor area in chronic tic disorders. *Brain Stimul* 2014; **7**(2)**:** 212-218.

203. Xiu MH, Guan HY, Zhao JM, Wang KQ, Pan YF, Su XR *et al.* Cognitive Enhancing Effect of High-Frequency Neuronavigated rTMS in Chronic Schizophrenia Patients With Predominant Negative Symptoms: A Double-Blind Controlled 32-Week Follow-up Study. *Schizophr Bull* 2020; **46**(5)**:** 1219-1230.

204. Yesavage JA, Fairchild JK, Mi Z, Biswas K, Davis-Karim A, Phibbs CS *et al.* Effect of Repetitive Transcranial Magnetic Stimulation on Treatment-Resistant Major Depression in US Veterans: A Randomized Clinical Trial. *JAMA Psychiatry* 2018; **75**(9)**:** 884-893.

205. Zavorotnyy M, Zöllner R, Rekate H, Dietsche P, Bopp M, Sommer J *et al.* Intermittent theta-burst stimulation moderates interaction between increment of N-Acetyl-Aspartate in anterior cingulate and improvement of unipolar depression. *Brain Stimul* 2020; **13**(4)**:** 943-952.

206. Zhang K, Fan X, Yuan J, Yin J, Su H, Hashimoto K *et al.* Impact of serotonin transporter gene on rTMS augmentation of SSRIs for obsessive compulsive disorder. *Neuropsychiatr Dis Treat* 2019; **15:** 1771-1779.

207. Zhang Z, Xie CM, Zhang M, Shi Y, Song R, Lu X *et al.* Task-related functional magnetic resonance imaging-based neuronavigation for the treatment of depression by individualized repetitive transcranial magnetic stimulation of the visual cortex. *Science China Life sciences* 2020; **10**.

208. Zhao S, Kong J, Li S, Tong Z, Yang C, Zhong H. Randomized controlled trial of four protocols of repetitive transcranial magnetic stimulation for treating the negative symptoms of schizophrenia. *Shanghai Arch Psychiatry* 2014; **26**(1)**:** 15-21.

209. Zheng HR, Zhang L, Li LJ, Liu P, Gao JL, Liu XY *et al.* High-frequency rTMS treatment increases left prefrontal myo-inositol in young patients with treatment-resistant depression. *Progress in Neuro-Psychopharmacology & Biological Psychiatry* 2010; **34**(7)**:** 1189-1195.

210. Zheng LN, Guo Q, Li H, Li CB, Wang JJ. Effects of repetitive transcranial magnetic stimulation with different paradigms on the cognitive function and psychotic symptoms of schizophrenia patients. *Beijing Da Xue Xue Bao Yi Xue Ban* 2012; **44**(5)**:** 732-736.

211. Zhuo KM, Tang YY, Song ZH, Wang YC, Wang JJ, Qian ZY *et al.* Repetitive transcranial magnetic stimulation as an adjunctive treatment for negative symptoms and cognitive impairment in patients with schizophrenia: a randomized, double-blind, sham-controlled trial. *Neuropsychiatric Disease and Treatment* 2019; **15:** 1141-1150.

**Supplementary material C.** Full list of excluded references

Combined treatment

1. Bares M, Brunovsky M, Stopkova P, Hejzlar M, Novak T. Transcranial Direct-Current Stimulation (tDCS) Versus Venlafaxine ER In The Treatment Of Depression: A Randomized, Double-Blind, Single-Center Study With Open-Label, Follow-Up. *Neuropsychiatr Dis Treat* 2019; **15:** 3003-3014.

*Combined treatment: Venlafaxine*

2. Bares M, Kopecek M, Novak T, Stopkova P, Sos P, Kozeny J *et al.* Low frequency (1-Hz), right prefrontal repetitive transcranial magnetic stimulation (rTMS) compared with venlafaxine ER in the treatment of resistant depression: a double-blind, single-centre, randomized study. *J Affect Disord* 2009; **118**(1-3)**:** 94-100.

*Combined treatment: Venlafaxine*

3. Bennabi D, Nicolier M, Monnin J, Tio G, Pazart L, Vandel P *et al.* Pilot study of feasibility of the effect of treatment with tDCS in patients suffering from treatment-resistant depression treated with escitalopram. *Clinical Neurophysiology* 2015; **126**(6)**:** 1185-1189.

*Combined treatment: Escitalopram*

4. Bimorgh MS, Omidi A, Ghoreishi FS, Ardani AR, Ghaderi A, Banafshe HR. The Effect of Transcranial Direct Current Stimulation on Relapse, Anxiety, and Depression in Patients With Opioid Dependence Under Methadone Maintenance Treatment: A Pilot Study. *Front Pharmacol* 2020; **11:** 9.

*Combined treatment: Methadone*

5. Bretlau LG, Lunde M, Lindberg L, Undén M, Dissing S, Bech P. Repetitive transcranial magnetic stimulation (rTMS) in combination with escitalopram in patients with treatment-resistant major depression: a double-blind, randomised, sham-controlled trial. *Pharmacopsychiatry* 2008; **41**(2)**:** 41-47.

*Combined treatment: Escitalopram*

6. Brunelin J, Jalenques I, Trojak B, Attal J, Szekely D, Gay A *et al.* The efficacy and safety of low frequency repetitive transcranial magnetic stimulation for treatment-resistant depression: The results from a large multicenter French RCT. *Brain Stimulation* 2014; **7**(6)**:** 855-863.

*Combined treatment: Venlafaxine*

7. Brunoni AR, Boggio PS, De Raedt R, Benseñor IM, Lotufo PA, Namur V *et al.* Cognitive control therapy and transcranial direct current stimulation for depression: a randomized, double-blinded, controlled trial. *J Affect Disord* 2014; **162:** 43-49.

*Combined treatment: Cognitive control therapy*

**Brunoni2013**

8. Brunoni AR, Junior RF, Kemp AH, Lotufo PA, Bensenor IM, Fregni F. Differential improvement in depressive symptoms for tDCS alone and combined with pharmacotherapy: an exploratory analysis from the Sertraline vs. Electrical Current Therapy for Treating Depression Clinical Study. *International Journal of Neuropsychopharmacology* 2014; **17**(1)**:** 53-61.

9. Brunoni AR, Kemp AH, Dantas EM, Goulart AC, Nunes MA, Boggio PS *et al.* Heart rate variability is a trait marker of major depressive disorder: evidence from the sertraline vs. electric current therapy to treat depression clinical study. *Int J Neuropsychopharmacol* 2013; **16**(9)**:** 1937-1949.

10. Brunoni AR, Kemp AH, Shiozawa P, Cordeiro Q, Valiengo LC, Goulart AC *et al.* Impact of 5-HTTLPR and BDNF polymorphisms on response to sertraline versus transcranial direct current stimulation: implications for the serotonergic system. *European Neuropsychopharmacology* 2013; **23**(11)**:** 1530-1540.

11. Brunoni AR, Machado-Vieira R, Sampaio-Junior B, Vieira ELM, Valiengo L, Bensenor IM *et al.* Plasma levels of soluble TNF receptors 1 and 2 after tDCS and sertraline treatment in major depression: Results from the SELECT-TDCS trial. *Journal of Affective Disorders* 2015; **185**(pp 209-213).

12. Brunoni AR, Machado-Vieira R, Zarate CA, Valiengo L, Vieira EL, Benseñor IM *et al.* Cytokines plasma levels during antidepressant treatment with sertraline and transcranial direct current stimulation (tDCS): results from a factorial, randomized, controlled trial. *Psychopharmacology (Berl)* 2014; **231**(7)**:** 1315-1323.

13. Brunoni AR, Machado-Vieira R, Zarate CA, Jr., Vieira EL, Valiengo L, Benseñor IM *et al.* Assessment of non-BDNF neurotrophins and GDNF levels after depression treatment with sertraline and transcranial direct current stimulation in a factorial, randomized, sham-controlled trial (SELECT-TDCS): an exploratory analysis. *Prog Neuropsychopharmacol Biol Psychiatry* 2015; **56:** 91-96.

14. Brunoni AR, Machado-Vieira R, Zarate CA, Jr., Vieira EL, Vanderhasselt MA, Nitsche MA *et al.* BDNF plasma levels after antidepressant treatment with sertraline and transcranial direct current stimulation: results from a factorial, randomized, sham-controlled trial. *Eur Neuropsychopharmacol* 2014; **24**(7)**:** 1144-1151.

15. Brunoni AR, Schestatsky P, Lotufo PA, Benseñor IM, Fregni F. Comparison of blinding effectiveness between sham tDCS and placebo sertraline in a 6-week major depression randomized clinical trial. *Clin Neurophysiol* 2014; **125**(2)**:** 298-305.

16. Brunoni AR, Tortella G, Bensenor IM, Lotufo PA, Carvalho AF, Fregni F. Cognitive effects of transcranial direct current stimulation in depression: Results from the SELECT-TDCS trial and insights for further clinical trials. *Journal of Affective Disorders* 2016; **202:** 46-52.

17. Brunoni AR, Valiengo L, Baccaro A, Zanão TA, de Oliveira JF, Goulart A *et al.* The sertraline vs. electrical current therapy for treating depression clinical study: results from a factorial, randomized, controlled trial. *JAMA Psychiatry* 2013; **70**(4)**:** 383-391.

*Combined treatment: Sertraline*

**Brunoni2017**

18. Brunoni AR, Carracedo A, Amigo OM, Pellicer AL, Talib L, Carvalho AF *et al.* Association of BDNF, HTR2A, TPH1, SLC6A4, and COMT polymorphisms with tDCS and escitalopram efficacy: ancillary analysis of a double-blind, placebo-controlled trial. *Braz J Psychiatry* 2020; **42**(2)**:** 128-135.

19. Brunoni AR, Moffa AH, Sampaio-Junior B, Borrione L, Moreno ML, Fernandes RA *et al.* Trial of Electrical Direct-Current Therapy versus Escitalopram for Depression. *N Engl J Med* 2017; **376**(26)**:** 2523-2533.

20. Brunoni AR, Padberg F, Vieira ELM, Teixeira AL, Carvalho AF, Lotufo PA *et al.* Plasma biomarkers in a placebo-controlled trial comparing tDCS and escitalopram efficacy in major depression. *Progress in Neuro-Psychopharmacology & Biological Psychiatry* 2018; **86:** 211-217.

21. Bulubas L, Padberg F, Bueno PV, Duran F, Busatto G, Amaro E, Jr. *et al.* Antidepressant effects of tDCS are associated with prefrontal gray matter volumes at baseline: Evidence from the ELECT-TDCS trial. *Brain Stimul* 2019; **12**(5)**:** 1197-1204.

22. Bulubas L, Padberg F, Mezger E, Suen P, Bueno PV, Duran F *et al.* Prefrontal resting-state connectivity and antidepressant response: no associations in the ELECT-TDCS trial. *Eur Arch Psychiatry Clin Neurosci* 2020.

23. Goerigk SA, Padberg F, Bühner M, Sarubin N, Kaster TS, Daskalakis ZJ *et al.* Distinct trajectories of response to prefrontal tDCS in major depression: results from a 3-arm randomized controlled trial. *Neuropsychopharmacology* 2021; **46**(4)**:** 774-782.

24. Moreno ML, Goerigk SA, Bertola L, Suemoto CK, Razza LB, Moffa AH *et al.* Cognitive changes after tDCS and escitalopram treatment in major depressive disorder: Results from the placebo-controlled ELECT-TDCS trial. *Journal of Affective Disorders* 2020; **263**(pp 344-352).

*Combined treatment: Escitalopram*

25. Buchholtz PE, Ashkanian M, Hjerrild S, Hauptmann LK, Devantier TA, Jensen P *et al.* Low-frequency rTMS inhibits the anti-depressive effect of ECT. A pilot study. *Acta Neuropsychiatr* 2020**:** 1-11.

*Combined treatment: ECT*

26. Buday J, Albrecht J, Podgorná G, Mareš T, Le TH, Čapek V *et al.* Seizure threshold manipulation in electroconvulsive therapy via repetitive transcranial magnetic stimulation. A novel way of augmentation? *Brain Stimul* 2020; **13**(6)**:** 1631-1638.

*Combined treatment: ECT*

27. Casarotto S, Canali P, Rosanova M, Pigorini A, Fecchio M, Mariotti M *et al.* Assessing the Effects of Electroconvulsive Therapy on Cortical Excitability by Means of Transcranial Magnetic Stimulation and Electroencephalography. *Brain Topogr* 2013; **26**(2)**:** 326-337.

*Combined treatment: ECT*

28. Chen TZ, Su H, Li RH, Jiang HF, Li XT, Wu QY *et al.* The exploration of optimized protocol for repetitive transcranial magnetic stimulation in the treatment of methamphetamine use disorder: A randomized sham-controlled study. *EBioMedicine* 2020; **60:** 9.

*Combined treatment: Treatment as usual (including detoxification, psychological consultation, exercise, medical care, and anti-relapse education)*

29. Chistyakov AV, Kaplan B, Rubichek O, Kreinin I, Koren D, Feinsod M *et al.* Antidepressant effects of different schedules of repetitive transcranial magnetic stimulation vs. clomipramine in patients with major depression: relationship to changes in cortical excitability. *Int J Neuropsychopharmacol* 2005; **8**(2)**:** 223-233.

*Combined treatment: Clomipramine*

30. Cobb AR, O'Connor P, Zaizar E, Caulfield K, Gonzalez-Lima F, Telch MJ. tDCS-Augmented in vivo exposure therapy for specific fears: A randomized clinical trial. *Journal of Anxiety Disorders* 2021; **78 (no pagination)**.

*Combined treatment: Exposure therapy*

31. Dai L, Wang P, Zhang P, Guo Q, Du H, Li F *et al.* The therapeutic effect of repetitive transcranial magnetic stimulation in elderly depression patients. *Medicine (Baltimore)* 2020; **99**(32)**:** e21493.

*Combined treatment: Escitalopram*

32. Dalton B, Bartholdy S, McClelland J, Kekic M, Rennalls SJ, Werthmann J *et al.* Randomised controlled feasibility trial of real versus sham repetitive transcranial magnetic stimulation treatment in adults with severe and enduring anorexia nervosa: the TIARA study. *BMJ Open* 2018; **8**(7)**:** e021531.

*Combined treatment: Treatment as usual (Specialist ED outpatient or day-patient treatment, or no treatment)*

33. Deppermann S, Notzon S, Kroczek A, Rosenbaum D, Haeussinger FB, Diemer J *et al.* Functional co-activation within the prefrontal cortex supports the maintenance of behavioural performance in fear-relevant situations before an iTBS modulated virtual reality challenge in participants with spider phobia. *Behavioural Brain Research* 2016; **307:** 208-217.

*Combined treatment: Virtual reality exposure*

34. Deppermann S, Vennewald N, Diemer J, Sickinger S, Haeussinger FB, Dresler T *et al.* Neurobiological and clinical effects of fNIRS-controlled rTMS in patients with panic disorder/agoraphobia during cognitive-behavioural therapy. *Neuroimage Clin* 2017; **16:** 668-677.

*Combined treatment: Cognitive behavioural therapy*

35. Deppermann S, Vennewald N, Diemer J, Sickinger S, Haeussinger FB, Notzon S *et al.* Does rTMS alter neurocognitive functioning in patients with panic disorder/agoraphobia? An fNIRS-based investigation of prefrontal activation during a cognitive task and its modulation via sham-controlled rTMS. *Biomed Res Int* 2014; **2014:** 542526.

*Combined treatment: Psychotherapy*

36. Donse L, Padberg F, Sack AT, Rush AJ, Arns M. Simultaneous rTMS and psychotherapy in major depressive disorder: Clinical outcomes and predictors from a large naturalistic study. *Brain Stimulation* 2018; **11**(2)**:** 337-345.

*Combined treatment: Psychotherapy*

37. Eche J, Mondino M, Haesebaert F, Saoud M, Poulet E, Brunelin J. Low- vs High-Frequency Repetitive Transcranial Magnetic Stimulation as an Add-On Treatment for Refractory Depression. *Front Psychiatry* 2012; **3:** 13.

*Combined treatment: Venlafaxine*

38. Eichhammer P, Kharraz A, Wiegand R, Langguth B, Frick U, Aigner JM *et al.* Sleep deprivation in depression stabilizing antidepressant effects by repetitive transcranial magnetic stimulation. *Life Sci* 2002; **70**(15)**:** 1741-1749.

*Combined treatment: Partial sleep deprivation*

39. Eranti S, Mogg A, Pluck G, Landau S, Purvis R, Brown RG *et al.* A randomized, controlled trial with 6-month follow-up of repetitive transcranial magnetic stimulation and electroconvulsive therapy for severe depression. *Am J Psychiatry* 2007; **164**(1)**:** 73-81.

*Combined treatment: ECT*

40. Fregni F, Ono CR, Santos CM, Bermpohl F, Buchpiguel C, Barbosa ER *et al.* Effects of antidepressant treatment with rTMS and fluoxetine on brain perfusion in PD. *Neurology* 2006; **66**(11)**:** 1629-1637.

*Combined treatment: Fluoxetine*

41. Fryml LD, Pelic CG, Acierno R, Tuerk P, Yoder M, Borckardt JJ *et al.* Exposure Therapy and Simultaneous Repetitive Transcranial Magnetic Stimulation: A Controlled Pilot Trial for the Treatment of Posttraumatic Stress Disorder. *J ect* 2019; **35**(1)**:** 53-60.

*Combined treatment: Exposure therapy*

42. García-Toro M, Pascual-Leone A, Romera M, González A, Micó J, Ibarra O *et al.* Prefrontal repetitive transcranial magnetic stimulation as add on treatment in depression. *J Neurol Neurosurg Psychiatry* 2001; **71**(4)**:** 546-548.

*Combined treatment: Sertraline*

43. George MS, Raman R, Benedek DM, Pelic CG, Grammer GG, Stokes KT *et al.* A two-site pilot randomized 3 day trial of high dose left prefrontal repetitive transcranial magnetic stimulation (rTMS) for suicidal inpatients. *Brain Stimul* 2014; **7**(3)**:** 421-431.

*Combined treatment: Inpatient suicidality treatment*

44. Goyal N, Nizamie SH, Desarkar P. Efficacy of adjuvant high frequency repetitive transcranial magnetic stimulation on negative and positive symptoms of schizophrenia: preliminary results of a double-blind sham-controlled study. *Journal of Neuropsychiatry & Clinical Neurosciences* 2007; **19**(4)**:** 464-467.

*Combined treatment: Antipsychotic medication started at beginning of trial*

**Hausman2004**

45. Hausmann A, Kemmler G, Walpoth M, Mechtcheriakov S, Kramer-Reinstadler K, Lechner T *et al.* No benefit derived from repetitive transcranial magnetic stimulation in depression: a prospective, single centre, randomised, double blind, sham controlled "add on" trial. *J Neurol Neurosurg Psychiatry* 2004; **75**(2)**:** 320-322.

46. Hausmann A, Pascual-Leone A, Kemmler G, Rupp CI, Lechner-Schoner T, Kramer-Reinstadler K *et al.* No deterioration of cognitive performance in an aggressive unilateral and bilateral antidepressant rTMS add-on trial. *J Clin Psychiatry* 2004; **65**(6)**:** 772-782.

*Combined treatment: Antidepressant medication started on first day of stimulation*

47. Herrmann MJ, Katzorke A, Busch Y, Gromer D, Polak T, Pauli P *et al.* Medial prefrontal cortex stimulation accelerates therapy response of exposure therapy in acrophobia. *Brain Stimulation* 2017; **10**(2)**:** 291-297.

*Combined treatment: Exposure therapy*

48. Herwig U, Fallgatter AJ, Höppner J, Eschweiler GW, Kron M, Hajak G *et al.* Antidepressant effects of augmentative transcranial magnetic stimulation: randomised multicentre trial. *Br J Psychiatry* 2007; **191:** 441-448.

*Combined treatment: Co-initiated of antidepressant medication*

49. Herwig U, Lampe Y, Juengling FD, Wunderlich A, Walter H, Spitzer M *et al.* Add-on rTMS for treatment of depression: a pilot study using stereotaxic coil-navigation according to PET data. *J Psychiatr Res* 2003; **37**(4)**:** 267-275.

*Combined treatment: Six participants co-initiated antidepressant medication*

50. Hoeppner J, Padberg F, Domes G, Zinke A, Herpertz SC, Groheinrich N *et al.* Influence of repetitive transcranial magnetic stimulation on psychomotor symptoms in major depression. *European Archives of Psychiatry and Clinical Neuroscience* 2010; **260**(3)**:** 197-202.

*Combined treatment: Antidepressant medication started on the first day of stimulation*

51. Hu SH, Lai JB, Xu DR, Qi HL, Peterson BS, Bao AM *et al.* Efficacy of repetitive transcranial magnetic stimulation with quetiapine in treating bipolar II depression: a randomized, double-blinded, control study. *Sci Rep* 2016; **6:** 30537.

*Combined treatment: Quetiapine*

52. Huang M-l, Luo B-y, Hu J-b, Wang S-S, Zhou W-h, Wei N *et al.* Repetitive transcranial magnetic stimulation in combination with citalopram in young patients with first-episode major depressive disorder: A double-blind, randomized, sham-controlled trial. *Australian and New Zealand Journal of Psychiatry* 2012; **46**(3)**:** 257-264.

*Combined treatment: Citalopram*

53. Kozel FA, Motes MA, Didehbani N, DeLaRosa B, Bass C, Schraufnagel CD *et al.* Repetitive TMS to augment cognitive processing therapy in combat veterans of recent conflicts with PTSD: A randomized clinical trial. *J Affect Disord* 2018; **229:** 506-514.

*Combined treatment: Cognitive processing therapy*

54. Krstić J, Buzadžić I, Milanović SD, Ilić NV, Pajić S, Ilić TV. Low-frequency repetitive transcranial magnetic stimulation in the right prefrontal cortex combined with partial sleep deprivation in treatment-resistant depression: a randomized sham-controlled trial. *J ect* 2014; **30**(4)**:** 325-331.

*Combined treatment: Partial sleep deprivation*

55. Li C-T, Hsieh J-C, Huang H-H, Chen M-H, Juan C-H, Tu P-C *et al.* Cognition-modulated frontal activity in prediction and augmentation of antidepressant efficacy: A randomized controlled pilot study. *Cerebral Cortex* 2016; **26**(1)**:** 202-210.

*Combined treatment: Cognitive training*

56. Li X, Yuan X, Kang Y, Pang L, Liu Y, Zhu Q *et al.* A synergistic effect between family intervention and rTMS improves cognitive and negative symptoms in schizophrenia: A randomized controlled trial. *Journal of Psychiatric Research* 2020; **126:** 81-91.

*Combined treatment: Family therapy*

57. Mayur P, Howari R, Byth K, Vannitamby R. Concomitant Transcranial Direct Current Stimulation With Ultrabrief Electroconvulsive Therapy: A 2-Week Double-Blind Randomized Sham-Controlled Trial. *J ect* 2018; **34**(4)**:** 291-295.

*Combined treatment: ECT*

58. Mittrach M, Thünker J, Winterer G, Agelink MW, Regenbrecht G, Arends M *et al.* The tolerability of rTMS treatment in schizophrenia with respect to cognitive function. *Pharmacopsychiatry* 2010; **43**(3)**:** 110-117.

*Combined treatment: Co-medication of lorazepam*

59. Nienow TM, Lim KO, MacDonald AW, III. TDCS produces incremental gain when combined with working memory training in patients with schizophrenia: A proof of concept pilot study. *Schizophrenia Research* 2016; **172**(1-3)**:** 218-219.

*Combined treatment: Working memory training*

60. Nord CL, Halahakoon DC, Limbachya T, Charpentier C, Lally N, Walsh V *et al.* Neural predictors of treatment response to brain stimulation and psychological therapy in depression: a double-blind randomized controlled trial. *Neuropsychopharmacology* 2019; **44**(9)**:** 1613-1622.

*Combined treatment: Cognitive behavioural therapy*

**Orlov2017**

61. Orlov ND, O'Daly O, Tracy DK, Daniju Y, Hodsoll J, Valdearenas L *et al.* Stimulating thought: a functional MRI study of transcranial direct current stimulation in schizophrenia. *Brain* 2017; **140**(9)**:** 2490-2497.

62. Orlov ND, Tracy DK, Joyce D, Patel S, Rodzinka-Pasko J, Dolan H *et al.* Stimulating cognition in schizophrenia: A controlled pilot study of the effects of prefrontal transcranial direct current stimulation upon memory and learning. *Brain Stimul* 2017; **10**(3)**:** 560-566.

*Combined treatment: Cognitive training*

63. Pan F, Shen Z, Jiao J, Chen J, Li S, Lu J *et al.* Neuronavigation-Guided rTMS for the Treatment of Depressive Patients With Suicidal Ideation: A Double-Blind, Randomized, Sham-Controlled Trial. *Clin Pharmacol Ther* 2020; **108**(4)**:** 826-832.

*Combined treatment: Escitalopram*

64. Pathak V, Sinha VK, Praharaj SK. Efficacy of Adjunctive High Frequency Repetitive Transcranial Magnetic Stimulation of Right Prefrontal Cortex in Adolescent Mania: A Randomized Sham-Controlled Study. *Clinical Psychopharmacology and Neuroscience* 2015; **13**(3)**:** 245-249.

*Combined treatment: Participants were started on medications as decided by the treatment team*

65. Pavlova EL, Menshikova AA, Semenov RV, Bocharnikova EN, Gotovtseva GN, Druzhkova TA *et al.* Transcranial direct current stimulation of 20- and 30-minutes combined with sertraline for the treatment of depression. *Prog Neuropsychopharmacol Biol Psychiatry* 2018; **82:** 31-38.

*Combined treatment: Sertraline*

66. Pedapati E, DiFrancesco M, Wu S, Giovanetti C, Nash T, Mantovani A *et al.* Neural correlates associated with symptom provocation in pediatric obsessive compulsive disorder after a single session of sham-controlled repetitive transcranial magnetic stimulation. *Psychiatry Research* 2015; **233**(3)**:** 466-473.

*Combined treatment: OCD-related exposure*

67. Plewnia C, Pasqualetti P, Grose S, Schlipf S, Wasserka B, Zwissler B *et al.* Treatment of major depression with bilateral theta burst stimulation: a randomized controlled pilot trial. *Journal of Affective Disorders* 2014; **156:** 219-223.

*Combined treatment: In 10 patients the antidepressant medication was changed during TBS treatment*

68. Poulet E, Brunelin J, Boeuve C, Lerond J, D'Amato T, Dalery J *et al.* Repetitive transcranial magnetic stimulation does not potentiate antidepressant treatment. *European Psychiatry: the Journal of the Association of European Psychiatrists* 2004; **19**(6)**:** 382-383.

*Combined treatment: Paroxetine*

69. Prasko J, Záleský R, Bares M, Horácek J, Kopecek M, Novák T *et al.* The effect of repetitive transcranial magnetic stimulation (rTMS) add on serotonin reuptake inhibitors in patients with panic disorder: a randomized, double blind sham controlled study. *Neuro Endocrinol Lett* 2007; **28**(1)**:** 33-38.

*Combined treatment: SRI therapy*

70. Qin BY, Dai LL, Zheng Y. [Efficacy of repetitive transcranial magnetic stimulation for alleviating clinical symptoms and suicidal ideation in elderly depressive patients: a randomized controlled trial]. *Nan Fang Yi Ke Da Xue Xue Bao* 2017; **37**(1)**:** 97-101.

*Combined treatment: Escitalopram*

71. Ray S, Nizamie SH, Akhtar S, Praharaj SK, Mishra BR, Zia-ul-Haq M. Efficacy of adjunctive high frequency repetitive transcranial magnetic stimulation of left prefrontal cortex in depression: a randomized sham controlled study. *J Affect Disord* 2011; **128**(1-2)**:** 153-159.

*Combined treatment: Participants were started on medications as decided by the treatment team*

72. Rumi DO, Gattaz WF, Rigonatti SP, Rosa MA, Fregni F, Rosa MO *et al.* Transcranial magnetic stimulation accelerates the antidepressant effect of amitriptyline in severe depression: a double-blind placebo-controlled study. *Biol Psychiatry* 2005; **57**(2)**:** 162-166.

*Combined treatment: Amitriptyline*

73. Segrave RA, Arnold S, Hoy K, Fitzgerald PB. Concurrent cognitive control training augments the antidepressant efficacy of tDCS: a pilot study. *Brain Stimul* 2014; **7**(2)**:** 325-331.

*Combined treatment: Cognitive training*

74. Shiozawa P, Gomes JS, Ducos DV, Akiba HT, Dias Á M, Trevizol AP *et al.* Effect of transcranial direct current stimulation (tDCS) over the prefrontal cortex combined with cognitive training for treating schizophrenia: a sham-controlled randomized clinical trial. *Trends Psychiatry Psychother* 2016; **38**(3)**:** 175-177.

*Combined treatment: Cognitive training*

75. Smits FM, Geuze E, Schutter D, van Honk J, Gladwin TE. Effects of tDCS during inhibitory control training on performance and PTSD, aggression and anxiety symptoms: a randomized-controlled trial in a military sample. *Psychol Med* 2021**:** 1-11.

*Combined treatment: Cognitive training*

76. Su H, Chen TZ, Jiang HF, Zhong N, Du J, Xiao K *et al.* Intermittent theta burst transcranial magnetic stimulation for methamphetamine addiction: A randomized clinical trial. *European Neuropsychopharmacology* 2020; **31:** 158-161.

*Combined treatment: Residential treatment including social skills training*

77. Tendler A, Gersner R, Roth Y, Zangen A. Alternate day dTMS combined with SSRIs for chronic treatment resistant depression: A prospective multicenter study. *Journal of Affective Disorders* 2018; **240**(pp 130-136).

*Combined treatment: SSRIS*

78. Tikka SK, Haque Nizamie S, Venkatesh Babu GM, Aggarwal N, Das AK, Goyal N. Safety and efficacy of adjunctive T burst repetitive transcranial magnetic stimulation to right inferior parietal lobule in schizophrenia patients with first-rank symptoms a pilot, exploratory study. *Journal of ECT* 2017; **33**(1)**:** 43-51.

*Combined treatment: Participants were started on medications as decided by the treatment team*

**Ullrich2012**

79. Ullrich H, Kranaster L, Sigges E, Andrich J, Sartorius A. Ultra-high-frequency left prefrontal transcranial magnetic stimulation as augmentation in severely ill patients with depression: a naturalistic sham-controlled, double-blind, randomized trial. *Neuropsychobiology* 2012; **66**(3)**:** 141-148.

80. Ullrich H, Kranaster L, Sigges E, Andrich J, Sartorius A. Neuron specific enolase and serum remain unaffected by ultra high frequency left prefrontal transcranial magnetic stimulation in patients with depression: A preliminary study. *Journal of Neural Transmission* 2012; **120**(12)**:** 1733-1736.

*Combined treatment: Venlafaxine or mirtazapine and additional standard inpatient treatment as usual*

81. Vanderhasselt M-A, De Raedt R, Namur V, Lotufo PA, Bensenor IM, Boggio PS *et al.* Transcranial electric stimulation and neurocognitive training in clinically depressed patients: A pilot study of the effects on rumination. *Progress in Neuro Psychopharmacology & Biological Psychiatry* 2015; **57:** 93-99.

*Combined treatment: Cognitive training*

82. Wang Y-M, Li N, Yang L-L, Song M, Shi L, Chen W-H *et al.* Randomized controlled trial of repetitive transcranial magnetic stimulation combined with paroxetine for the treatment of patients with first-episode major depressive disorder. *Psychiatry Research* 2017; **254:** 18-23.

*Combined treatment: Paroxetine*

83. Welch ES, Weigand A, Hooker JE, Philip NS, Tyrka AR, Press DZ *et al.* Feasibility of Computerized Cognitive-Behavioral Therapy Combined With Bifrontal Transcranial Direct Current Stimulation for Treatment of Major Depression. *Neuromodulation* 2019; **22**(8)**:** 898-903.

*Combined treatment: Computerised cognitive behavioural therapy*

84. Wing VC, Bacher I, Wu BS, Daskalakis ZJ, George TP. High frequency repetitive transcranial magnetic stimulation reduces tobacco craving in schizophrenia. *Schizophrenia Research* 2012; **139**(1-3)**:** 264-266.

*Combined treatment: Group therapy and transdermal nicotine*

85. Xie M, Jiang W, Yang H. Efficacy and safety of the Chinese herbal medicine shuganjieyu with and without adjunctive repetitive transcranial magnetic stimulation (rTMS) for geriatric depression: a randomized controlled trial. *Shanghai Arch Psychiatry* 2015; **27**(2)**:** 103-110.

*Combined treatment: Chinese herbal medicine*

86. Zheng H-r, Li L-j, Zhang L. Treatment of rTMS on young patients with intractable depression. *Chinese Journal of Clinical Psychology* 2010; **18**(1)**:** 44-46.

*Combined treatment: Active group took routine medication, the sham group did not*

87. Zhou Q, Yu C, Yu H, Zhang Y, Liu Z, Hu Z *et al.* The effects of repeated transcranial direct current stimulation on sleep quality and depression symptoms in patients with major depression and insomnia. *Sleep Med* 2020; **70:** 17-26.

*Combined treatment: Escitalopram and zopiclone*

Crossover design without phase report

1. Allenby C, Falcone M, Bernardo L, Wileyto EP, Rostain A, Ramsay JR *et al.* Transcranial direct current brain stimulation decreases impulsivity in ADHD. *Brain Stimulation* 2018; **11**(5)**:** 974-981.

2. Bloch Y, Harel EV, Aviram S, Govezensky J, Ratzoni G, Levkovitz Y. Positive effects of repetitive transcranial magnetic stimulation on attention in ADHD Subjects: a randomized controlled pilot study. *World J Biol Psychiatry* 2010; **11**(5)**:** 755-758.

3. Brennan S, McLoughlin DM, O'Connell R, Bogue J, O'Connor S, McHugh C *et al.* Anodal transcranial direct current stimulation of the left dorsolateral prefrontal cortex enhances emotion recognition in depressed patients and controls. *J Clin Exp Neuropsychol* 2017; **39**(4)**:** 384-395.

4. Calderon-Moctezuma AR, Reyes-Lopez JV, Rodriguez-Valdes R, Barbosa-Luna M, Ricardo-Garcell J, Espino-Cortes M *et al.* Improvement in borderline personality disorder symptomatology after repetitive transcranial magnetic stimulation of the dorsomedial prefrontal cortex: preliminary results. *Braz J Psychiat* 2021; **43**(1)**:** 65-69.

5. Chae J-H, Nahas Z, Wassermann E, Li X, Sethuraman G, Gilbert D *et al.* A Pilot Safety Study of Repetitive Transcranial Magnetic Stimulation (rTMS) in Tourette's Syndrome. *Cognitive and Behavioral Neurology* 2004; **17**(2)**:** 109-117.

6. Desmyter S, Duprat R, Baeken C, Van Autreve S, Audenaert K, van Heeringen K. Accelerated Intermittent Theta Burst Stimulation for Suicide Risk in Therapy-Resistant Depressed Patients: A Randomized, Sham-Controlled Trial. *Front Hum Neurosci* 2016; **10:** 480.

7. dos Santos MD, Cavenaghi VB, Mac-Kay A, Serafim V, Venturi A, Truong DQ *et al.* Non-invasive brain stimulation and computational models in post-stroke aphasic patients: single session of transcranial magnetic stimulation and transcranial direct current stimulation. A randomized clinical trial. *Sao Paulo Med J* 2017; **135**(5)**:** 475-480.

8. Dubreuil-Vall L, Gomez-Bernal F, Villegas AC, Cirillo P, Surman C, Ruffini G *et al.* Transcranial Direct Current Stimulation to the Left Dorsolateral Prefrontal Cortex Improves Cognitive Control in Patients With Attention-Deficit/Hyperactivity Disorder: A Randomized Behavioral and Neurophysiological Study. *Biol Psychiatry Cogn Neurosci Neuroimaging* 2021; **6**(4)**:** 439-448.

9. Esse Wilson J, Trumbo MC, Wilson JK, Tesche CD. Transcranial direct current stimulation (tDCS) over right temporoparietal junction (rTPJ) for social cognition and social skills in adults with autism spectrum disorder (ASD). *J Neural Transm (Vienna)* 2018; **125**(12)**:** 1857-1866.

10. Gay A, Boutet C, Sigaud T, Kamgoue A, Sevos J, Brunelin J *et al.* A single session of repetitive transcranial magnetic stimulation of the prefrontal cortex reduces cue-induced craving in patients with gambling disorder. *Eur Psychiatry* 2017; **41:** 68-74.

11. Hoy KE, Arnold SL, Emonson MRL, Daskalakis ZJ, Fitzgerald PB. An investigation into the effects of tDCS dose on cognitive performance over time in patients with schizophrenia. *Schizophrenia Research* 2014; **155**(1-3)**:** 96-100.

12. Impey D, Baddeley A, Nelson R, Labelle A, Knott V. Effects of transcranial direct current stimulation on the auditory mismatch negativity response and working memory performance in schizophrenia: a pilot study. *J Neural Transm (Vienna)* 2017; **124**(11)**:** 1489-1501.

13. Jandl M, Steyer J, Weber M, Linden DE, Rothmeier J, Maurer K *et al.* Treating auditory hallucinations by transcranial magnetic stimulation: a randomized controlled cross-over trial. *Neuropsychobiology* 2006; **53**(2)**:** 63-69.

14. Kim E-J, Yeo S, Hwang I, Park J-I, Cui Y, Jin H-M *et al.* Bilateral repetitive transcranial magnetic stimulation for auditory hallucinations in patients with schizophrenia: A randomized controlled, cross-over study. *Clinical Psychopharmacology and Neuroscience* 2014; **12**(3)**:** 222-228.

15. Klirova M, Horacek J, Novak T, Cermak J, Spaniel F, Skrdlantova L *et al.* Individualized rTMS neuronavigated according to regional brain metabolism ((18)FGD PET) has better treatment effects on auditory hallucinations than standard positioning of rTMS: a double-blind, sham-controlled study. *European Archives of Psychiatry and Clinical Neuroscience* 2013; **263**(6)**:** 475-484.

16. Lanza G, Cantone M, Aricò D, Lanuzza B, Cosentino FII, Paci D *et al.* Clinical and electrophysiological impact of repetitive low-frequency transcranial magnetic stimulation on the sensory-motor network in patients with restless legs syndrome. *Ther Adv Neurol Disord* 2018; **11:** 1756286418759973.

17. Leyman L, De Raedt R, Vanderhasselt MA, Baeken C. Effects of repetitive transcranial magnetic stimulation of the dorsolateral prefrontal cortex on the attentional processing of emotional information in major depression: A pilot study. *Psychiatry Research* 2011; **185**(1-2)**:** 102-107.

18. Loo CK, Sainsbury K, Mitchell P, Hadzi-Pavlovic D, Sachdev PS. A sham-controlled trial of left and right temporal rTMS for the treatment of auditory hallucinations. *Psychological Medicine* 2010; **40**(4)**:** 541-546.

19. Möller AL, Hjaltason O, Ivarsson O, Stefánsson SB. The effects of repetitive transcranial magnetic stimulation on depressive symptoms and the P(300) event-related potential. *Nord J Psychiatry* 2006; **60**(4)**:** 282-285.

20. Münchau A, Bloem BR, Thilo KV, Trimble MR, Rothwell JC, Robertson MM. Repetitive transcranial magnetic stimulation for Tourette syndrome. *Neurology* 2002; **59**(11)**:** 1789-1791.

21. Ni HC, Hung J, Wu CT, Wu YY, Chang CJ, Chen RS *et al.* The Impact of Single Session Intermittent Theta-Burst Stimulation over the Dorsolateral Prefrontal Cortex and Posterior Superior Temporal Sulcus on Adults with Autism Spectrum Disorder. *Front Neurosci* 2017; **11:** 255.

22. Palm U, Schiller C, Fintescu Z, Obermeier M, Keeser D, Reisinger E *et al.* Transcranial direct current stimulation in treatment resistant depression: a randomized double-blind, placebo-controlled study. *Brain Stimul* 2012; **5**(3)**:** 242-251.

23. Papazova I, Strube W, Becker B, Henning B, Schwippel T, Fallgatter AJ *et al.* Improving working memory in schizophrenia: Effects of 1 mA and 2 mA transcranial direct current stimulation to the left DLPFC. *Schizophr Res* 2018; **202:** 203-209.

24. Pascual-Leone A, Rubio B, Pallardó F, Catalá MD. Rapid-rate transcranial magnetic stimulation of left dorsolateral prefrontal cortex in drug-resistant depression. *Lancet* 1996; **348**(9022)**:** 233-237.

25. Poulet E, Brunelin J, Bediou B, Bation R, Forgeard L, Dalery J *et al.* Slow transcranial magnetic stimulation can rapidly reduce resistant auditory hallucinations in schizophrenia. *Biological Psychiatry* 2005; **57**(2)**:** 188-191.

26. Rassovsky Y, Dunn W, Wynn J, Wu AD, Iacoboni M, Hellemann G *et al.* The effect of transcranial direct current stimulation on social cognition in schizophrenia: A preliminary study. *Schizophr Res* 2015; **165**(2-3)**:** 171-174.

27. Rassovsky Y, Dunn W, Wynn JK, Wu AD, Iacoboni M, Hellemann G *et al.* Single transcranial direct current stimulation in schizophrenia: Randomized, cross-over study of neurocognition, social cognition, ERPs, and side effects. *PLoS One* 2018; **13**(5)**:** e0197023.

28. Rollnik JD, Huber TJ, Mogk H, Siggelkow S, Kropp S, Dengler R *et al.* High frequency repetitive transcranial magnetic stimulation (rTMS) of the dorsolateral prefrontal cortex in schizophrenic patients. *Neuroreport* 2000; **11**(18)**:** 4013-4015.

29. Salehinejad MA, Ghayerin E, Nejati V, Yavari F, Nitsche MA. Domain-specific Involvement of the Right Posterior Parietal Cortex in Attention Network and Attentional Control of ADHD: A Randomized, Cross-over, Sham-controlled tDCS Study. *Neuroscience* 2020; **444:** 149-159.

30. Sauvaget A, Bulteau S, Guilleux A, Leboucher J, Pichot A, Valriviere P *et al.* Both active and sham low-frequency rTMS single sessions over the right DLPFC decrease cue-induced cravings among pathological gamblers seeking treatment: A randomized, double-blind, sham-controlled crossover trial. *J Behav Addict* 2018; **7**(1)**:** 126-136.

31. Schwippel T, Papazova I, Strube W, Fallgatter AJ, Hasan A, Plewnia C. Beneficial effects of anodal transcranial direct current stimulation (tDCS) on spatial working memory in patients with schizophrenia. *European Neuropsychopharmacology* 2018; **28**(12)**:** 1339-1350.

32. Selvaraj S, Chhabra H, Dinakaran D, Sreeraj VS, Venkataram S, Narayanaswamy JC *et al.* Auditory signal detection in schizophrenia: Correlates with auditory verbal hallucinations & effect of single session transcranial direct current stimulation (tDCS). *Psychiatry Res* 2021; **297:** 113704.

33. Soff C, Sotnikova A, Christiansen H, Becker K, Siniatchkin M. Transcranial direct current stimulation improves clinical symptoms in adolescents with attention deficit hyperactivity disorder. *J Neural Transm (Vienna)* 2017; **124**(1)**:** 133-144.

34. Sotnikova A, Soff C, Tagliazucchi E, Becker K, Siniatchkin M. Transcranial Direct Current Stimulation Modulates Neuronal Networks in Attention Deficit Hyperactivity Disorder. *Brain Topogr* 2017; **30**(5)**:** 656-672.

35. Speer AM, Benson BE, Kimbrell TK, Wassermann EM, Willis MW, Herscovitch P *et al.* Opposite effects of high and low frequency rTMS on mood in depressed patients: relationship to baseline cerebral activity on PET. *J Affect Disord* 2009; **115**(3)**:** 386-394.

36. Sreeraj VS, Bose A, Chhabra H, Shivakumar V, Agarwal M, Narayanaswamy JC *et al.* Working memory performance with online-tDCS in schizophrenia: A randomized, double-blinded, sham-controlled, partial cross-over proof-of-concept study. *Asian J Psychiatr* 2020; **50:** 7.

37. Todder D, Gershi A, Perry Z, Kaplan Z, Levine J, Avirame K. Immediate Effects of Transcranial Direct Current Stimulation on Obsession-Induced Anxiety in Refractory Obsessive-Compulsive Disorder: A Pilot Study. *Journal of ECT* 2018; **34**(4)**:** e51-e57.

38. Vanderhasselt MA, de Raedt R, Baeken C, Leyman L, D'Haenen H. A single session of rTMS over the left dorsolateral prefrontal cortex influences attentional control in depressed patients. *World Journal of Biological Psychiatry* 2009; **10**(1)**:** 34-42.

39. Vercammen A, Rushby JA, Loo C, Short B, Weickert CS, Weickert TW. Transcranial direct current stimulation influences probabilistic association learning in schizophrenia. *Schizophrenia Research* 2011; **131**(1-3)**:** 198-205.

40. Wolkenstein L, Plewnia C. Amelioration of cognitive control in depression by transcranial direct current stimulation. *Biol Psychiatry* 2013; **73**(7)**:** 646-651.

41. Wolkenstein L, Rombold-Bruehl F, Bingmann T, Sommer A, Kanske P, Plewnia C. Challenging control over emotions in borderline personality disorder - a tDCS study. *Neuropsychologia* 2021; **156:** 107850.

42. Wu LL, Potenza MN, Zhou N, Kober H, Shi XH, Yip SW *et al.* A role for the right dorsolateral prefrontal cortex in enhancing regulation of both craving and negative emotions in internet gaming disorder: A randomized trial. *European Neuropsychopharmacology* 2020; **36:** 29-37.

Different stimulation technique

1. Ahn S, Mellin JM, Alagapan S, Alexander ML, Gilmore JH, Jarskog LF *et al.* Targeting reduced neural oscillations in patients with schizophrenia by transcranial alternating current stimulation. *Neuroimage* 2019; **186:** 126-136.

*Different stimulation technique: Transcranial alternating current stimulation*

2. Alexander ML, Alagapan S, Lugo CE, Mellin JM, Lustenberger C, Rubinow DR *et al.* Double-blind, randomized pilot clinical trial targeting alpha oscillations with transcranial alternating current stimulation (tACS) for the treatment of major depressive disorder (MDD). *Transl Psychiatry Psychiatry* 2019; **9**(1)**:** 106.

*Different stimulation technique: Transcranial alternating current stimulation*

3. Berger I, Dakwar-Kawar O, Grossman ES, Nahum M, Cohen Kadosh R. Scaffolding the attention-deficit/hyperactivity disorder brain using transcranial direct current and random noise stimulation: A randomized controlled trial. *Clin Neurophysiol* 2021; **132**(3)**:** 699-707.

*Different stimulation technique: Transcranial random noise stimulation*

4. Martiny K, Lunde M, Bech P. Transcranial Low Voltage Pulsed Electromagnetic Fields in Patients with Treatment-Resistant Depression. *Biological Psychiatry* 2010; **68**(2)**:** 163-169.

*Different stimulation technique: Transcranial low voltage pulsed electromagnetic fields*

5. Mellin JM, Alagapan S, Lustenberger C, Lugo CE, Alexander ML, Gilmore JH *et al.* Randomized trial of transcranial alternating current stimulation for treatment of auditory hallucinations in schizophrenia. *Eur Psychiatry* 2018; **51:** 25-33.

*Different stimulation technique: Transcranial alternating current stimulation*

6. Munz MT, Prehn-Kristensen A, Thielking F, Mölle M, Göder R, Baving L. Slow oscillating transcranial direct current stimulation during non-rapid eye movement sleep improves behavioral inhibition in attention-deficit/hyperactivity disorder. *Front Cell Neurosci* 2015; **9:** 307.

*Different stimulation technique: Slow oscillating transcranial direct current stimulation*

7. Nikolin S, Alonzo A, Martin D, Galvez V, Buten S, Taylor R *et al.* Transcranial Random Noise Stimulation for the Acute Treatment of Depression: A Randomized Controlled Trial. *International Journal of Neuropsychopharmacology* 2020; **23**(3)**:** 146-156.

*Different stimulation technique: Transcranial random noise stimulation*

8. Palm U, Hasan A, Keeser D, Falkai P, Padberg F. Transcranial random noise stimulation for the treatment of negative symptoms in schizophrenia. *Schizophrenia Research* 2013; **146**(1-3)**:** 372-373.

*Different stimulation technique: Transcranial random noise stimulation*

9. Shiozawa P, da Silva ME, Netto GT, Taiar I, Cordeiro Q. Effect of a 10-day trigeminal nerve stimulation (TNS) protocol for treating major depressive disorder: a phase II, sham-controlled, randomized clinical trial. *Epilepsy Behav* 2015; **44:** 23-26.

*Different stimulation technique: Trigeminal nerve stimulation*

10. Vaziri-Bozorg SM, Ghasemi-Esfe AR, Khalilzadeh O, Sotoudeh H, Rokni-Yazdi H, Ghanaati H *et al.* Antidepressant effects of magnetic resonance imaging-based stimulation on major depressive disorder: a double-blind randomized clinical trial. *Brain Imaging Behav* 2012; **6**(1)**:** 70-76.

*Different stimulation technique: MR stimulation*

Duplicate data

1. Chang CC, Kao YC, Chao CY, Tzeng NS, Chang HA. The effects of bi-anodal tDCS over the prefrontal cortex regions with extracephalic references placement on insight levels, cardio-respiratory and autonomic functions in schizophrenia patients and exploratory biomarker analyses for treatment response. *Int J Neuropsychopharmacol* 2020.

*Duplicate data:* Chang C-C, Kao Y-C, Chao C-Y, Tzeng N-S, Chang H-A. Examining bi-anodal transcranial direct current stimulation (tDCS) over bilateral dorsolateral prefrontal cortex coupled with bilateral extracephalic references as a treatment for negative symptoms in non-acute schizophrenia patients: A randomized, double-blind, sham-controlled trial. *Progress in Neuro Psychopharmacology & Biological Psychiatry* 2020; **96:** 109715.

2. Chauhan P, Garg S, Tikka SK, Khattri S. Efficacy of Intensive Cerebellar Intermittent Theta Burst Stimulation (iCiTBS) in Treatment-Resistant Schizophrenia: a Randomized Placebo-Controlled Study. *Cerebellum* 2021; **20**(1)**:** 116-123.

*Duplicate data:* Chauhan P, Garg S, Tikka SK, Khattri S. Efficacy of Intensive Cerebellar Intermittent Theta Burst Stimulation (iCiTBS) in Treatment-Resistant Schizophrenia: a Randomized Placebo-Controlled Study. *Cerebellum* 2020**:** 1-8.

3. Cheng CM, Juan CH, Chen MH, Chang CF, Lu HJ, Su TP *et al.* Different forms of prefrontal theta burst stimulation for executive function of medication- resistant depression: Evidence from a randomized sham-controlled study. *Prog Neuropsychopharmacol Biol Psychiatry* 2016; **66:** 35-40.

*Duplicate data:* Li C-T, Chen M-H, Juan C-H, Huang H-H, Chen L-F, Hsieh J-C *et al.* Efficacy of prefrontal theta-burst stimulation in refractory depression: A randomized sham-controlled study. *Brain: A Journal of Neurology* 2014; **137**(7)**:** 2088-2098.

4. Dlabac-de Lange JJ, Liemburg EJ, Bais L, Renken RJ, Knegtering H, Aleman A. Effect of rTMS on brain activation in schizophrenia with negative symptoms: A proof-of-principle study. *Schizophr Res* 2015; **168**(1-2)**:** 475-482.

*Duplicate data:* Dlabac-de Lange JJ, Bais L, van Es FD, Visser BG, Reinink E, Bakker B *et al.* Efficacy of bilateral repetitive transcranial magnetic stimulation for negative symptoms of schizophrenia: results of a multicenter double-blind randomized controlled trial. *Psychol Med* 2015; **45**(6)**:** 1263-1275.

5. Goegler N, Papazova I, Oviedo-Salcedo T, Filipova N, Strube W, Funk J *et al.* Parameter-Based Evaluation of Attentional Impairments in Schizophrenia and Their Modulation by Prefrontal Transcranial Direct Current Stimulation. *Front Psychiatry* 2017; **8:** 13.

*Duplicate data:* Gögler N, Willacker L, Funk J, Strube W, Langgartner S, Napiórkowski N *et al.* Single-session transcranial direct current stimulation induces enduring enhancement of visual processing speed in patients with major depression. *Eur Arch Psychiatry Clin Neurosci* 2017; **267**(7)**:** 671-686.

6. Mingli H, Zhengtian G, Xinyi W, Xiaoping T. Effects of repetitive transcranial magnetic stimulation on hypothalamic-pituitary-adrenal axis of patients with depression. *Journal of Medical Colleges of PLA* 2009; **24**(6)**:** 337-345.

*Duplicate data:* He ML, Gu ZT, Wang XY, Shi HP. Treatment of depression using sleep electroencephalogram modulated repetitive transcranial magnetic stimulation. *Chin Med J (Engl)* 2011; **124**(12)**:** 1779-1783.

7. Nahas Z, DeBrux C, Chandler V, Lorberbaum JP, Speer AM, Molloy MA *et al.* Lack of significant changes on magnetic resonance scans before and after 2 weeks of daily left prefrontal repetitive transcranial magnetic stimulation for depression. *Journal of ECT* 2000; **16**(4)**:** 380-390.

*Duplicate data:* Nahas Z, Kozel FA, Li X, Anderson B, George MS. Left prefrontal transcranial magnetic stimulation (TMS) treatment of depression in bipolar affective disorder: a pilot study of acute safety and efficacy. *Bipolar Disord* 2003; **5**(1)**:** 40-47.

8. Nahas Z, Teneback CC, Kozel A, Speer AM, DeBrux C, Molloy M *et al.* Brain effects of TMS delivered over prefrontal cortex in depressed adults: Role of stimulation frequency and coil-cortex distance. *Journal of Neuropsychiatry and Clinical Neurosciences* 2001; **13**(4)**:** 459-470.

*Duplicate data:* Nahas Z, Kozel FA, Li X, Anderson B, George MS. Left prefrontal transcranial magnetic stimulation (TMS) treatment of depression in bipolar affective disorder: a pilot study of acute safety and efficacy. *Bipolar Disord* 2003; **5**(1)**:** 40-47.

9. Theleritis C, Sakkas P, Paparrigopoulos T, Tzavara C, Politis A, Papageorgiou CC *et al.* *Assessing the Antidepressant Effect of Two and Three HF-rTMS Sessions per Day*. Medimond S R L: 40128 Bologna, 2015, 79-86pp.

*Duplicate data:* Theleritis C, Sakkas P, Paparrigopoulos T, Vitoratou S, Tzavara C, Bonaccorso S *et al.* Two Versus One High-Frequency Repetitive Transcranial Magnetic Stimulation Session per Day for Treatment-Resistant Depression: A Randomized Sham-Controlled Trial. *J ect* 2017; **33**(3)**:** 190-197.

Incomparable study

1. Ameis SH, Blumberger DM, Croarkin PE, Mabbott DJ, Lai MC, Desarkar P *et al.* Treatment of Executive Function Deficits in autism spectrum disorder with repetitive transcranial magnetic stimulation: A double-blind, sham-controlled, pilot trial. *Brain Stimul* 2020; **13**(3)**:** 539-547.

*Reason for exclusion: No comparable ASD study*

2. Bolloni C, Panella R, Pedetti M, Frascella AG, Gambelunghe C, Piccoli T *et al.* Bilateral Transcranial Magnetic Stimulation of the Prefrontal Cortex Reduces Cocaine Intake: A Pilot Study. *Front Psychiatry* 2016; **7:** 133.

*Reason for exclusion: No comparable SUD cocaine study*

3. Cachoeira CT, Leffa DT, Mittelstadt SD, Mendes LST, Brunoni AR, Pinto JV *et al.* Positive effects of transcranial direct current stimulation in adult patients with attention-deficit/hyperactivity disorder - A pilot randomized controlled study. *Psychiatry Res* 2017; **247:** 28-32.

*Reason for exclusion: No comparable tDCS ADHD study*

4. Cailhol L, Roussignol B, Klein R, Bousquet B, Simonetta-Moreau M, Schmitt L *et al.* Borderline personality disorder and rTMS: a pilot trial. *Psychiatry Res* 2014; **216**(1)**:** 155-157.

*Reason for exclusion: No other eligible TMS BPD studies*

5. Cosmo C, Baptista AF, de Araújo AN, do Rosário RS, Miranda JG, Montoya P *et al.* A Randomized, Double-Blind, Sham-Controlled Trial of Transcranial Direct Current Stimulation in Attention-Deficit/Hyperactivity Disorder. *PLoS One* 2015; **10**(8)**:** e0135371.

*Reason for exclusion: No comparable tDCS ADHD study*

6. Eapen V, Baker R, Walter A, Raghupathy V, Wehrman JJ, Sowman PF. The Role of Transcranial Direct Current Stimulation (tDCS) in Tourette Syndrome: A Review and Preliminary Findings. *Brain Sci* 2017; **7**(12)**:** 13.

*Reason for exclusion: No other eligible tDCS tourettes study*

7. Enticott PG, Fitzgibbon BM, Kennedy HA, Arnold SL, Elliot D, Peachey A *et al.* A double-blind, randomized trial of deep repetitive transcranial magnetic stimulation (rTMS) for autism spectrum disorder. *Brain Stimul* 2014; **7**(2)**:** 206-211.

*Reason for exclusion: No comparable ASD study*

8. Kekic M, McClelland J, Bartholdy S, Boysen E, Musiat P, Dalton B *et al.* Single-Session Transcranial Direct Current Stimulation Temporarily Improves Symptoms, Mood, and Self-Regulatory Control in Bulimia Nervosa: A Randomised Controlled Trial. *PLoS One* 2017; **12**(1)**:** e0167606.

*Reason for exclusion: No other eligible tDCS bulimia nervosa study*

9. Knyahnytska YO, Blumberger DM, Daskalakis ZJ, Zomorrodi R, Kaplan AS. Insula H-coil deep transcranial magnetic stimulation in severe and enduring anorexia nervosa (SE-AN): a pilot study. *Neuropsychiatr Dis Treat* 2019; **15:** 2247-2256.

*Reason for exclusion: No other eligible TMS anorexia nervosa study*

10. Lisoni J, Miotto P, Barlati S, Calza S, Crescini A, Deste G *et al.* Change in core symptoms of borderline personality disorder by tDCS: A pilot study. *Psychiatry Res* 2020; **291:** 113261.

*Reason for exclusion: No comparable tDCS BPD study*

11. Molavi P, Aziziaram S, Basharpoor S, Atadokht A, Nitsche MA, Salehinejad MA. Repeated transcranial direct current stimulation of dorsolateral-prefrontal cortex improves executive functions, cognitive reappraisal emotion regulation, and control over emotional processing in borderline personality disorder: A randomized, sham-controlled, parallel-group study. *J Affect Disord* 2020; **274:** 93-102.

*Reason for exclusion: No comparable tDCS BPD study*

12. Phillipou A, Kirkovski M, Castle DJ, Gurvich C, Abel LA, Miles S *et al.* High-definition transcranial direct current stimulation in anorexia nervosa: A pilot study. *Int J Eat Disord* 2019; **52**(11)**:** 1274-1280.

*Reason for exclusion: No other eligible tDCS anorexia nervosa study*

13. Praharaj SK, Ram D, Arora M. Efficacy of high frequency (rapid) suprathreshold repetitive transcranial magnetic stimulation of right prefrontal cortex in bipolar mania: A randomized sham controlled study. *Journal of Affective Disorders* 2009; **117**(3)**:** 146-150.

*Reason for exclusion: No other eligible mania study*

14. Schulze L, Grove M, Tamm S, Renneberg B, Roepke S. Effects of transcranial direct current stimulation on the cognitive control of negative stimuli in borderline personality disorder. *Sci Rep* 2019; **9**(1)**:** 332.

*Reason for exclusion: No comparable tDCS BPD study*

15. Soyata AZ, Aksu S, Woods AJ, Iscen P, Sacar KT, Karamursel S. Effect of transcranial direct current stimulation on decision making and cognitive flexibility in gambling disorder. *European Archives of Psychiatry and Clinical Neuroscience* 2019; **269**(3)**:** 275-284.

*Reason for exclusion: No other eligible gambling disorder study*

16. Van den Eynde F, Claudino AM, Mogg A, Horrell L, Stahl D, Ribeiro W *et al.* Repetitive Transcranial Magnetic Stimulation Reduces Cue-Induced Food Craving in Bulimic Disorders. *Biological Psychiatry* 2010; **67**(8)**:** 793-795.

*Reason for exclusion: No comparable bulimia nervosa study*

17. Walpoth M, Hoertnagl C, Mangweth-Matzek B, Kemmler G, Hinterhölzl J, Conca A *et al.* Repetitive transcranial magnetic stimulation in bulimia nervosa: preliminary results of a single-centre, randomised, double-blind, sham-controlled trial in female outpatients. *Psychother Psychosom* 2008; **77**(1)**:** 57-60.

*Reason for exclusion: No comparable bulimia nervosa study*

Ineligible sample

1. Aguirre I, Carretero B, Ibarra O, Kuhalainen J, Martínez J, Ferrer A *et al.* Age predicts low-frequency transcranial magnetic stimulation efficacy in major depression. *J Affect Disord* 2011; **130**(3)**:** 466-469.

*Reason for exclusion: Large age difference between groups*

2. Aparicio LVM, Rosa V, Razza LM, Sampaio-Junior B, Borrione L, Valiengo L *et al.* Transcranial direct current stimulation (tDCS) for preventing major depressive disorder relapse: Results of a 6-month follow-up. *Depression and Anxiety* 2019; **36**(3)**:** 262-268.

*Reason for exclusion: Patients in remission*

3. Badran BW, Taylor JJ, Devries W, Li X, Hanlon C, George MS. One step closer to patient-specific brain treatments: Interleaved transcranial magnetic stimulation (TMS)/fMRI to assess the fMRI BOLD response before and after high frequency repetitive TMS treatment. *Brain Stimulation* 2015; **Conference:** 1st International Brain Stimulation Conference. Singapore Singapore. Conference Publication: (var.pagings). 8 (2) (pp 408).

*Reason for exclusion: No formal diagnosis*

4. Balconi M, Ferrari C. Left DLPFC rTMS stimulation reduced the anxiety bias effect or how to restore the positive memory processing in high-anxiety subjects. *Psychiatry Research* 2013; **209**(3)**:** 554-559.

*Reason for exclusion: Sub-clinical participants*

5. Benadhira R, Thomas F, Bouaziz N, Braha S, Andrianisaina PS, Isaac C *et al.* A randomized, sham-controlled study of maintenance rTMS for treatment-resistant depression (TRD). *Psychiatry Res* 2017; **258:** 226-233.

*Reason for exclusion: Patients in remission*

6. Chrysikou EG, Wing EK, van Dam WO. Transcranial Direct Current Stimulation Over the Prefrontal Cortex in Depression Modulates Cortical Excitability in Emotion Regulation Regions as Measured by Concurrent Functional Magnetic Resonance Imaging: An Exploratory Study. *Biol Psychiatry Cogn Neurosci Neuroimaging* 2019.

*Reason for exclusion: No formal diagnosis*

7. Coussement C, Maurage P, Billieux J, Heeren A. Does Change in Attention Control Mediate the Impact of tDCS on Attentional Bias for Threat? Limited Evidence from a Double-blind Sham-controlled Experiment in an Unselected Sample. *Psychol Belg* 2019; **59**(1)**:** 16-32.

*Reason for exclusion: No formal diagnosis*

8. D'Anselmo A, Prete G, Tommasi L, Brancucci A. The Dichotic Right Ear Advantage Does not Change with Transcranial Direct Current Stimulation (tDCS). *Brain Stimulation* 2015; **8**(6)**:** 1238-1240.

*Reason for exclusion: No formal diagnosis*

9. Fileccia E, Di Stasi V, Poda R, Rizzo G, Stanzani-Maserati M, Oppi F *et al.* Effects on cognition of 20-day anodal transcranial direct current stimulation over the left dorsolateral prefrontal cortex in patients affected by mild cognitive impairment: a case-control study. *Neurol Sci* 2019; **40**(9)**:** 1865-1872.

*Reason for exclusion: No formal diagnosis*

10. Herbsman T, Avery D, Ramsey D, Holtzheimer P, Wadjik C, Hardaway F *et al.* More lateral and anterior prefrontal coil location is associated with better repetitive transcranial magnetic stimulation antidepressant response. *Biol Psychiatry* 2009; **66**(5)**:** 509-515.

*Reason for exclusion: Participant overlap with* Avery DH, Holtzheimer IPE, Fawaz W, Russo J, Neumaier J, Dunner DL *et al.* A controlled study of repetitive transcranial magnetic stimulation in medication-resistant major depression. *Biological Psychiatry* 2006; **59**(2)**:** 187-194.

11. Herrmann MJ, Beier JS, Simons B, Polak T. Transcranial Direct Current Stimulation (tDCS) of the Right Inferior Frontal Gyrus Attenuates Skin Conductance Responses to Unpredictable Threat Conditions. *Front Hum Neurosci* 2016; **10:** 352.

*Reason for exclusion: No formal diagnosis*

12. Impey D, de la Salle S, Knott V. Assessment of anodal and cathodal transcranial direct current stimulation (tDCS) on MMN-indexed auditory sensory processing. *Brain Cogn* 2016; **105:** 46-54.

*Reason for exclusion: No formal diagnosis*

13. Mantovani A, Aly M, Dagan Y, Allart A, Lisanby SH. Randomized sham controlled trial of repetitive transcranial magnetic stimulation to the dorsolateral prefrontal cortex for the treatment of panic disorder with comorbid major depression. *J Affect Disord* 2013; **144**(1-2)**:** 153-159.

*Reason for exclusion: Study required participants with comorbid panic and depression*

14. Mendes VA, de Jesus DR, Belmonte-de-Abreu P, Cachoeira CT, Lobato MIR. Effects of repetitive transcranial magnetic stimulation over supplementary motor area in patients with schizophrenia with obsessive-compulsive-symptoms: A pilot study. *Psychiatry Research* 2016; **242:** 34-38.

*Reason for exclusion: Study required participants with comorbid schizophrenia and OCD*

15. Miniussi C, Bonato C, Bignotti S, Gazzoli A, Gennarelli M, Pasqualetti P *et al.* Repetitive transcranial magnetic stimulation (rTMS) at high and low frequency: an efficacious therapy for major drug-resistant depression? *Clin Neurophysiol* 2005; **116**(5)**:** 1062-1071.

*Reason for exclusion: Depression not primary diagnosis*

16. Mondino M, Haesebaert F, Poulet E, Suaud-Chagny MF, Brunelin J. Fronto-temporal transcranial Direct Current Stimulation (tDCS) reduces source-monitoring deficits and auditory hallucinations in patients with schizophrenia. *Schizophrenia Research* 2015; **161**(2-3)**:** 515-516.

*Reason for exclusion: Participant overlap with:* Brunelin J, Mondino M, Gassab L, Haesebaert F, Gaha L, Suaud-Chagny MF et al. Examining transcranial direct-current stimulation (tDCS) as a treatment for hallucinations in schizophrenia. Am J Psychiatry 2012; **169**(7)**:** 719-724.

17. Mondino M, Jardri R, Suaud-Chagny MF, Saoud M, Poulet E, Brunelin J. Effects of Fronto-Temporal Transcranial Direct Current Stimulation on Auditory Verbal Hallucinations and Resting-State Functional Connectivity of the Left Temporo-Parietal Junction in Patients With Schizophrenia. *Schizophr Bull* 2016; **42**(2)**:** 318-326.

*Reason for exclusion: Participant overlap with* Brunelin J, Mondino M, Gassab L, Haesebaert F, Gaha L, Suaud-Chagny MF *et al.* Examining transcranial direct-current stimulation (tDCS) as a treatment for hallucinations in schizophrenia. *Am J Psychiatry* 2012; **169**(7)**:** 719-724.

18. Narushima K, McCormick LM, Yamada T, Thatcher RW, Robinson RG. Subgenual cingulate theta activity predicts treatment response of repetitive transcranial magnetic stimulation in participants with vascular depression. *J Neuropsychiatry Clin Neurosci* 2010; **22**(1)**:** 75-84.

*Reason for exclusion: Study required patients with vascular depression*

19. Oliveira JF, Zanão TA, Valiengo L, Lotufo PA, Benseñor IM, Fregni F *et al.* Acute working memory improvement after tDCS in antidepressant-free patients with major depressive disorder. *Neurosci Lett* 2013; **537:** 60-64.

*Reason for exclusion: Participant overlap with* Brunoni AR, Valiengo L, Baccaro A, Zanão TA, de Oliveira JF, Goulart A *et al.* The sertraline vs. electrical current therapy for treating depression clinical study: results from a factorial, randomized, controlled trial. *JAMA Psychiatry* 2013; **70**(4)**:** 383-391.

20. Philip NS, Dunner DL, Dowd SM, Aaronson ST, Brock DG, Carpenter LL *et al.* Can Medication Free, Treatment-Resistant, Depressed Patients Who Initially Respond to TMS Be Maintained Off Medications? A Prospective, 12-Month Multisite Randomized Pilot Study. *Brain Stimul* 2016; **9**(2)**:** 251-257.

*Reason for exclusion: Patients in remission*

21. Richieri R, Guedj E, Michel P, Loundou A, Auquier P, Lançon C *et al.* Maintenance transcranial magnetic stimulation reduces depression relapse: a propensity-adjusted analysis. *J Affect Disord* 2013; **151**(1)**:** 129-135.

*Reason for exclusion: Patients in remission*

22. Soltaninejad Z, Nejati V, Ekhtiari H. Effect of Anodal and Cathodal Transcranial Direct Current Stimulation on DLPFC on Modulation of Inhibitory Control in ADHD. *Journal of Attention Disorders* 2019; **23**(4)**:** 325-332.

*Reason for exclusion: No formal diagnosis*

Missing data/data not reported

1. Boggio PS, Rigonatti SP, Ribeiro RB, Myczkowski ML, Nitsche MA, Pascual-Leone A *et al.* A randomized, double-blind clinical trial on the efficacy of cortical direct current stimulation for the treatment of major depression. *Int J Neuropsychopharmacol* 2008; **11**(2)**:** 249-254.

*Reason for exclusion: Outcome means and SDs not reported*

2. Boggio PS, Rocha M, Oliveira MO, Fecteau S, Cohen RB, Campanhã C *et al.* Noninvasive brain stimulation with high-frequency and low-intensity repetitive transcranial magnetic stimulation treatment for posttraumatic stress disorder. *J Clin Psychiatry* 2010; **71**(8)**:** 992-999.

*Reason for exclusion: Outcome means and SDs not reported*

3. Cao P, Wang L, Cheng Q, Sun X, Kang Q, Dai L *et al.* Changes in serum miRNA-let-7 level in children with attention deficit hyperactivity disorder treated by repetitive transcranial magnetic stimulation or atomoxetine: An exploratory trial. *Psychiatry Research* 2019; **274:** 189-194.

*Reason for exclusion: Outcome means and SDs not reported*

4. Claudino AM, Van den Eynde F, Stahl D, Dew T, Andiappan M, Kalthoff J *et al.* Repetitive transcranial magnetic stimulation reduces cortisol concentrations in bulimic disorders. *Psychological Medicine* 2011; **41**(6)**:** 1329-1336.

*Reason for exclusion: Outcome means and SDs not reported*

5. da Silva RDF, Brunoni AR, Goerigk S, Batistuzzo MC, Costa DLD, Diniz JB *et al.* Efficacy and safety of transcranial direct current stimulation as an add-on treatment for obsessive-compulsive disorder: a randomized, sham-controlled trial. *Neuropsychopharmacology* 2021; **46**(5)**:** 1028-1034.

*Reason for exclusion: Outcome means and SDs not reported*

6. de Wit SJ, van der Werf YD, Mataix-Cols D, Trujillo JP, van Oppen P, Veltman DJ *et al.* Emotion regulation before and after transcranial magnetic stimulation in obsessive compulsive disorder. *Psychol Med* 2015; **45**(14)**:** 3059-3073.

*Reason for exclusion: Outcome means and SDs not reported*

7. Dolberg OT, Dannon PN, Schreiber S, Grunhaus L. Transcranial magnetic stimulation in patients with bipolar depression: A double blind, controlled study. *Bipolar Disorders* 2002; **4**(Suppl1)**:** 94-95.

*Reason for exclusion: No information regarding treatment parameters or patient characteristics*

8. Fitzgerald PB, McQueen S, Daskalakis ZJ, Hoy KE. A negative pilot study of daily bimodal transcranial direct current stimulation in schizophrenia. *Brain Stimul* 2014; **7**(6)**:** 813-816.

*Reason for exclusion: Unclear how many participants were in each study group*

9. Fregni F, Boggio PS, Nitsche MA, Marcolin MA, Rigonatti SP, Pascual-Leone A. Treatment of major depression with transcranial direct current stimulation. *Bipolar Disorders* 2006; **8**(2)**:** 203-204.

*Reason for exclusion: Outcome means and SDs not reported*

10. Gan H, Zhu J, Zhuo K, Zhang J, Tang Y, Qian Z *et al.* High frequency repetitive transcranial magnetic stimulation of dorsomedial prefrontal cortex for negative symptoms in patients with schizophrenia: A double-blind, randomized controlled trial. *Psychiatry Res* 2021; **299:** 113876.

*Reason for exclusion: Outcome means and SDs not reported*

11. Gay A, Jaussent I, Sigaud T, Billard S, Attal J, Seneque M *et al.* A Lack of Clinical Effect of High-frequency rTMS to Dorsolateral Prefrontal Cortex on Bulimic Symptoms: A Randomised, Double-blind Trial. *Eur Eat Disord Rev* 2016; **24**(6)**:** 474-481.

*Reason for exclusion: Outcome means and SDs not reported*

12. Gordon PC, Valiengo L, de Paula VJR, Galhardoni R, Ziemann U, de Andrade DC *et al.* Changes in motor cortical excitability in schizophrenia following transcranial direct current stimulation. *Progress in Neuro-Psychopharmacology & Biological Psychiatry* 2019; **90:** 43-48.

*Reason for exclusion: Outcome means and SDs not reported*

13. Hajak G, Marienhagen J, Langguth B, Werner S, Binder H, Eichhammer P. High-frequency repetitive transcranial magnetic stimulation in schizophrenia: a combined treatment and neuroimaging study. *Psychological Medicine* 2004; **34**(7)**:** 1157-1163.

*Reason for exclusion: Outcome means and SDs not reported*

14. Hansen PE, Videbech P, Clemmensen K, Sturlason R, Jensen HM, Vestergaard P. Repetitive transcranial magnetic stimulation as add-on antidepressant treatment. The applicability of the method in a clinical setting. *Nord J Psychiatry* 2004; **58**(6)**:** 455-457.

*Reason for exclusion: Outcome means and SDs not reported*

15. Hoffman RE, Hawkins KA, Gueorguieva R, Boutros NN, Rachid F, Carroll K *et al.* Transcranial magnetic stimulation of left temporoparietal cortex and medication-resistant auditory hallucinations. *Arch Gen Psychiatry* 2003; **60**(1)**:** 49-56.

*Reason for exclusion: Outcome means and SDs not reported*

16. Jakob F, Brakemeier E-L, Schommer NC, Quante A, Merkl A, Danker-Hopfe H *et al.* Ultrahigh frequency repetitive transcranial magnetic stimulation in unipolar depression. *Journal of Clinical Psychopharmacology* 2008; **28**(4)**:** 474-476.

*Reason for exclusion: Outcome means and SDs not reported*

17. Jin Y, Kemp AS, Huang YQ, Thai TM, Liu ZR, Xu WJ *et al.* Alpha EEG guided TMS in schizophrenia. *Brain Stimulation* 2012; **5**(4)**:** 560-568.

*Reason for exclusion: Outcome means and SDs not reported*

18. Kim J, Plitman E, Nakajima S, Alshehri Y, Iwata Y, Chung JK *et al.* Modulation of brain activity with transcranial direct current stimulation: Targeting regions implicated in impaired illness awareness in schizophrenia. *Eur Psychiatry* 2019; **61:** 63-71.

*Reason for exclusion: Outcome means and SDs not reported*

19. Li Z, Yin M, Lyu X-L, Zhang L-L, Du X-D, Hung GC-L. Delayed effect of repetitive transcranial magnetic stimulation (rTMS) on negative symptoms of schizophrenia: Findings from a randomized controlled trial. *Psychiatry Research* 2016; **240:** 333-335.

*Reason for exclusion: Outcome means and SDs not reported*

20. Loo C, Mitchell P, Sachdev P, McDarmont B, Parker G, Gandevia S. Double-blind controlled investigation of transcranial magnetic stimulation for the treatment of resistant major depression. *Am J Psychiatry* 1999; **156**(6)**:** 946-948.

*Reason for exclusion: Outcome means and SDs not reported*

21. Masafi S, Maddahi ME, Mujembari AK, Aval SB, Radfar M. Effectiveness of transcranial direct current stimulation on depression severity and automatic thoughts reduction in depressed women. *Razavi International Journal of Medicine* 2019; **7**(1).

*Reason for exclusion: No details regarding control condition*

22. Moreno ML, Vanderhasselt MA, Carvalho AF, Moffa AH, Lotufo PA, Benseñor IM *et al.* Effects of acute transcranial direct current stimulation in hot and cold working memory tasks in healthy and depressed subjects. *Neurosci Lett* 2015; **591:** 126-131.

*Reason for exclusion: Outcome means and SDs not reported*

23. Nadeau SE, Bowers D, Jones TL, Wu SS, Triggs WJ, Heilman KM. Cognitive effects of treatment of depression with repetitive transcranial magnetic stimulation. *Cogn Behav Neurol* 2014; **27**(2)**:** 77-87.

*Reason for exclusion: Outcome means and SDs not reported*

24. Novak T, Horacek J, Mohr P, Kopecek M, Skrdlantova L, Klirova M *et al.* The double-blind sham-controlled study of high-frequency rTMS (20 Hz) for negative symptoms in schizophrenia: negative results. *Neuroendocrinology Letters* 2006; **27**(1-2)**:** 209-213.

*Reason for exclusion: Outcome means and SDs not reported*

25. Schneider AL, Schneider TL, Stark H. Repetitive transcranial magnetic stimulation (rTMS) as an augmentation treatment for the negative symptoms of schizophrenia: a 4-week randomized placebo controlled study. *Brain Stimul* 2008; **1**(2)**:** 106-111.

*Reason for exclusion: Outcome means and SDs not reported*

26. Struckmann W, Persson J, Weigl W, Gingnell M, Boden R. Modulation of the prefrontal blood oxygenation response to intermittent theta-burst stimulation in depression: A sham-controlled study with functional near-infrared spectroscopy. *World Journal of Biological Psychiatry* 2020.

*Reason for exclusion: Outcome means and SDs not reported*

27. Szuba MP, O'Reardon JP, Rai AS, Snyder-Kastenberg J, Amsterdam JD, Gettes DR *et al.* Acute mood and thyroid stimulating hormone effects of transcranial magnetic stimulation in major depression. *Biological Psychiatry* 2001; **50**(1)**:** 22-27.

*Reason for exclusion: Full Hamilton scale not reported*

28. Vercammen A, Knegtering H, Bruggeman R, Westenbroek Hanneke M, Jenner JA, Slooff CJ *et al.* Effects of bilateral repetitive transcranial magnetic stimulation on treatment resistant auditory-verbal hallucinations in schizophrenia: A randomized controlled trial. *Schizophrenia Research* 2009; **114**(1-3)**:** 172-179.

*Reason for exclusion: Outcome means and SDs not reported*

29. Vercammen A, Knegtering H, Bruggeman R, Westenbroek HM, Jenner JA, Slooff CJ *et al.* Corrigendum to "Effects of bilateral repetitive transcranial magnetic stimulation on treatment resistant auditory-verbal hallucinations in schizophrenia: A randomized controlled trial" [Schizophr. Res. 114 (1-3) (October 2009) 172-179]. *Schizophrenia Research* 2015; **168**(1-2)**:** 599-600.

*Reason for exclusion: Outcome means and SDs not reported*

30. Voineskos AN, Blumberger DM, Schifani C, Hawco C, Dickie EW, Rajji TK *et al.* Effects of Repetitive Transcranial Magnetic Stimulation on Working Memory Performance and Brain Structure in People With Schizophrenia Spectrum Disorders: A Double-Blind, Randomized, Sham-Controlled Trial. *Biol Psychiatry Cogn Neurosci Neuroimaging* 2021; **6**(4)**:** 449-458.

*Reason for exclusion: Outcome means and SDs not reported*

No relevant outcome measure

1. Brunoni AR, Zanao TA, Ferrucci R, Priori A, Valiengo L, de Oliveira JF *et al.* Bifrontal tDCS prevents implicit learning acquisition in antidepressant-free patients with major depressive disorder. *Progress in Neuro-Psychopharmacology & Biological Psychiatry* 2013; **43:** 146-150.

*Primary outcome: Probabilistic learning*

2. Brunoni AR, Zanao TA, Vanderhasselt MA, Valiengo L, de Oliveira JF, Boggio PS *et al.* Enhancement of affective processing induced by bifrontal transcranial direct current stimulation in patients with major depression. *Neuromodulation* 2014; **17**(2)**:** 138-142.

*Primary outcome: Emotion processing*

3. Dumas R, Richieri R, Guedj E, Auquier P, Lancon C, Boyer L. Improvement of health-related quality of life in depression after transcranial magnetic stimulation in a naturalistic trial is associated with decreased perfusion in precuneus. *Health Qual Life Outcomes* 2012; **10:** 7.

*Primary outcome: Health-related quality of life*

4. Dunn W, Rassovsky Y, Wynn J, Wu AD, Iacoboni M, Hellemann G *et al.* The effect of bilateral transcranial direct current stimulation on early auditory processing in schizophrenia: a preliminary study. *Journal of Neural Transmission* 2017; **124**(9)**:** 1145-1149.

*Primary outcome: Auditory processing*

5. Kim SJ, Son SJ, Jang M, Kim BH, Hong SJ, Seo L *et al.* Rapid Symptom Improvement in Major Depressive Disorder Using Accelerated Repetitive Transcranial Magnetic Stimulation. *Clin Psychopharmacol Neurosci* 2021; **19**(1)**:** 73-83.

*Primary outcome: Korean Quick Inventory of Depressive Symptomatology*

6. Klooster DCW, Vos IN, Caeyenberghs K, Leemans A, David S, Besseling RMH *et al.* Indirect frontocingulate structural connectivity predicts clinical response to accelerated rtms in major depressive disorder. *Journal of Psychiatry and Neuroscience* 2020; **45**(4)**:** 243-252.

*Primary outcome: Structural connectivity*

7. Peng H, Zheng H, Li L, Liu J, Zhang Y, Shan B *et al.* High-frequency rTMS treatment increases white matter FA in the left middle frontal gyrus in young patients with treatment-resistant depression. *J Affect Disord* 2012; **136**(3)**:** 249-257.

*Primary outcome: White matter fractional anisotropy*

8. Speer AM, Kimbrell TA, Wasermann EM, Repella JD, Willis MW, Herscovitch P *et al.* Opposite effects of high and low frequency rTMS on regional brain activity in depressed patients. *Biological Psychiatry* 2000; **48**(12)**:** 1133-1141.

*Primary outcome: Brain region activity*

9. Speer AM, Repella JD, Figueras S, Demian NK, Kimbrell TA, Wasserman EM *et al.* Lack of adverse cognitive effects of 1 Hz and 20 Hz repetitive transcranial magnetic stimulation at 100% of motor threshold over left prefrontal cortex in depression. *J ect* 2001; **17**(4)**:** 259-263.

*Primary outcome: Episodic memory*

10. Walther S, Kunz M, Müller M, Zürcher C, Vladimirova I, Bachofner H *et al.* Single Session Transcranial Magnetic Stimulation Ameliorates Hand Gesture Deficits in Schizophrenia. *Schizophr Bull* 2020; **46**(2)**:** 286-293.

*Primary outcome: Hand gesture deficits*

No sham condition

1. Avissar M, Powell F, Ilieva I, Respino M, Gunning FM, Liston C *et al.* Functional connectivity of the left DLPFC to striatum predicts treatment response of depression to TMS. *Brain Stimulation* 2017; **10**(5)**:** 919-925.

2. Bagati D, Nizamie SH, Prakash R. Effect of augmentatory repetitive transcranial magnetic stimulation on auditory hallucinations in schizophrenia: randomized controlled study. *Aust N Z J Psychiatry* 2009; **43**(4)**:** 386-392.

3. Blumberger DM, Vila-Rodriguez F, Thorpe KE, Feffer K, Noda Y, Giacobbe P *et al.* Effectiveness of theta burst versus high-frequency repetitive transcranial magnetic stimulation in patients with depression (THREE-D): a randomised non-inferiority trial. *Lancet* 2018; **391**(10131)**:** 1683-1692.

4. Cao P, Xing J, Cao Y, Cheng Q, Sun X, Kang Q *et al.* Clinical effects of repetitive transcranial magnetic stimulation combined with atomoxetine in the treatment of attention-deficit hyperactivity disorder. *Neuropsychiatr Dis Treat* 2018; **14:** 3231-3240.

5. Carnell BL, Clarke P, Gill S, Galletly CA. How effective is repetitive transcranial magnetic stimulation for bipolar depression? [References]. *Journal of Affective Disorders* 2017; **209:** 270-272.

6. Cristancho P, Trapp NT, Siddiqi SH, Dixon D, Miller JP, Lenze EJ. Crossover to Bilateral Repetitive Transcranial Magnetic Stimulation: A Potential Strategy When Patients Are Not Responding to Unilateral Left-Sided High-Frequency Repetitive Transcranial Magnetic Stimulation. *J ect* 2019; **35**(1)**:** 3-5.

7. D'Urso G, Brunoni AR, Mazzaferro MP, Anastasia A, de Bartolomeis A, Mantovani A. Transcranial direct current stimulation for obsessive-compulsive disorder: A randomized, controlled, partial crossover trial. *Depress Anxiety* 2016; **33**(12)**:** 1132-1140.

8. Dadashi M, Yousefi Asl V, Morsali Y. Cognitive-Behavioral Therapy Versus Transcranial Direct Current Stimulation for Augmenting Selective Serotonin Reuptake Inhibitors in Obsessive-Compulsive Disorder Patients. *Basic Clin Neurosci* 2020; **11**(1)**:** 111-120.

9. Dannon PN, Dolberg OT, Schreiber S, Grunhaus L. Three and six-month outcome following courses of either ECT or rTMS in a population of severely depressed individuals--preliminary report. *Biol Psychiatry* 2002; **51**(8)**:** 687-690.

10. de Weijer AD, Sommer IE, Lotte Meijering A, Bloemendaal M, Neggers SF, Daalman K *et al.* High frequency rTMS; a more effective treatment for auditory verbal hallucinations? *Psychiatry Res* 2014; **224**(3)**:** 204-210.

11. Deng ZD, McClinctock SM, Lisanby SH. Brain network properties in depressed patients receiving seizure therapy: A graph theoretical analysis of peri-treatment resting EEG. *Annual International Conference Of The IEEE Engineering In Medicine And Biology Society* 2015; **2015:** 2203-2206.

12. Filipcic I, Filipcic IS, Milovac Z, Sucic S, Gajsak T, Ivezic E *et al.* Efficacy of repetitive transcranial magnetic stimulation using a figure-8-coil or an H1-Coil in treatment of major depressive disorder; A randomized clinical trial. *Journal of Psychiatric Research* 2019; **114:** 113-119.

13. Filipcic I, Milovac Z, Sucic S, Gajsak T, Filipcic IS, Ivezic E *et al.* Efficacy, Safety and Tolerability of Augmentative rTMS in Treatment of Major Depressive Disorder (MDD): A Prospective Cohort Study in Croatia. *Psychiatr Danub* 2017; **29**(1)**:** 31-38.

14. Filipčić I, Šimunović Filipčić I, Sučić S, Milovac Ž, Gereš N, Matić K *et al.* A pilot investigation of accelerated deep transcranial magnetic stimulation protocols in treatment-resistant depression. *Eur Arch Psychiatry Clin Neurosci* 2021; **271**(1)**:** 49-59.

15. Fitzgerald PB, Chen L, Richardson K, Daskalakis ZJ, Hoy KE. A pilot investigation of an intensive theta burst stimulation protocol for patients with treatment resistant depression. *Brain Stimulation* 2020; **13**(1)**:** 137-144.

16. Fitzgerald PB, Hoy K, Daskalakis ZJ, Kulkarni J. A randomized trial of the anti-depressant effects of low- and high-frequency transcranial magnetic stimulation in treatment-resistant depression. *Depress Anxiety* 2009; **26**(3)**:** 229-234.

17. Fitzgerald PB, Hoy K, Gunewardene R, Slack C, Ibrahim S, Bailey M *et al.* A randomized trial of unilateral and bilateral prefrontal cortex transcranial magnetic stimulation in treatment-resistant major depression. *Psychol Med* 2011; **41**(6)**:** 1187-1196.

18. Fitzgerald PB, Hoy K, McQueen S, Herring S, Segrave R, Been G *et al.* Priming stimulation enhances the effectiveness of low-frequency right prefrontal cortex transcranial magnetic stimulation in major depression. *J Clin Psychopharmacol* 2008; **28**(1)**:** 52-58.

19. Fitzgerald PB, Hoy K, McQueen S, Maller JJ, Herring S, Segrave R *et al.* A randomized trial of rTMS targeted with MRI based neuro-navigation in treatment-resistant depression. *Neuropsychopharmacology* 2009; **34**(5)**:** 1255-1262.

20. Fitzgerald PB, Hoy KE, Elliot D, McQueen S, Wambeek LE, Daskalakis ZJ. Exploring alternative rTMS strategies in non-responders to standard high frequency left-sided treatment: A switching study. *J Affect Disord* 2018; **232:** 79-82.

21. Fitzgerald PB, Hoy KE, Elliot D, Susan McQueen RN, Wambeek LE, Daskalakis ZJ. Accelerated repetitive transcranial magnetic stimulation in the treatment of depression. *Neuropsychopharmacology* 2018; **43**(7)**:** 1565-1572.

22. Fitzgerald PB, Hoy KE, Reynolds J, Singh A, Gunewardene R, Slack C *et al.* A pragmatic randomized controlled trial exploring the relationship between pulse number and response to repetitive transcranial magnetic stimulation treatment in depression. *Brain Stimulation* 2019.

23. Fitzgerald PB, Hoy KE, Reynolds J, Singh A, Gunewardene R, Slack C *et al.* A pragmatic randomized controlled trial exploring the relationship between pulse number and response to repetitive transcranial magnetic stimulation treatment in depression. *Brain Stimul* 2020; **13**(1)**:** 145-152.

24. Fitzgerald PB, Hoy KE, Singh A, Gunewardene R, Slack C, Ibrahim S *et al.* Equivalent beneficial effects of unilateral and bilateral prefrontal cortex transcranial magnetic stimulation in a large randomized trial in treatment-resistant major depression. *Int J Neuropsychopharmacol* 2013; **16**(9)**:** 1975-1984.

25. Fitzgerald PB, Huntsman S, Gunewardene R, Kulkarni J, Daskalakis ZJ. A randomized trial of low-frequency right-prefrontal-cortex transcranial magnetic stimulation as augmentation in treatment-resistant major depression. *Int J Neuropsychopharmacol* 2006; **9**(6)**:** 655-666.

26. Fitzgerald PB, Sritharan A, Daskalakis ZJ, de Castella AR, Kulkarni J, Egan G. A functional magnetic resonance imaging study of the effects of low frequency right prefrontal transcranial magnetic stimulation in depression. *J Clin Psychopharmacol* 2007; **27**(5)**:** 488-492.

27. Furtado CP, Hoy KE, Maller JJ, Savage G, Daskalakis ZJ, Fitzgerald PB. An Investigation of Medial Temporal Lobe Changes and Cognition Following Antidepressant Response: A Prospective rTMS Study. *Brain Stimulation* 2013; **6**(3)**:** 346-354.

28. Galletly C, Gill S, Clarke P, Burton C, Fitzgerald PB. A randomized trial comparing repetitive transcranial magnetic stimulation given 3 days/week and 5 days/week for the treatment of major depression: is efficacy related to the duration of treatment or the number of treatments? *Psychol Med* 2012; **42**(5)**:** 981-988.

29. Galletly CA, Carnell BL, Clarke P, Gill S. A Comparison of Right Unilateral and Sequential Bilateral Repetitive Transcranial Magnetic Stimulation for Major Depression A Naturalistic Clinical Australian Study. *Journal of Ect* 2017; **33**(1)**:** 58-62.

30. Ge R, Blumberger DM, Downar J, Daskalakis ZJ, Dipinto AA, Tham JCW *et al.* Abnormal functional connectivity within resting-state networks is related to rTMS-based therapy effects of treatment resistant depression: A pilot study. *Journal of Affective Disorders* 2017; **218**(pp 75-81).

31. Greenberg BD, George MS, Martin JD, Benjamin J, Schlaepfer TE, Altemus M *et al.* Effect of prefrontal repetitive transcranial magnetic stimulation in obsessive-compulsive disorder: a preliminary study. *Am J Psychiatry* 1997; **154**(6)**:** 867-869.

32. Grunhaus L, Dannon PN, Schreiber S, Dolberg OH, Amiaz R, Ziv R *et al.* Repetitive transcranial magnetic stimulation is as effective as electroconvulsive therapy in the treatment of nondelusional major depressive disorder: an open study. *Biol Psychiatry* 2000; **47**(4)**:** 314-324.

33. Grunhaus L, Polak D, Amiaz R, Dannon PN. Motor-evoked potential amplitudes elicited by transcranial magnetic stimulation do not differentiate between patients and normal controls. *International Journal of Neuropsychopharmacology* 2003; **6**(4)**:** 371-378.

34. Grunhaus L, Schreiber S, Dolberg OT, Polak D, Dannon PN. A randomized controlled comparison of electroconvulsive therapy and repetitive transcranial magnetic stimulation in severe and resistant nonpsychotic major depression. *Biol Psychiatry* 2003; **53**(4)**:** 324-331.

35. Gu P, Qiu Y-y, Li X-z. Clinical efficacy of repetitive transcranial magnetic stimulation in treatment of late life depression. *Chinese Journal of Clinical Psychology* 2017; **25**(3)**:** 588-590.

36. Hansen PE, Ravnkilde B, Videbech P, Clemmensen K, Sturlason R, Reiner M *et al.* Low-frequency repetitive transcranial magnetic stimulation inferior to electroconvulsive therapy in treating depression. *J ect* 2011; **27**(1)**:** 26-32.

37. Iwabuchi SJ, Auer DP, Lankappa ST, Palaniyappan L. Baseline effective connectivity predicts response to repetitive transcranial magnetic stimulation in patients with treatment-resistant depression. *Eur Neuropsychopharmacol* 2019; **29**(5)**:** 681-690.

38. Jahangard L, Tayebi M, Haghighi M, Ahmadpanah M, Holsboer-Trachsler E, Bahmani DS *et al.* Does rTMS on brain areas of mirror neurons lead to higher improvements on symptom severity and empathy compared to the rTMS standard procedure? - Results from a double-blind interventional study in individuals with major depressive disorders. *Journal of Affective Disorders* 2019; **257:** 527-535.

39. Janicak PG, Dowd SM, Martis B, Alam D, Beedle D, Krasuski J *et al.* Repetitive transcranial magnetic stimulation versus electroconvulsive therapy for major depression: preliminary results of a randomized trial. *Biol Psychiatry* 2002; **51**(8)**:** 659-667.

40. Jha S, Chadda RK, Kumar N, Bal CS. Brain SPECT guided repetitive transcranial magnetic stimulation (rTMS) in treatment resistant major depressive disorder. *Asian J Psychiatr* 2016; **21:** 1-6.

41. Johansson K, Adler M, Båve U, Ekman CJ, Lundberg J. Repetitive transcranial magnetic stimulation in major depression: A three-arm parallel-group dose-response randomized pilot trial. *Medicine (Baltimore)* 2021; **100**(14)**:** e25273.

42. Kazemi R, Rostami R, Khomami S, Horacek J, Brunovsky M, Novak T *et al.* Electrophysiological correlates of bilateral and unilateral repetitive transcranial magnetic stimulation in patients with bipolar depression. *Psychiatry Research* 2016; **240:** 364-375.

43. Keshtkar M, Ghanizadeh A, Firoozabadi A. Repetitive transcranial magnetic stimulation versus electroconvulsive therapy for the treatment of major depressive disorder, a randomized controlled clinical trial. *J ect* 2011; **27**(4)**:** 310-314.

44. Khayyer Z, Ngaosuvan L, Sikstrom S, Ghaderi AH. Transcranial direct current stimulation based on quantitative electroencephalogram combining positive psychotherapy for major depression. *Journal of Integrative Neuroscience* 2018; **17**(2)**:** 89-96.

45. Kindler J, Homan P, Flury R, Strik W, Dierks T, Hubl D. Theta burst transcranial magnetic stimulation for the treatment of auditory verbal hallucinations: results of a randomized controlled study. *Psychiatry Res* 2013; **209**(1)**:** 114-117.

46. Kito S, Miyazi M, Nakatani H, Matsuda Y, Yamazaki R, Okamoto T *et al.* Effectiveness of high-frequency left prefrontal repetitive transcranial magnetic stimulation in patients with treatment-resistant depression: A randomized clinical trial of 37.5-minute vs 18.75-minute protocol. *Neuropsychopharmacol Rep* 2019; **39**(3)**:** 203-208.

47. Kozel FA, Van Trees K, Larson V, Phillips S, Hashimie J, Gadbois B *et al.* One hertz versus ten hertz repetitive TMS treatment of PTSD: A randomized clinical trial. *Psychiatry Res* 2019; **273:** 153-162.

48. Kucerova H, Prikryl R, Navratilova P, Ceskova E, Ustohal L. Influencing cognitive deficit in patients with depressive disorder by means of repetitive transcranial magnetic stimulation (rTMS) under conditions of single blind randomized study. *Ceska a Slovenska Psychiatrie* 2008; **104**(5)**:** 224-229.

49. Kurimori M, Shiozawa P, Bikson M, Aboseria M, Cordeiro Q. Targeting negative symptoms in schizophrenia: Results from a proof-of-concept trial assessing prefrontal anodic tDCS protocol. *Schizophrenia Research* 2015; **166**(1-3)**:** 362-363.

50. Latif AA, Nasreldin M, Kader AA, Fathy H, Moussa S, Basheer M *et al.* A Randomized Study Comparing the Short-Term Neurocognitive Outcome of Electroconvulsive Therapy Versus Repetitive Transcranial Magnetic Stimulation in the Treatment of Patients With Depression. *J Psychiatr Pract* 2020; **26**(1)**:** 23-36.

51. Martis B, Alam D, Dowd SM, Hill SK, Sharma RP, Rosen C *et al.* Neurocognitive effects of repetitive transcranial magnetic stimulation in severe major depression. *Clinical Neurophysiology* 2003; **114**(6)**:** 1125-1132.

52. Mattai A, Miller R, Weisinger B, Greenstein D, Bakalar J, Tossell J *et al.* Tolerability of transcranial direct current stimulation in childhood-onset schizophrenia. *Brain Stimulation* 2011; **4**(4)**:** 275-280.

53. McClintock SM, Martin DM, Lisanby SH, Alonzo A, McDonald WM, Aaronson ST *et al.* Neurocognitive effects of transcranial direct current stimulation (tDCS) in unipolar and bipolar depression: Findings from an international randomized controlled trial. *Depress Anxiety* 2020; **37**(3)**:** 261-272.

54. McLoughlin DM, Mogg A, Eranti S, Pluck G, Purvis R, Edwards D *et al.* The clinical effectiveness and cost of repetitive transcranial magnetic stimulation versus electroconvulsive therapy in severe depression: a multicentre pragmatic randomised controlled trial and economic analysis. *Health Technol Assess* 2007; **11**(24)**:** 1-54.

55. Nasiri F, Mashhadi A, Bigdeli I, Chamanabad AG, Ellard KK. Augmenting the unified protocol for transdiagnostic treatment of emotional disorders with transcranial direct current stimulation in individuals with generalized anxiety disorder and comorbid depression: A randomized controlled trial. *J Affect Disord* 2020; **262:** 405-413.

56. Nongpiur A, Sinha VK, Praharaj SK, Goyal N. Theta-patterned, frequency-modulated priming stimulation enhances low-frequency, right prefrontal cortex repetitive transcranial magnetic stimulation (rTMS) in depression: a randomized, sham-controlled study. *J Neuropsychiatry Clin Neurosci* 2011; **23**(3)**:** 348-357.

57. Park S, Choi W-J, Kim S, Kim B, Son SJ, Roh D *et al.* Effects of transcranial direct current stimulation using miniaturized devices vs sertraline for depression in Korea: A 6 week, multicenter, randomized, double blind, active-controlled study. *Journal of Psychiatric Research* 2020; **127:** 42-47.

58. Poydasheva AG, Sinitsyn DO, Bakulin IS, Suponeva NA, Maslennikov NV, Tsukarzi EE *et al.* Target determination for transcranial magnetic stimulation in patients with a pharmacotherapy-resistant depressive episode based on the individual parameters of resting-state functional magnetic resonance imaging (a pilot blind controlled trial). *Nevrologiya, Neiropsikhiatriya, Psikhosomatika* 2019; **11**(4)**:** 44-50.

59. Price GW, Lee JW, Garvey CA, Gibson N. The use of background EEG activity to determine stimulus timing as a means of improving rTMS efficacy in the treatment of depression: a controlled comparison with standard techniques. *Brain Stimul* 2010; **3**(3)**:** 140-152.

60. Ray P, Sinha VK, Tikka SK. Adjuvant low-frequency rTMS in treating auditory hallucinations in recent-onset schizophrenia: a randomized controlled study investigating the effect of high-frequency priming stimulation. *Ann Gen Psychiatry* 2015; **14:** 8.

61. Reyes-López J, Ricardo-Garcell J, Armas-Castañeda G, García-Anaya M, Arango-De Montis I, González-Olvera JJ *et al.* Clinical improvement in patients with borderline personality disorder after treatment with repetitive transcranial magnetic stimulation: preliminary results. *Braz J Psychiatry* 2018; **40**(1)**:** 97-104.

62. Richieri R, Boyer L, Padovani R, Adida M, Colavolpe C, Mundler O *et al.* Equivalent brain SPECT perfusion changes underlying therapeutic efficiency in pharmacoresistant depression using either high-frequency left or low-frequency right prefrontal rTMS. *Prog Neuropsychopharmacol Biol Psychiatry* 2012; **39**(2)**:** 364-370.

63. Rosa MA, Gattaz WF, Pascual-Leone A, Fregni F, Rosa MO, Rumi DO *et al.* Comparison of repetitive transcranial magnetic stimulation and electroconvulsive therapy in unipolar non-psychotic refractory depression: a randomized, single-blind study. *Int J Neuropsychopharmacol* 2006; **9**(6)**:** 667-676.

64. Rossini D, Lucca A, Magri L, Malaguti A, Smeraldi E, Colombo C *et al.* A symptom-specific analysis of the effect of high-frequency left or low-frequency right transcranial magnetic stimulation over the dorsolateral prefrontal cortex in major depression. *Neuropsychobiology* 2010; **62**(2)**:** 91-97.

65. Rybak M, Bruno R, Turnier-Shea Y, Pridmore S. An attempt to increase the rate and magnitude of the antidepressant effect of transcranial magnetic stimulation (TMS). A pilot study. *German Journal of Psychiatry* 2005; **8**(4)**:** 59-65.

66. Slotema CW, Blom JD, de Weijer AD, Hoek HW, Sommer IE. Priming does not enhance the efficacy of 1 Hertz repetitive transcranial magnetic stimulation for the treatment of auditory verbal hallucinations: results of a randomized controlled study. *Brain Stimul* 2012; **5**(4)**:** 554-559.

67. Sokhadze EM, Lamina EV, Casanova EL, Kelly DP, Opris I, Tasman A *et al.* Exploratory Study of rTMS Neuromodulation Effects on Electrocortical Functional Measures of Performance in an Oddball Test and Behavioral Symptoms in Autism. *Front Syst Neurosci* 2018; **12:** 20.

68. Tastevin M, Baumstarck K, Groppi F, Cermolacce M, Lagrange G, Lançon C *et al.* Double cone coil rTMS efficacy for treatment-resistant depression: A prospective randomized controlled trial. *Brain Stimul* 2020; **13**(1)**:** 256-258.

69. Trojak B, Meille V, Jonval L, Schuffenecker N, Haffen E, Schwan R *et al.* Interest of targeting either cortical area Brodmann 9 or 46 in rTMS treatment for depression: A preliminary randomized study. *Clinical Neurophysiology* 2014; **125**(12)**:** 2384-2389.

70. Udupa K, Thirthalli J, Sathyaprabha TN, Kishore KR, Raju TR, Gangadhar BN. Differential actions of antidepressant treatments on cardiac autonomic alterations in depression: A prospective comparison. *Asian J Psychiatr* 2011; **4**(2)**:** 100-106.

71. Ustohal L, Kucerova HP, Prikryl R, Stehnova I, Hublova V, Mayerova M *et al.* Repetitive Transcranial Magnetic Stimulation in the Treatment of Depressive Disorder - a Randomized, Single-blind, Antidepressants-controlled Study. *Cesk Slov Neurol Neurochir* 2014; **77**(5)**:** 602-607.

72. Valiulis V, Gerulskis G, Dapšys K, Valavičiūtė K, Šiurkutė A, Mačiulis V. The use of MR‑less MNI based neuronavigation for 10 Hz rTMS depression therapy: electrophysiological and clinical implications. *Acta Neurobiol Exp (Wars)* 2018; **78**(3)**:** 271-280.

73. van Lutterveld R, Koops S, Schutter D, Geertsema E, Stam CJ, Kahn RS *et al.* The effect of rTMS on auditory hallucinations: Clues from an EEG-rTMS study. *Schizophrenia Research* 2012; **137**(1-3)**:** 174-179.

74. Wang XM, Yang DB, Yu YF, Huang H, Zhao XQ. A controlled study of the treatment of repetitive transcranial magnetic stimulation in patients with major depression. *Chinese Journal of Clinical Rehabilitation* 1770; **8**(9)**:** 1770-1771.

75. Yuan J, Chen YM, Yu PL, Luo F, Gao YX, Chen J *et al.* Effect of magnetic stimulation of Shenmen point on cognitive function of chronic insomnia A randomized controlled clinical trial. *Medicine* 2020; **99**(51)**:** 5.

76. Zhang T, Huang Y, Jin Y, Ma X, Liu Z. Treatment for Major Depressive Disorder by Repetitive Transcranial Magnetic Stimulation in Different Parameters: A Randomized Double-Blinded Controlled Trial. *Front Psychiatry* 2021; **12:** 623765.

77. Zhao X, Li Y, Tian Q, Zhu B, Zhao Z. Repetitive transcranial magnetic stimulation increases serum brain-derived neurotrophic factor and decreases interleukin-1β and tumor necrosis factor-α in elderly patients with refractory depression. *J Int Med Res* 2019; **47**(5)**:** 1848-1855.

Not a psychiatric condition

1. Altunrende B, Yildiz S, Cevik A, Yildiz N. Repetitive transcranial magnetic stimulation in restless legs syndrome: preliminary results. *Neurol Sci* 2014; **35**(7)**:** 1083-1088.

2. Arias P, Vivas J, Grieve KL, Cudeiro J. Double-blind, randomized, placebo controlled trial on the effect of 10 days low-frequency rTMS over the vertex on sleep in Parkinson's disease. *Sleep Med* 2010; **11**(8)**:** 759-765.

3. Broeder S, Heremans E, Pinto Pereira M, Nackaerts E, Meesen R, Verheyden G *et al.* Does transcranial direct current stimulation during writing alleviate upper limb freezing in people with Parkinson's disease? A pilot study. *Hum Mov Sci* 2019; **65**.

4. Bystad M, Gronli O, Rasmussen ID, Gundersen N, Nordvang L, Wang-Iversen H *et al.* Transcranial direct current stimulation as a memory enhancer in patients with Alzheimer's disease: a randomized, placebo-controlled trial. *Alzheimers Res Ther* 2016; **8:** 7.

5. Cha YH, Deblieck C, Wu AD. Double-Blind Sham-Controlled Crossover Trial of Repetitive Transcranial Magnetic Stimulation for Mal de Debarquement Syndrome. *Otol Neurotol* 2016; **37**(6)**:** 805-812.

6. Chalah MA, Riachi N, Ahdab R, Mhalla A, Abdellaoui M, Creange A *et al.* Effects of left DLPFC versus right PPC tDCS on multiple sclerosis fatigue. *J Neurol Sci* 2017; **372:** 131-137.

7. Chung CLH, Mak MKY, Hallett M. Transcranial Magnetic Stimulation Promotes Gait Training in Parkinson Disease. *Ann Neurol* 2020; **88**(5)**:** 933-945.

8. da Silva DCL, Lemos T, Ferreira AD, Horsczaruk CHR, Pedron CA, Rodrigues ED *et al.* Effects of Acute Transcranial Direct Current Stimulation on Gait Kinematics of Individuals With Parkinson Disease. *Top Geriatr Rehabil* 2018; **34**(4)**:** 262-268.

9. de Albuquerque LL, Pantovic M, Clingo M, Fischer K, Jalene S, Landers M *et al.* An Acute Application of Cerebellar Transcranial Direct Current Stimulation Does Not Improve Motor Performance in Parkinson's Disease. *Brain Sci* 2020; **10**(10)**:** 15.

10. Fridriksson J, Rorden C, Elm J, Sen S, George MS, Bonilha L. Transcranial Direct Current Stimulation vs Sham Stimulation to Treat Aphasia After Stroke: A Randomized Clinical Trial. *JAMA Neurol* 2018; **75**(12)**:** 1470-1476.

11. Gaede G, Tiede M, Lorenz I, Brandt AU, Pfueller C, Dorr J *et al.* Safety and preliminary efficacy of deep transcranial magnetic stimulation in MS-related fatigue. *Neurol-Neuroimmunol Neuroinflammation* 2018; **5**(1)**:** 8.

12. Grecco LAC, Duarte NAC, Zanon N, Galli M, Fregni F, Oliveira CS. Effect of a single session of transcranial direct-current stimulation on balance and spatiotemporal gait variables in children with cerebral palsy: A randomized sham-controlled study. *Rev Bras Fisioter* 2014; **18**(5)**:** 419-427.

13. Grohs MN, Craig BT, Kirton A, Dewey D. Effects of transcranial direct current stimulation on motor function in children 8-12 years with developmental coordination disorder: A randomized controlled trial. [References]. *Frontiers in Human Neuroscience* 2020; **14:** 608131.

14. Hamoudi M, Schambra HM, Fritsch B, Schoechlin-Marx A, Weiller C, Cohen LG *et al.* Transcranial Direct Current Stimulation Enhances Motor Skill Learning but Not Generalization in Chronic Stroke. *Neurorehabil Neural Repair* 2018; **32**(4-5)**:** 295-308.

15. Inagawa T, Yokoi Y, Narita Z, Maruo K, Okazaki M, Nakagome K. Safety and Feasibility of Transcranial Direct Current Stimulation for Cognitive Rehabilitation in Patients With Mild or Major Neurocognitive Disorders: A Randomized Sham-Controlled Pilot Study. *Front Hum Neurosci* 2019; **13:** 273.

16. Lu H, Chan SSM, Chan WC, Lin C, Cheng CPW, Linda Chiu Wa L. Randomized controlled trial of TDCS on cognition in 201 seniors with mild neurocognitive disorder. *Ann Clin Transl Neurol* 2019; **6**(10)**:** 1938-1948.

17. Saebipour MR, Joghataei MT, Yoonessi A, Sadeghniiat-Haghighi K, Khalighinejad N, Khademi S. Slow oscillating transcranial direct current stimulation during sleep has a sleep-stabilizing effect in chronic insomnia: a pilot study. *J Sleep Res* 2015; **24**(5)**:** 518-525.

Not an RCT

1. Aleman A, Slotema CW, Sommer IE. rTMS deserves a fair chance as a novel treatment for depression. *Acta Psychiatrica Scandinavica* 2014; **130**(5)**:** 324-325.

*Reason for exclusion: Editorial comment*

2. Anderson BS, Kavanagh K, Borckardt JJ, Nahas ZH, Kose S, Lisanby SH *et al.* Decreasing procedural pain over time of left prefrontal rTMS for depression: initial results from the open-label phase of a multi-site trial (OPT-TMS). *Brain Stimul* 2009; **2**(2)**:** 88-92.

*Reason for exclusion: Open label study*

3. Andrade C. Transcranial direct current stimulation for refractory auditory hallucinations in schizophrenia. *J Clin Psychiatry* 2013; **74**(11)**:** e1054-1058.

*Reason for exclusion: Opinion piece*

4. Avery DH, Isenberg KE, Sampson SM, Janicak PG, Lisanby SH, Maixner DF *et al.* Transcranial magnetic stimulation in the acute treatment of major depressive disorder: Clinical response in an open-label extension trial. *J Clin Psychiatry* 2008; **69**(3)**:** 441-451.

*Reason for exclusion: Open label study*

5. Bakker N, Shahab S, Giacobbe P, Blumberger DM, Daskalakis ZJ, Kennedy SH *et al.* rTMS of the dorsomedial prefrontal cortex for major depression: safety, tolerability, effectiveness, and outcome predictors for 10 Hz versus intermittent theta-burst stimulation. *Brain Stimul* 2015; **8**(2)**:** 208-215.

*Reason for exclusion: Review article*

6. Bation R, Poulet E, Haesebaert F, Saoud M, Brunelin J. Transcranial direct current stimulation in treatment-resistant obsessive-compulsive disorder: An open-label pilot study. *Prog Neuropsychopharmacol Biol Psychiatry* 2016; **65:** 153-157.

*Reason for exclusion: Open label study*

7. Bozzay ML, Primack J, Barredo J, Philip NS. Transcranial magnetic stimulation to reduce suicidality - A review and naturalistic outcomes. *J Psychiatr Res* 2020; **125:** 106-112.

*Reason for exclusion: Review article*

8. Brunoni AR, Ferrucci R, Bortolomasi M, Vergari M, Tadini L, Boggio PS *et al.* Transcranial direct current stimulation (tDCS) in unipolar vs. bipolar depressive disorder. *Progress in Neuro-Psychopharmacology & Biological Psychiatry* 2011; **35**(1)**:** 96-101.

*Reason for exclusion: Open label study*

9. Chen XG, Ji GJ, Zhu CY, Bai XM, Wang L, He KL *et al.* Neural Correlates of Auditory Verbal Hallucinations in Schizophrenia and the Therapeutic Response to Theta-Burst Transcranial Magnetic Stimulation. *Schizophr Bull* 2019; **45**(2)**:** 474-483.

*Reason for exclusion: Naturalistic study*

10. Chow ZR. Sham Treatment Is as Effective for Treatment-Resistant Depression as Repetitive Transcranial Magnetic Stimulation. *JAMA Psychiatry* 2019; **76**(1).

*Reason for exclusion: Editorial comment*

11. Ciobanu C, Girard M, Marin B, Labrunie A, Malauzat D. rTMS for pharmacoresistant major depression in the clinical setting of a psychiatric hospital: Effectiveness and effects of age. *Journal of Affective Disorders* 2013; **150**(2)**:** 677-681.

*Reason for exclusion: Open label study*

12. Cordes J, Mobascher A, Arends M, Agelink MW, Klimke A. [A new method for the treatment of depression: repetitive transcranial magnetic stimulation]. *Deutsche Medizinische Wochenschrift* 2005; **130**(14)**:** 889-892.

*Reason for exclusion:*

13. Croarkin PE, Nakonezny PA, Wall CA, Murphy LL, Sampson SM, Frye MA *et al.* Transcranial magnetic stimulation potentiates glutamatergic neurotransmission in depressed adolescents. *Psychiatry Research Neuroimaging* 2016; **247**(pp 25-33).

*Reason for exclusion: Review article*

14. Demeulemeester M, Amad A, Bubrovszky M, Pins D, Thomas P, Jardri R. What is the real effect of 1-Hz repetitive transcranial magnetic stimulation on hallucinations? Controlling for publication bias in neuromodulation trials. *Biological Psychiatry* 2012; **71**(6)**:** e15-16.

*Reason for exclusion: Review article*

15. Dhami P, Knyahnytska Y, Atluri S, Lee J, Courtney DB, Croarkin PE *et al.* Feasibility and clinical effects of theta burst stimulation in youth with major depressive disorders: An open-label trial. *J Affect Disord* 2019; **258:** 66-73.

*Reason for exclusion: Open label study*

16. Dilkov D. General health in obsessive-compulsive disorder and transcranial magnetic stimulation. *General Medicine* 2019; **21**(1)**:** 19-24.

*Reason for exclusion: Review article*

17. Downar J, Blumberger DM, Daskalakis ZJ. Repetitive transcranial magnetic stimulation: an emerging treatment for medication-resistant depression. *Can Med Assoc J* 2016; **188**(16)**:** 1175-1177.

*Reason for exclusion: Review article*

18. Etcheverrigaray F, Bulteau S, Machon LO, Riche VP, Mauduit N, Leux C *et al.* Treating depression with repetitive transcranial magnetic stimulation (rTMS): Which repayment of a leading activity in psychiatry? *Rev Epidemiol Sante Publique* 2017; **65**(3)**:** 241-246.

*Reason for exclusion: Review article*

19. Fitzgerald PB. Is Maintenance Repetitive Transcranial Magnetic Stimulation for Patients With Depression a Valid Therapeutic Strategy? *Clinical Pharmacology & Therapeutics* 2019; **106**(4)**:** 723-725.

*Reason for exclusion: Review article*

20. Fitzgerald PB, McQueen S, Herring S, Hoy K, Segrave R, Kulkarni J *et al.* A study of the effectiveness of high-frequency left prefrontal cortex transcranial magnetic stimulation in major depression in patients who have not responded to right-sided stimulation. *Psychiatry Res* 2009; **169**(1)**:** 12-15.

*Reason for exclusion: Pooled analysis*

21. Ford H, Hahn L, Clarke P, Gill S, Carnell B, Galletly C. A comparison of 15 minute vs 30 minute repetitive transcranial magnetic stimulation sessions for treatment resistant depression - are longer treatment sessions more effective? *J Affect Disord* 2021; **282:** 974-978.

*Reason for exclusion: Naturalistic study*

22. George MS. Transcranial magnetic stimulation: a stimulating new method for treating depression, but saddled with the same old problems. *International Journal of Neuropsychopharmacology* 2006; **9**(6)**:** 637-640.

*Reason for exclusion: Review article*

23. George MS, Post RM. Daily Left Prefrontal Repetitive Transcranial Magnetic Stimulation for Acute Treatment of Medication-Resistant Depression. *Am J Psychiat* 2011; **168**(4)**:** 356-364.

*Reason for exclusion: Review article*

24. George MS, Speer AM, Molloy M, Nahas Z, Teneback CC, Risch SC *et al.* Low frequency daily left prefrontal rTMS improves mood in bipolar depression: A placebo-controlled case report. *Hum Psychopharmacol-Clin Exp* 1998; **13**(4)**:** 271-275.

*Reason for exclusion: Case study*

25. Hadas I, Sun Y, Lioumis P, Zomorrodi R, Jones B, Voineskos D *et al.* Association of Repetitive Transcranial Magnetic Stimulation Treatment With Subgenual Cingulate Hyperactivity in Patients With Major Depressive Disorder: A Secondary Analysis of a Randomized Clinical Trial. *JAMA Netw Open* 2019; **2**(6)**:** e195578.

*Reason for exclusion: Diagnostic study*

26. Haesebaert F, Mondino M, Saoud M, Poulet E, Brunelin J. Efficacy and safety of fronto-temporal transcranial random noise stimulation (tRNS) in drug-free patients with schizophrenia: A case study. *Schizophrenia Research* 2014; **159**(1)**:** 251-252.

*Reason for exclusion: Case study*

27. Hernandez MJ, Reljic T, Van Trees K, Phillips S, Hashimie J, Bajor L *et al.* Impact of Comorbid PTSD on Outcome of Repetitive Transcranial Magnetic Stimulation (TMS) for Veterans With Depression. *J Clin Psychiatry* 2020; **81**(4).

*Reason for exclusion: Naturalistic study*

28. Jin XL, Xu WQ, Le YJ, Dai XK. Long-term Effectiveness of Modified Electroconvulsive Therapy Compared With Repetitive Transcranial Magnetic Stimulation for the Treatment of Recurrent Major Depressive Disorder. *J Nerv Ment Dis* 2016; **204**(6)**:** 479-482.

*Reason for exclusion: Retrospective study*

29. Karstens MI, Cohen Kadosh R. Targeting neuronal correlates of executive function in ADHD using brain stimulation. *Clinical Neurophysiology* 1142; **131**(5)**:** 1142-1143.

*Reason for exclusion: Editorial comment*

30. Kim J, Gerretsen P. Improving insight to facilitate antipsychotic medication adherence in patients with schizophrenia. *Clinical Neurophysiology* 1968; **131**(8)**:** 1968-1970.

*Reason for exclusion: Editorial comment*

31. Kim M, Yoon YB, Lee TH, Lee TY, Kwon JS. The effect of tDCS on auditory hallucination and P50 sensory gating in patients with schizophrenia: A pilot study. *Schizophrenia Research* 2018; **192**(pp 469-470).

*Reason for exclusion: Open label study*

32. Koops S, Slotema CW, Kos C, Bais L, Aleman A, Blom JD *et al.* Predicting response to rTMS for auditory hallucinations: Younger patients and females do better. *Schizophrenia Research* 2018; **195:** 583-584.

*Reason for exclusion: Pooled analysis*

33. Lee TY, Lee J, Kim M, Kwon JS. The effect of transcranial direct current stimulation on auditory hallucination in patients with schizophrenia. *Schizophrenia Research* 2018; **192:** 489-490.

*Reason for exclusion: Review article*

34. Loo C, Martin D. *Transcranial direct current stimulation*. John Wiley & Sons Ltd: Chichester, 2016, 227-243pp.

*Reason for exclusion: Review article*

35. Loo C, McFarquhar T, Walter G. Transcranial magnetic stimulation in adolescent depression. *Australas Psychiatry* 2006; **14**(1)**:** 81-85.

*Reason for exclusion: Report of two participants only*

36. Magnezi R, Aminov E, Shmuel D, Dreifuss M, Dannon P. Comparison between neurostimulation techniques repetitive transcranial magnetic stimulation vs electroconvulsive therapy for the treatment of resistant depression: patient preference and cost-effectiveness. *Patient Prefer Adherence* 2016; **10:** 1481-1487.

*Reason for exclusion: Medical record review*

38. Miron JP, Feffer K, Cash RFH, Derakhshan D, Kim JMS, Fettes P *et al.* Safety, tolerability and effectiveness of a novel 20 Hz rTMS protocol targeting dorsomedial prefrontal cortex in major depression: An open-label case series. *Brain Stimulation* 1319; **12**(5)**:** 1319-1321.

*Reason for exclusion: Open label study*

39. Nursey J, Sbisa A, Knight H, Ralph N, Cowlishaw S, Forbes D *et al.* Exploring Theta Burst Stimulation for Post-traumatic Stress Disorder in Australian Veterans-A Pilot Study. *Mil Med* 2020; **185**(9-10)**:** e1770-e1778.

*Reason for exclusion: Case series*

40. Padinjareveettil AMT, Rogers J, Loo C, Martin D. Transcranial Direct Current Stimulation to Enhance Cognitive Remediation in Schizophrenia. *Brain Stimulation* 2015; **8**(2)**:** 307-309.

*Reason for exclusion: Case study*

41. Pallanti S, Marras A, Salerno L, Makris N, Hollander E. Better than treated as usual: Transcranial magnetic stimulation augmentation in selective serotonin reuptake inhibitor-refractory obsessive-compulsive disorder, mini-review and pilot open-label trial. *Journal of Psychopharmacology* 2016; **30**(6)**:** 568-578.

*Reason for exclusion: Review and open label study*

42. Paul C, Pascual-Leone A, Stern AP. Varied Antidepressant Response and Subjective Experience Across 3 Different Repetitive Transcranial Magnetic Stimulation Devices A Case Report. *Journal of Ect* 2017; **33**(4)**:** E34-E35.

*Reason for exclusion: Case study*

43. Pereira BD, Tortella G, Lafer B, Nunes P, Bensenor IM, Lotufo PA *et al.* The Bipolar Depression Electrical Treatment Trial (BETTER): Design, Rationale, and Objectives of a Randomized, Sham-Controlled Trial and Data from the Pilot Study Phase. *Neural Plast* 2015; **2015:** 10.

*Reason for exclusion: Protocol only*

44. Philip NS, Dunner DL, Dowd SM, Aaronson ST, Brock DG, Carpenter LL *et al.* "Can medication free, treatment-resistant, depressed patients who initially respond to TMS be maintained off medications? A prospective, 12-month multisite randomized pilot study": Erratum. *Brain Stimulation* 2016; **9**(4)**:** 639.

*Reason for exclusion: Erratum*

45. Plewnia C, Padberg F. Transcranial and invasive brain stimulation for depression. *Nervenarzt* 2012; **83**(8)**:** 1006-1012.

*Reason for exclusion: Review article*

46. Prikryl R, Kasparek T, Skotakova S, Ustohal L, Kucerova H, Ceskova E. Treatment of negative symptoms of schizophrenia using repetitive transcranial magnetic stimulation in a double-blind, randomized controlled study (vol 95, pg 151, 2007). *Schizophrenia Research* 2008; **99**(1-3)**:** 380-381.

*Reason for exclusion: Erratum*

47. Prikryl R, Skotakova S, Kasparek T, Ceskova E, Kucerova H, Ustohal L. Influencing negative symptoms of schizophrenia with repetitive transcranial magnetic stimulation: a case study. *Acta Neuropsychiatr* 2007; **19**(1)**:** 53-55.

*Reason for exclusion: Case study*

48. Prikryl R, Ustohal L, Prikrylova-Kucerova H, Cermakova I, Ceskova E. Effects of Sequential Frontotemporal Repetitive Transcranial Magnetic Stimulation (rTMS) on Schizophrenia. *Act Nerv Super Rediviva* 2010; **52**(1)**:** 7.

*Reason for exclusion: Case study*

49. Rosenich E, Gill S, Clarke P, Paterson T, Hahn L, Galletly C. Does rtms reduce depressive symptoms in young people who have not responded to antidepressants? *Early Intervention in Psychiatry* 2018; (Pagination).

*Reason for exclusion: Retrospective study*

50. Schulze L, Remington G, Giacobbe P, Kennedy SH, Blumberger DM, Daskalakis ZJ *et al.* Effect of antipsychotic pharmacotherapy on clinical outcomes of intermittent theta-burst stimulation for refractory depression. *J Psychopharmacol* 2017; **31**(3)**:** 312-319.

*Reason for exclusion: Review article*

51. Shiozawa P, Da Silva ME, Cordeiro Q, Fregni F, Brunoni AR. Transcranial direct current stimulation (tDCS) for the treatment of persistent visual and auditory hallucinations in schizophrenia: A case study. *Brain Stimulation* 2013; **6**(5)**:** 831-833.

*Reason for exclusion: Case study*

52. Spampinato C, Aguglia E, Concerto C, Pennisi M, Lanza G, Bella R *et al.* Transcranial Magnetic Stimulation in the Assessment of Motor Cortex Excitability and Treatment of Drug-Resistant Major Depression. *IEEE Trans Neural Syst Rehabil Eng* 2013; **21**(3)**:** 391-403.

*Reason for exclusion: No randomisation*

53. Tamas RL, Menkes D, El-Mallakh RS. Stimulating research: A prospective, randomized, double-blind, sham-controlled study of slow transcranial magnetic stimulation in depressed bipolar patients. *The Journal of Neuropsychiatry and Clinical Neurosciences* 2007; **19**(2)**:** 198-199.

*Reason for exclusion: Unequal randomisation*

54. Taylor SF, Bhati MT, Dubin MJ, Hawkins JM, Lisanby SH, Morales O *et al.* A naturalistic, multi-site study of repetitive transcranial magnetic stimulation therapy for depression. *Journal of Affective Disorders* 2017; **208:** 284-290.

*Reason for exclusion: Naturalistic study*

55. Terhune DB, Cohen Kadosh R. Modulating cognitive control in major depression with transcranial electrical stimulation. *Biological Psychiatry* 2013; **73**(7)**:** 595-596.

*Reason for exclusion: Commentary*

56. Thomas TA, Hernandez EI. Transcranial direct current stimulation for the treatment of major depression. *Psychopharm Review* 2013; **48**(12)**:** 89-96.

*Reason for exclusion: Review article*

57. Thomas-Ollivier V, Deschamps T, Bulteau S, Le Gall F, Pichot A, Valriviere P *et al.* Effect of repetitive transcranial magnetic stimulation on psychomotor retardation in major depression: A pilot feasibility study. *Journal of Neuropsychiatry and Clinical Neurosciences* 2016; **28**(1)**:** 62-65.

*Reason for exclusion: Open label study*

58. Vanderhasselt MA, De Raedt R, Leyman L, Baeken C. Acute effects of repetitive transcranial magnetic stimulation on attentional control are related to antidepressant outcomes. *Journal of Psychiatry and Neuroscience* 2009; **34**(2)**:** 119-126.

*Reason for exclusion: No randomisation*

59. Voineskos D, Daskalakis ZJ. A primer on the treatment of schizophrenia through repetitive transcranial magnetic stimulation. *Expert Review of Neurotherapeutics* 2013; **13**(10)**:** 1079-1082.

*Reason for exclusion: Review article*

60. Yuan J, Wang H, Chen J, Lei Y, Wan Z, Zhao Y *et al.* Effect of low frequency repetitive magnetic stimulation at Shenmen (HT7) on sleep quality in patients with chronic insomnia. *Medicine (Baltimore)* 2020; **99**(30)**:** e21292.

*Reason for exclusion: Study protocol only*

61. Zhang YL, Liang W, Yang SC, Dai P, Shen LJ, Wang CH. Repetitive transcranial magnetic stimulation for hallucination in schizophrenia spectrum disorders A meta-analysis. *Neural Regen Res* 2013; **8**(28)**:** 2666-2676.

*Reason for exclusion: Review article*

62. Zrenner B, Zrenner C, Gordon PC, Belardinelli P, McDermott EJ, Soekadar SR *et al.* Brain oscillation-synchronized stimulation of the left dorsolateral prefrontal cortex in depression using real-time EEG-triggered TMS. *Brain Stimul* 2020; **13**(1)**:** 197-205.

*Reason for exclusion: No randomisation*

Subanalysis

1. Alonzo A, Chan G, Martin D, Mitchell PB, Loo C. Transcranial direct current stimulation (tDCS) for depression: Analysis of response using a three-factor structure of the Montgomery-Asberg depression rating scale. *Journal of Affective Disorders* 2013; **150**(1)**:** 91-95.

*Original article:* Loo CK, Alonzo A, Martin D, Mitchell PB, Galvez V, Sachdev P. Transcranial direct current stimulation for depression: 3-week, randomised, sham-controlled trial. *Br J Psychiatry* 2012; **200**(1)**:** 52-59.

2. Assaf M, Rabany L, Zertuche L, Bragdon L, Tolin D, Goethe J *et al.* Neural functional architecture and modulation during decision making under uncertainty in individuals with generalized anxiety disorder. *Brain Behav* 2018; **8**(8)**:** e01015.

*Original article:* Diefenbach GJ, Bragdon LB, Zertuche L, Hyatt CJ, Hallion LS, Tolin DF *et al.* Repetitive transcranial magnetic stimulation for generalised anxiety disorder: a pilot randomised, double-blind, sham-controlled trial. *Br J Psychiatry* 2016; **209**(3)**:** 222-228.

3. Avery DH, Holtzheimer PE, Fawaz W, Russo J, Neumaier J, Dunner DL *et al.* Transcranial magnetic stimulation reduces pain in patients with major depression a sham-controlled study. *J Nerv Ment Dis* 2007; **195**(5)**:** 378-381.

*Original article:* Avery DH, Holtzheimer IPE, Fawaz W, Russo J, Neumaier J, Dunner DL *et al.* A controlled study of repetitive transcranial magnetic stimulation in medication-resistant major depression. *Biological Psychiatry* 2006; **59**(2)**:** 187-194.

4. Baeken C, Duprat R, Wu GR, De Raedt R, van Heeringen K. Subgenual Anterior Cingulate-Medial Orbitofrontal Functional Connectivity in Medication-Resistant Major Depression: A Neurobiological Marker for Accelerated Intermittent Theta Burst Stimulation Treatment? *Biol Psychiatry Cogn Neurosci Neuroimaging* 2017; **2**(7)**:** 556-565.

*Original article:* Duprat R, Desmyter S, De Raedt R, van Heeringen K, Van den Abbeele D, Tandt H *et al.* Accelerated intermittent theta burst stimulation treatment in medication-resistant major depression: A fast road to remission? *Journal of Affective Disorders* 2016; **200:** 6-14.

5. Baeken C, Marinazzo D, Everaert H, Wu G-R, Van Hove C, Audenaert K *et al.* The impact of accelerated HF-rTMS on the subgenual anterior cingulate cortex in refractory unipolar major depression: Insights from 18FDG PET brain imaging. *Brain Stimulation* 2015; **8**(4)**:** 808-815.

*Original article:* Baeken C, Vanderhasselt M-A, Remue J, Herremans S, Vanderbruggen N, Zeeuws D *et al.* Intensive HF-rTMS treatment in refractory medication-resistant unipolar depressed patients. *Journal of Affective Disorders* 2013; **151**(2)**:** 625-631.

6. Baeken C, Marinazzo D, Wu G-R, Van Schuerbeek P, De Mey J, Marchetti I *et al.* Accelerated HF-rTMS in treatment-resistant unipolar depression: Insights from subgenual anterior cingulate functional connectivity. *The World Journal of Biological Psychiatry* 2014; **15**(4)**:** 286-297.

*Original article:* Baeken C, Vanderhasselt M-A, Remue J, Herremans S, Vanderbruggen N, Zeeuws D *et al.* Intensive HF-rTMS treatment in refractory medication-resistant unipolar depressed patients. *Journal of Affective Disorders* 2013; **151**(2)**:** 625-631.

7. Baeken C, Wu GR, van Heeringen K. Placebo aiTBS attenuates suicidal ideation and frontopolar cortical perfusion in major depression. *Transl Psychiatry* 2019; **9**(1)**:** 38.

*Original article:* Desmyter S, Duprat R, Baeken C, Van Autreve S, Audenaert K, van Heeringen K. Accelerated Intermittent Theta Burst Stimulation for Suicide Risk in Therapy-Resistant Depressed Patients: A Randomized, Sham-Controlled Trial. *Front Hum Neurosci* 2016; **10:** 480.

8. Bais L, Liemburg E, Vercammen A, Bruggeman R, Knegtering H, Aleman A. Effects of low frequency rTMS treatment on brain networks for inner speech in patients with schizophrenia and auditory verbal hallucinations. *Prog Neuropsychopharmacol Biol Psychiatry* 2017; **78:** 105-113.

*Original article:* Bais L, Vercammen A, Stewart R, van Es F, Visser B, Aleman A *et al.* Short and long term effects of left and bilateral repetitive transcranial magnetic stimulation in schizophrenia patients with auditory verbal hallucinations: a randomized controlled trial. *PLoS One* 2014; **9**(10)**:** e108828.

9. Bose A, Nawani H, Agarwal SM, Shivakumar V, Kalmady SV, Shenoy S *et al.* Effect of fronto-temporal transcranial direct current stimulation on corollary discharge in schizophrenia: A randomized, double-blind, sham-controlled mediation analysis study. *Schizophr Res* 2019; **204:** 411-412.

*Original article:* Bose A, Shivakumar V, Agarwal SM, Kalmady SV, Shenoy S, Sreeraj VS *et al.* Efficacy of fronto-temporal transcranial direct current stimulation for refractory auditory verbal hallucinations in schizophrenia: A randomized, double-blind, sham-controlled study. *Schizophr Res* 2018; **195:** 475-480.

10. Caeyenberghs K, Duprat R, Leemans A, Hosseini H, Wilson PH, Klooster D *et al.* Accelerated intermittent theta burst stimulation in major depression induces decreases in modularity: A connectome analysis. *Netw Neurosci* 2019; **3**(1)**:** 157-172.

*Original article:* Desmyter S, Duprat R, Baeken C, Van Autreve S, Audenaert K, van Heeringen K. Accelerated Intermittent Theta Burst Stimulation for Suicide Risk in Therapy-Resistant Depressed Patients: A Randomized, Sham-Controlled Trial. *Front Hum Neurosci* 2016; **10:** 480.

11. Cosmo C, Baptista AF, de Sena EP. Contribution of transcranial direct current stimulation on inhibitory control to assess the neurobiological aspects of attention deficit hyperactivity disorder: randomized controlled trial. *JMIR Res Protoc* 2015; **4**(2)**:** e56.

*Original article:* Cosmo C, Baptista AF, de Araújo AN, do Rosário RS, Miranda JG, Montoya P *et al.* A Randomized, Double-Blind, Sham-Controlled Trial of Transcranial Direct Current Stimulation in Attention-Deficit/Hyperactivity Disorder. *PLoS One* 2015; **10**(8)**:** e0135371.

12. Cosmo C, Ferreira C, Garcia J, Miranda V, do Rosario RS, Baptista AF *et al.* Spreading effect of tDCS in individuals with attention-deficit/hyperactivity disorder as shown by functional cortical networks: a randomized, double-blind, sham-controlled trial. *Front Psychiatry* 2015; **6:** 9.

*Original article:* Cosmo C, Baptista AF, de Araújo AN, do Rosário RS, Miranda JG, Montoya P *et al.* A Randomized, Double-Blind, Sham-Controlled Trial of Transcranial Direct Current Stimulation in Attention-Deficit/Hyperactivity Disorder. *PLoS One* 2015; **10**(8)**:** e0135371.

13. Diefenbach GJ, Assaf M, Goethe JW, Gueorguieva R, Tolin DF. Improvements in emotion regulation following repetitive transcranial magnetic stimulation for generalized anxiety disorder. *J Anxiety Disord* 2016; **43:** 1-7.

*Original article:* Diefenbach GJ, Bragdon LB, Zertuche L, Hyatt CJ, Hallion LS, Tolin DF *et al.* Repetitive transcranial magnetic stimulation for generalised anxiety disorder: a pilot randomised, double-blind, sham-controlled trial. *Br J Psychiatry* 2016; **209**(3)**:** 222-228.

14. Diefenbach GJ, Rabany L, Hallion LS, Tolin DF, Goethe JW, Gueorguieva R *et al.* Sleep improvements and associations with default mode network functional connectivity following rTMS for generalized anxiety disorder. *Brain Stimulation* 2019; **12**(1)**:** 184-186.

*Original article:* Diefenbach GJ, Bragdon LB, Zertuche L, Hyatt CJ, Hallion LS, Tolin DF *et al.* Repetitive transcranial magnetic stimulation for generalised anxiety disorder: a pilot randomised, double-blind, sham-controlled trial. *Br J Psychiatry* 2016; **209**(3)**:** 222-228.

15. Dlabac-de Lange JJ, Liemburg EJ, Bais L, van de Poel-Mustafayeva AT, de Lange-de Klerk ESM, Knegtering H *et al.* Effect of Bilateral Prefrontal rTMS on Left Prefrontal NAA and Glx Levels in Schizophrenia Patients with Predominant Negative Symptoms: An Exploratory Study. *Brain Stimul* 2017; **10**(1)**:** 59-64.

*Original article:* Dlabac-de Lange JJ, Bais L, van Es FD, Visser BG, Reinink E, Bakker B *et al.* Efficacy of bilateral repetitive transcranial magnetic stimulation for negative symptoms of schizophrenia: results of a multicenter double-blind randomized controlled trial. *Psychol Med* 2015; **45**(6)**:** 1263-1275.

16. Duprat R, Wu GR, De Raedt R, Baeken C. Accelerated iTBS treatment in depressed patients differentially modulates reward system activity based on anhedonia. *World Journal of Biological Psychiatry* 2018; **19**(7)**:** 497-508.

*Original article:* Duprat R, Desmyter S, De Raedt R, van Heeringen K, Van den Abbeele D, Tandt H *et al.* Accelerated intermittent theta burst stimulation treatment in medication-resistant major depression: A fast road to remission? *Journal of Affective Disorders* 2016; **200:** 6-14.

17. Fan J, Tso IF, Maixner DF, Abagis T, Hernandez-Garcia L, Taylor SF. Segregation of salience network predicts treatment response of depression to repetitive transcranial magnetic stimulation. *Neuroimage Clin* 2019; **22:** 101719.

*Original article:* Taylor SF, Ho SS, Abagis T, Angstadt M, Maixner DF, Welsh RC *et al.* Changes in brain connectivity during a sham-controlled, transcranial magnetic stimulation trial for depression. *J Affect Disord* 2018; **232:** 143-151.

18. Fitzgerald PB, Brown TL, Marston NA, Daskalakis ZJ, de Castella A, Bradshaw JL *et al.* Motor cortical excitability and clinical response to rTMS in depression. *Journal of Affective Disorders* 2004; **82**(1)**:** 71-76.

*Original article:* Fitzgerald PB, Brown TL, Marston NA, Daskalakis ZJ, De Castella A, Kulkarni J. Transcranial magnetic stimulation in the treatment of depression: a double-blind, placebo-controlled trial. *Arch Gen Psychiatry* 2003; **60**(10)**:** 1002-1008.

19. Giacobbe P, Mithani K, Meng Y, Vila-Rodriguez F, Daskalakis ZJ, Downar J *et al.* Evaluation of the effects of rTMS on self-reported quality of life and disability in treatment-resistant depression: A THREE-D study. *Journal of Affective Disorders* 2020; **268**(pp 127-133).

*Original article:* Blumberger DM, Vila-Rodriguez F, Thorpe KE, Feffer K, Noda Y, Giacobbe P *et al.* Effectiveness of theta burst versus high-frequency repetitive transcranial magnetic stimulation in patients with depression (THREE-D): a randomised non-inferiority trial. *Lancet* 2018; **391**(10131)**:** 1683-1692.

20. Goerigk S, Cretaz E, Sampaio-Junior B, Vieira É LM, Gattaz W, Klein I *et al.* Effects of tDCS on neuroplasticity and inflammatory biomarkers in bipolar depression: Results from a sham-controlled study. *Prog Neuropsychopharmacol Biol Psychiatry* 2020; **105:** 110119.

*Original article:* Sampaio B, Tortella G, Borrione L, Moffa AH, Machado-Vieira R, Cretaz E *et al.* Efficacy and Safety of Transcranial Direct Current Stimulation as an Add-on Treatment for Bipolar Depression A Randomized Clinical Trial. *Jama Psychiatry* 2018; **75**(2)**:** 158-166.

21. Hansbauer M, Wobrock T, Kunze B, Langguth B, Landgrebe M, Eichhammer P *et al.* Efficacy of high-frequency repetitive transcranial magnetic stimulation on PANSS factors in schizophrenia with predominant negative symptoms - Results from an exploratory re-analysis. *Psychiatry Res* 2018; **263:** 22-29.

*Original article:* Wobrock T, Guse B, Cordes J, Wölwer W, Winterer G, Gaebel W *et al.* Left prefrontal high-frequency repetitive transcranial magnetic stimulation for the treatment of schizophrenia with predominant negative symptoms: a sham-controlled, randomized multicenter trial. *Biol Psychiatry* 2015; **77**(11)**:** 979-988.

22. Hasan A, Wobrock T, Guse B, Langguth B, Landgrebe M, Eichhammer P *et al.* Structural brain changes are associated with response of negative symptoms to prefrontal repetitive transcranial magnetic stimulation in patients with schizophrenia. *Mol Psychiatry* 2017; **22**(6)**:** 857-864.

*Original article:* Wobrock T, Guse B, Cordes J, Wölwer W, Winterer G, Gaebel W *et al.* Left prefrontal high-frequency repetitive transcranial magnetic stimulation for the treatment of schizophrenia with predominant negative symptoms: a sham-controlled, randomized multicenter trial. *Biol Psychiatry* 2015; **77**(11)**:** 979-988.

23. Herwig U, Cardenas-Morales L, Connemann BJ, Kammer T, Schonfeldt-Lecuona C. Sham or real-Post hoc estimation of stimulation condition in a randomized transcranial magnetic stimulation trial. *Neurosci Lett* 2010; **471**(1)**:** 30-33.

*Original article:* Herwig U, Fallgatter AJ, Höppner J, Eschweiler GW, Kron M, Hajak G *et al.* Antidepressant effects of augmentative transcranial magnetic stimulation: randomised multicentre trial. *Br J Psychiatry* 2007; **191:** 441-448.

24. Hsu JH, Downar J, Vila-Rodriguez F, Daskalakis ZJ, Blumberger DM. Impact of prior treatment on remission with intermittent theta burst versus high-frequency repetitive transcranial magnetic stimulation in treatment resistant depression. *Brain Stimulation* 2019; **12**(6)**:** 1553-1555.

*Original article:* Blumberger DM, Vila-Rodriguez F, Thorpe KE, Feffer K, Noda Y, Giacobbe P *et al.* Effectiveness of theta burst versus high-frequency repetitive transcranial magnetic stimulation in patients with depression (THREE-D): a randomised non-inferiority trial. *Lancet* 2018; **391**(10131)**:** 1683-1692.

25. Huber TJ, Schneider U, Rollnik J. Gender differences in the effect of repetitive transcranial magnetic stimulation in schizophrenia. *Psychiatry Research* 2003; **120**(1)**:** 103-105.

*Original article:* Rollnik JD, Huber TJ, Mogk H, Siggelkow S, Kropp S, Dengler R *et al.* High frequency repetitive transcranial magnetic stimulation (rTMS) of the dorsolateral prefrontal cortex in schizophrenic patients. *Neuroreport* 2000; **11**(18)**:** 4013-4015.

26. Janicak PG, Nahas Z, Lisanby SH, Solvason HB, Sampson SM, McDonald WM *et al.* Durability of clinical benefit with transcranial magnetic stimulation (TMS) in the treatment of pharmacoresistant major depression: assessment of relapse during a 6-month, multisite, open-label study. *Brain Stimulation* 2010; **3**(4)**:** 187-199.

*Original article:* O'Reardon JP, Solvason HB, Janicak PG, Sampson S, Isenberg KE, Nahas Z *et al.* Efficacy and safety of transcranial magnetic stimulation in the acute treatment of major depression: A multisite randomized controlled trial. *Biological Psychiatry* 2007; **62**(11)**:** 1208-1216.

27. Johnson KA, Baig M, Ramsey D, Lisanby SH, Avery D, McDonald WM *et al.* Prefrontal rTMS for treating depression: location and intensity results from the OPT-TMS multi-site clinical trial. *Brain Stimulation* 2013; **6**(2)**:** 108-117.

*Original article:* George MS, Lisanby SH, Avery D, McDonald WM, Durkalski V, Pavlicova M *et al.* Daily left prefrontal transcranial magnetic stimulation therapy for major depressive disorder: A sham-controlled randomized trial. *Archives of General Psychiatry* 2010; **67**(5)**:** 507-516.

28. Kambeitz J, Goerigk S, Gattaz W, Falkai P, Bensenor IM, Lotufo PA *et al.* Clinical patterns differentially predict response to transcranial direct current stimulation (tDCS) and escitalopram in major depression: A machine learning analysis of the ELECT-TDCS study. *Journal of Affective Disorders* 2020; **265**(pp 460-467).

*Original article:* Brunoni AR, Moffa AH, Sampaio-Junior B, Borrione L, Moreno ML, Fernandes RA *et al.* Trial of Electrical Direct-Current Therapy versus Escitalopram for Depression. *N Engl J Med* 2017; **376**(26)**:** 2523-2533.

29. Kamp D, Brinkmeyer J, Agelink MW, Habakuck M, Mobascher A, Wölwer W *et al.* High frequency repetitive transcranial magnetic stimulation (rTMS) reduces EEG-hypofrontality in patients with schizophrenia. *Psychiatry Res* 2016; **236:** 199-201.

*Original article:* Cordes J, Thünker J, Agelink MW, Arends M, Mobascher A, Wobrock T *et al.* Effects of 10 Hz repetitive transcranial magnetic stimulation (rTMS) on clinical global impression in chronic schizophrenia. *Psychiatry Res* 2010; **177**(1-2)**:** 32-36.

30. Kamp D, Engelke C, Wobrock T, Kunze B, Wolwer W, Winterer G *et al.* Letter to the Editor: Influence of rTMS on smoking in patients with schizophrenia. *Schizophrenia Research* 2018; **192:** 481-484.

*Original article:* Wobrock T, Guse B, Cordes J, Wölwer W, Winterer G, Gaebel W *et al.* Left prefrontal high-frequency repetitive transcranial magnetic stimulation for the treatment of schizophrenia with predominant negative symptoms: a sham-controlled, randomized multicenter trial. *Biol Psychiatry* 2015; **77**(11)**:** 979-988.

31. Kamp D, Engelke C, Wobrock T, Wölwer W, Winterer G, Schmidt-Kraepelin C *et al.* Left prefrontal high-frequency rTMS may improve movement disorder in schizophrenia patients with predominant negative symptoms - A secondary analysis of a sham-controlled, randomized multicenter trial. *Schizophr Res* 2019; **204:** 445-447.

*Original article:* Wobrock T, Guse B, Cordes J, Wölwer W, Winterer G, Gaebel W *et al.* Left prefrontal high-frequency repetitive transcranial magnetic stimulation for the treatment of schizophrenia with predominant negative symptoms: a sham-controlled, randomized multicenter trial. *Biol Psychiatry* 2015; **77**(11)**:** 979-988.

32. Kao YC, Tzeng NS, Chao CY, Chang CC, Chang HA. Modulation of self-appraisal of illness, medication adherence, life quality and autonomic functioning by transcranial direct current stimulation in schizophrenia patients. *Clin Neurophysiol* 2020; **131**(8)**:** 1997-2007.

*Original article:* Chang C-C, Kao Y-C, Chao C-Y, Chang H-A. Enhancement of cognitive insight and higher-order neurocognitive function by fronto-temporal transcranial direct current stimulation (tDCS) in patients with schizophrenia. *Schizophrenia Research* 2019; **208:** 430-438.

33. Kaster TS, Downar J, Vila-Rodriguez F, Thorpe KE, Feffer K, Noda Y *et al.* Trajectories of Response to Dorsolateral Prefrontal rTMS in Major Depression: A THREE-D Study. *Am J Psychiatry* 2019; **176**(5)**:** 367-375.

*Original article:* Blumberger DM, Vila-Rodriguez F, Thorpe KE, Feffer K, Noda Y, Giacobbe P *et al.* Effectiveness of theta burst versus high-frequency repetitive transcranial magnetic stimulation in patients with depression (THREE-D): a randomised non-inferiority trial. *Lancet* 2018; **391**(10131)**:** 1683-1692.

34. Koutsouleris N, Wobrock T, Guse B, Langguth B, Landgrebe M, Eichhammer P *et al.* Predicting Response to Repetitive Transcranial Magnetic Stimulation in Patients With Schizophrenia Using Structural Magnetic Resonance Imaging: A Multisite Machine Learning Analysis. *Schizophr Bull* 2018; **44**(5)**:** 1021-1034.

*Original article:* Wobrock T, Guse B, Cordes J, Wölwer W, Winterer G, Gaebel W *et al.* Left prefrontal high-frequency repetitive transcranial magnetic stimulation for the treatment of schizophrenia with predominant negative symptoms: a sham-controlled, randomized multicenter trial. *Biol Psychiatry* 2015; **77**(11)**:** 979-988.

35. Kozel FA, Johnson KA, Nahas Z, Nakonezny PA, Morgan PS, Anderson BS *et al.* Fractional anisotropy changes after several weeks of daily left high-frequency repetitive transcranial magnetic stimulation of the prefrontal cortex to treat major depression. *Journal of ECT* 2011; **27**(1)**:** 5-10.

*Original article:* O'Reardon JP, Solvason HB, Janicak PG, Sampson S, Isenberg KE, Nahas Z *et al.* Efficacy and safety of transcranial magnetic stimulation in the acute treatment of major depression: A multisite randomized controlled trial. *Biological Psychiatry* 2007; **62**(11)**:** 1208-1216.

36. Liemburg EJ, Dlabac-De Lange JJ, Bais L, Knegtering H, Aleman A. Effects of bilateral prefrontal rTMS on brain activation during social-emotional evaluation in schizophrenia: A double-blind, randomized, exploratory study. *Schizophr Res* 2018; **202:** 210-211.

*Original article:* Dlabac-de Lange JJ, Liemburg EJ, Bais L, Renken RJ, Knegtering H, Aleman A. Effect of rTMS on brain activation in schizophrenia with negative symptoms: A proof-of-principle study. *Schizophr Res* 2015; **168**(1-2)**:** 475-482.

37. Light SN, Bieliauskas LA, Taylor SF. Measuring change in anhedonia using the "Happy Faces" task pre- to post-repetitive transcranial magnetic stimulation (rTMS) treatment to left dorsolateral prefrontal cortex in Major Depressive Disorder (MDD): relation to empathic happiness. *Transl Psychiatry* 2019; **9**(1)**:** 217.

*Original article:* Taylor SF, Ho SS, Abagis T, Angstadt M, Maixner DF, Welsh RC *et al.* Changes in brain connectivity during a sham-controlled, transcranial magnetic stimulation trial for depression. *J Affect Disord* 2018; **232:** 143-151.

38. Lisanby SH, Husain MM, Rosenquist PB, Maixner D, Gutierrez R, Krystal A *et al.* Daily left prefrontal repetitive transcranial magnetic stimulation in the acute treatment of major depression: clinical predictors of outcome in a multisite, randomized controlled clinical trial. *Neuropsychopharmacology* 2009; **34**(2)**:** 522-534.

*Original article:* O'Reardon JP, Solvason HB, Janicak PG, Sampson S, Isenberg KE, Nahas Z *et al.* Efficacy and safety of transcranial magnetic stimulation in the acute treatment of major depression: A multisite randomized controlled trial. *Biological Psychiatry* 2007; **62**(11)**:** 1208-1216.

39. Malm E, Struckmann W, Persson J, Bodén R. Pain trajectories of dorsomedial prefrontal intermittent theta burst stimulation versus sham treatment in depression. *BMC Neurol* 2020; **20**(1)**:** 311.

*Original article:* Struckmann W, Persson J, Weigl W, Gingnell M, Boden R. Modulation of the prefrontal blood oxygenation response to intermittent theta-burst stimulation in depression: A sham-controlled study with functional near-infrared spectroscopy. *World Journal of Biological Psychiatry* 2020.

40. Mantovani A, Rossi S, Bassi BD, Simpson HB, Fallon BA, Lisanby SH. Modulation of motor cortex excitability in obsessive-compulsive disorder: an exploratory study on the relations of neurophysiology measures with clinical outcome. *Psychiatry Res* 2013; **210**(3)**:** 1026-1032.

*Original article:* Mantovani A, Simpson HB, Fallon BA, Rossi S, Lisanby SH. Randomized sham-controlled trial of repetitive transcranial magnetic stimulation in treatment-resistant obsessive-compulsive disorder. *Int J Neuropsychopharmacol* 2010; **13**(2)**:** 217-227.

41. Martin DM, McClintock SM, Aaronson ST, Alonzo A, Husain MM, Lisanby SH *et al.* Pre-treatment attentional processing speed and antidepressant response to transcranial direct current stimulation: Results from an international randomized controlled trial. *Brain Stimul* 2018; **11**(6)**:** 1282-1290.

*Original article:* Loo CK, Husain MM, McDonald WM, Aaronson S, O'Reardon JP, Alonzo A *et al.* International randomized-controlled trial of transcranial Direct Current Stimulation in depression. *Brain Stimulation* 2018; **11**(1)**:** 125-133.

42. Martinot M-LP, Martinot J-L, Ringuenet D, Galinowski A, Gallarda T, Bellivier F *et al.* Baseline brain metabolism in resistant depression and response to transcranial magnetic stimulation. *Neuropsychopharmacology* 2011; **36**(13)**:** 2710-2719.

*Original article:* Paillere Martinot ML, Galinowski A, Ringuenet D, Gallarda T, Bellivier F, Lefaucheur JP *et al.* Response to rTMS in resistant depressed patients, and baseline fluorodeoxyglucose-PET in prefrontal and limbic regions. *Biological Psychiatry* 2009; **Conference:** 64th Annual Scientific Convention and Meeting of the Society of Biological Psychiatry. Vancouver.

43. Palm U, Fintescu Z, Obermeier M, Schiller C, Reisinger E, Keeser D *et al.* Serum levels of brain-derived neurotrophic factor are unchanged after transcranial direct current stimulation in treatment-resistant depression. *J Affect Disord* 2013; **150**(2)**:** 659-663.

*Original article:* Palm U, Schiller C, Fintescu Z, Obermeier M, Keeser D, Reisinger E *et al.* Transcranial direct current stimulation in treatment resistant depression: a randomized double-blind, placebo-controlled study. *Brain Stimul* 2012; **5**(3)**:** 242-251.

44. Persson J, Struckmann W, Gingnell M, Fällmar D, Bodén R. Intermittent theta burst stimulation over the dorsomedial prefrontal cortex modulates resting-state connectivity in depressive patients: A sham-controlled study. *Behav Brain Res* 2020; **394:** 112834.

*Original article:* Struckmann W, Persson J, Weigl W, Gingnell M, Boden R. Modulation of the prefrontal blood oxygenation response to intermittent theta-burst stimulation in depression: A sham-controlled study with functional near-infrared spectroscopy. *World Journal of Biological Psychiatry* 2020.

45. Poulet E, Galvao F, Haffen E, Szekely D, Brault C, Haesebaert F *et al.* Effects of smoking status and MADRS retardation factor on response to low frequency repetitive transcranial magnetic stimulation for depression. *European Psychiatry* 2016; **38**(pp 40-44).

*Original article:* Brunelin J, Mondino M, Gassab L, Haesebaert F, Gaha L, Suaud-Chagny MF *et al.* Examining transcranial direct-current stimulation (tDCS) as a treatment for hallucinations in schizophrenia. *Am J Psychiatry* 2012; **169**(7)**:** 719-724.

46. Rigonatti SP, Boggio PS, Myczkowski ML, Otta E, Fiquer JT, Ribeiro RB *et al.* Transcranial direct stimulation and fluoxetine for the treatment of depression. *European Psychiatry* 2008; **23**(1)**:** 74-76.

*Original article:* Boggio PS, Rigonatti SP, Ribeiro RB, Myczkowski ML, Nitsche MA, Pascual-Leone A *et al.* A randomized, double-blind clinical trial on the efficacy of cortical direct current stimulation for the treatment of major depression. *Int J Neuropsychopharmacol* 2008; **11**(2)**:** 249-254.

47. Schutter DJ, van Honk J, Laman M, Vergouwen AC, Koerselman F. Increased sensitivity for angry faces in depressive disorder following 2 weeks of 2-Hz repetitive transcranial magnetic stimulation to the right parietal cortex. *International Journal of Neuropsychopharmacology* 2010; **13**(9)**:** 1155-1161.

*Original article:* Schutter DJ, Laman DM, van Honk J, Vergouwen AC, Koerselman GF. Partial clinical response to 2 weeks of 2 Hz repetitive transcranial magnetic stimulation to the right parietal cortex in depression. *International Journal of Neuropsychopharmacology* 2009; **12**(5)**:** 643-650.

48. Tastevin M, Richieri R, Boyer L, Fond G, Lancon C, Guedj E. Brain PET metabolic substrate of TMS response in pharmaco-resistant depression. *Brain Stimulation* 2020; **13**(3)**:** 683-685.

*Original article:* Tastevin M, Baumstarck K, Groppi F, Cermolacce M, Lagrange G, Lançon C *et al.* Double cone coil rTMS efficacy for treatment-resistant depression: A prospective randomized controlled trial. *Brain Stimul* 2020; **13**(1)**:** 256-258.

49. Trevizol AP, Downar J, Vila-Rodriguez F, Konstantinou G, Daskalakis ZJ, Blumberger DM. Effect of repetitive transcranial magnetic stimulation on anxiety symptoms in patients with major depression: An analysis from the THREE-D trial. *Depress Anxiety* 2021; **38**(3)**:** 262-271.

*Original article:* Blumberger D, Tran L, Fitzgerald P, Hoy KB, Daskalakis ZJ. A randomized double-blind sham-controlled study of transcranial direct current stimulation for treatment-resistant major depression. *Front Psychiatry* 2012; **3:** 74.

50. Trevizol AP, Downar J, Vila-Rodriguez F, Thorpe KE, Daskalakis ZJ, Blumberger DM. Predictors of remission after repetitive transcranial magnetic stimulation for the treatment of major depressive disorder: An analysis from the randomised non-inferiority THREE-D trial. *EClinicalMedicine* 2020; **22:** 100349.

*Original article:* Blumberger DM, Vila-Rodriguez F, Thorpe KE, Feffer K, Noda Y, Giacobbe P *et al.* Effectiveness of theta burst versus high-frequency repetitive transcranial magnetic stimulation in patients with depression (THREE-D): a randomised non-inferiority trial. *Lancet* 2018; **391**(10131)**:** 1683-1692.

51. Trevizol AP, Goldberger KW, Mulsant BH, Rajji TK, Downar J, Daskalakis ZJ *et al.* Unilateral and bilateral repetitive transcranial magnetic stimulation for treatment-resistant late-life depression. *Int J Geriatr Psychiatry* 2019; **34**(6)**:** 822-827.

*Original article:* Blumberger DM, Vila-Rodriguez F, Thorpe KE, Feffer K, Noda Y, Giacobbe P *et al.* Effectiveness of theta burst versus high-frequency repetitive transcranial magnetic stimulation in patients with depression (THREE-D): a randomised non-inferiority trial. *Lancet* 2018; **391**(10131)**:** 1683-1692.

52. Wagner E, Wobrock T, Kunze B, Langguth B, Landgrebe M, Eichhammer P *et al.* Efficacy of high-frequency repetitive transcranial magnetic stimulation in schizophrenia patients with treatment-resistant negative symptoms treated with clozapine. *Schizophr Res* 2019; **208:** 370-376.

*Original article:* Wobrock T, Guse B, Cordes J, Wölwer W, Winterer G, Gaebel W *et al.* Left prefrontal high-frequency repetitive transcranial magnetic stimulation for the treatment of schizophrenia with predominant negative symptoms: a sham-controlled, randomized multicenter trial. *Biol Psychiatry* 2015; **77**(11)**:** 979-988.

53. Widge AS, Avery DH, Zarkowski P. Baseline and treatment-emergent EEG biomarkers of antidepressant medication response do not predict response to repetitive transcranial magnetic stimulation. *Brain Stimulation* 2013; **6**(6)**:** 929-931.

*Original article:* George MS, Lisanby SH, Avery D, McDonald WM, Durkalski V, Pavlicova M *et al.* Daily left prefrontal transcranial magnetic stimulation therapy for major depressive disorder: A sham-controlled randomized trial. *Archives of General Psychiatry* 2010; **67**(5)**:** 507-516.

54. Wölwer W, Lowe A, Brinkmeyer J, Streit M, Habakuck M, Agelink MW *et al.* Repetitive transcranial magnetic stimulation (rTMS) improves facial affect recognition in schizophrenia. *Brain Stimul* 2014; **7**(4)**:** 559-563.

*Original article:* Mittrach M, Thünker J, Winterer G, Agelink MW, Regenbrecht G, Arends M *et al.* The tolerability of rTMS treatment in schizophrenia with respect to cognitive function. *Pharmacopsychiatry* 2010; **43**(3)**:** 110-117.

55. Wu G-R, Wang X, Baeken C. Baseline functional connectivity may predict placebo responses to accelerated rtms treatment in major depression. *Human Brain Mapping* 2020; **41**(3)**:** 632-639.

*Original article:* Desmyter S, Duprat R, Baeken C, Van Autreve S, Audenaert K, van Heeringen K. Accelerated Intermittent Theta Burst Stimulation for Suicide Risk in Therapy-Resistant Depressed Patients: A Randomized, Sham-Controlled Trial. *Front Hum Neurosci* 2016; **10:** 480.

56. Wu W, Zhang Y, Jiang J, Lucas MV, Fonzo GA, Rolle CE *et al.* An electroencephalographic signature predicts antidepressant response in major depression. *Nature Biotechnology* 2020; **38**(4)**:** 439-447.

*Original article:* Desmyter S, Duprat R, Baeken C, Van Autreve S, Audenaert K, van Heeringen K. Accelerated Intermittent Theta Burst Stimulation for Suicide Risk in Therapy-Resistant Depressed Patients: A Randomized, Sham-Controlled Trial. *Front Hum Neurosci* 2016; **10:** 480.

57. Zanão TA, Moffa AH, Shiozawa P, Lotufo PA, Benseñor IM, Brunoni AR. Impact of two or less missing treatment sessions on tDCS clinical efficacy: results from a factorial, randomized, controlled trial in major depression. *Neuromodulation* 2014; **17**(8)**:** 737-742; discussion 742.

*Original article:* Brunoni AR, Valiengo L, Baccaro A, Zanão TA, de Oliveira JF, Goulart A *et al.* The sertraline vs. electrical current therapy for treating depression clinical study: results from a factorial, randomized, controlled trial. *JAMA Psychiatry* 2013; **70**(4)**:** 383-391.

58. Zheng H, Jia F, Guo G, Quan D, Li G, Wu H *et al.* Abnormal Anterior Cingulate N-Acetylaspartate and Executive Functioning in Treatment-Resistant Depression After rTMS Therapy. *Int J Neuropsychopharmacol* 2015; **18**(11)**:** pyv059.

*Original article:* Zheng HR, Zhang L, Li LJ, Liu P, Gao JL, Liu XY *et al.* High-frequency rTMS treatment increases left prefrontal myo-inositol in young patients with treatment-resistant depression. *Progress in Neuro-Psychopharmacology & Biological Psychiatry* 2010; **34**(7)**:** 1189-1195.

**Supplementary material D.** Changes from pre-registered protocol and post-hoc analyses

Our study was registered with PROSPERO (number CRD42021250057).

**Exclusion criteria**

During study selection, we noticed that several trials co-initiated another treatment with neurostimulation. We agreed that protocols adopting this approach could not accurately be compared with those using neurostimulation as a monotherapy or augmentation of a stable treatment regimen. Thus, we added to our exclusion criteria the co-initiation of another therapy with neurostimulation.

**Additional outcomes**

During data extraction, we noticed that several included eligible trials also included a measure of processing speed. Thus, we also included pre- and post-treatment processing speed performance on standardized task in our data extraction and analyses.

**Measures of effect**

In our protocol submission, we stated that our measure of effect was standardized mean differences. During data extraction, we found that several trials did not report continuous outcomes. For eligible RCTs without continuous outcome data, we also calculated odds ratios from response rate data.

**Pairwise meta-analyses**

In our protocol submission, we stated that we planned to conduct pairwise meta-analyses for each psychiatric disorder where 3 or more eligible RCTs were available. Given the paucity of studies for certain outcomes, we agreed that conducting analyses for each psychiatric disorder and subgroup with at least 2 eligible RCTs was appropriate.

**Meta-regression analyses**

In our protocol submission, we stated that we planned to run meta-regression analyses for study quality, population, and stimulation parameters. During data synthesis, we agreed that sensitivity analyses and subgroup analyses were sufficient to investigate the effects of study quality on overall results, and to explore heterogeneity. Regarding study populations, we did collect data for age, however trials including children or adolescents were scarce and there were no eligible trials including only children or adolescents.

**Subgroup analyses**

In our protocol submission, we planned to run subgroup analyses for stimulation type in both TMS and tDCS. As there were fewer than 10 tDCS trials for any mental disorder, and little variation in electrode placement, we decided to only include subgroup analyses for mental disorders with more than 10 TMS trials.

**Supplementary Table 1.** Study characteristics of included randomized controlled trials

| **Study** | **Included study arms** | **Total study arms** | **Arm** | | **N** | **Age (SD)** | | **Cortical target** | | **TR** | **Hz/mA (% MT)** | | **Treatment strategy** | **N session (weeks)** | **Included scales/ tasks** | **Source of outcome data** |
| --- | --- | --- | --- | --- | --- | --- | --- | --- | --- | --- | --- | --- | --- | --- | --- | --- |
| **ADHD** |  |  |  | |  |  | |  | |  |  | |  |  |  |  |
| Alyagon, et al. (2020) | 2 | 2 | rTMS | | 20 | 26.62 (2.56) | | RDLPFC & VLPFC | | No | 18 Hz (120%) | | Monotherapy | 15 (3w) | CAARS | Original article |
|  |  |  | Sham | | 16 | 27.64 (5.91) | |  | |  |  | |  |  |  |  |
| Paz, et al. (2018) | 2 | 2 | dTMS | | 9 | 32.11 (6.47) | | BLPFC | | No | 18 Hz (120%) | | Monotherapy | 20 (4w) | CAARS | Original article |
|  |  |  | Sham | | 13 | 30.85 (6.82) | |  | |  |  | |  |  |  |  |
| **Depression** | |  |  | |  |  | |  | |  |  | |  |  |  |  |
| Anderson, et al. (2007) | 2 | 2 | rTMS | | 13 | 48 (8) | | LDLPFC | | Mixed | 10 Hz (110%) | | Mixed | 12 (4-6w) | MADRS | Previous analysis^5^ |
|  |  |  | Sham | | 16 | 46 (12) | |  | |  |  | |  |  |  |  |
| Avery, et al. (1999) | 2 | 2 | rTMS | | 4 | 44.3 (10.1) | | LDLPFC | | Yes | 10 Hz (80%) | | Mixed | 10 (3w) | HDRS-21 | Previous analysis^5^ |
|  |  |  | Sham | | 2 | 45 (7.1) | |  | |  |  | |  |  |  |  |
| Avery, et al. (2006) | 2 | 2 | rTMS | | 35 | 44.3 (10.3) | | LDLPFC | | Yes | 10 Hz (110%) | | Mixed | 15 (4w) | HDRS-17 | Previous analysis^5^ |
|  |  |  | Sham | | 33 | 44.2 (9.7) | |  | |  |  | |  |  |  |  |
| Baeken, et al. (2013) | 2 | 2 | rTMS | | 10 | 51.8 (12.1) | | LDLPFC | | Yes | 20 Hz (110%) | | Monotherapy | 20 (1w) | HDRS-17 | Previous analysis^5^ |
|  |  |  | Sham | | 11 | 47.3 (13.7) | |  | |  |  | |  |  |  |  |
| Bakim, et al. (2012) | 3^1^ | 3 | rTMS | | 23 | 40.8 (9) | | LDLPFC | | Yes | 20 Hz (80%, 110%) | | Augmentation | 30 (6w) | HDRS-17 | Previous analysis^5^ |
|  |  |  | Sham | | 12 | 44.4 (10.2) | |  | |  |  | |  |  |  |  |
| Berman, et al. (2000) | 2 | 2 | rTMS | | 10 | 45.2 (9.5) | | LDLPFC | | Yes | 20 Hz (80%) | | Monotherapy | 10 (2w) | MADRS | Previous analysis^5^ |
|  |  |  | Sham | | 10 | 39.4 (10.8) | |  | |  |  | |  |  |  |  |
| Beynel, et al. (2014) | 2 | 2 | iTBS | | 5 | 55 (12.8) | | LDLPFC | | Yes | TBS (80%) | | Augmentation | 10-30 (1-3w) | MADRS | Previous analysis^5^ |
|  |  |  | Sham | | 7 | 47.4 (10.9) | |  | |  |  | |  |  |  |  |
| Blumberger, et al. (2012)A | 3 | 3 | rTMS | | 28 | 58 (12.5) | | BLDLPFC | | Yes | 1 Hz R, 10 Hz L (100%, 120%) | | Mixed | 15 (3w) | HDRS-17 | Previous analysis^5^ |
|  |  |  | rTMS | | 24 | 48.9 (13.4) | | LDLPFC | |  | 10 Hz (100%, 120%) | |  | 15 (3w) |  |  |
|  |  |  | Sham | | 22 | 45.8 (13.4) | |  | |  |  | |  |  |  |  |
| Blumberger, et al. (2012)B | 2 | 2 | tDCS | | 13 | 45.3 (11.6) | | Anode: LDLPFC Cathode: RDLPFC | | Yes | 2mA | | Mixed | 7 (3w) | HDRS-17 | Previous analysis^5^ |
|  |  |  | Sham | | 11 | 49.7 (9.4) | |  | |  |  | |  |  |  |  |
| Blumberger, et al. (2016) | 3 | 3 | rTMS | | 40 | 46.5 (14.1) | | LDLPFC | | Yes | 10 Hz (120%) | | Augmentation | 15 (3w) | HDRS-17 | Original article |
|  |  |  | rTMS | | 40 | 46.4 (12.5) | | BLDLPFC | |  | 1 Hz R, 10 Hz L (120%) | |  | 15 (3w) |  |  |
|  |  |  | Sham | | 41 | 48.1 (12) | |  | |  |  | |  |  |  |  |
| Bortolomasi, et al. (2007) | 2 | 2 | rTMS | | 12 | 55.6 (15.4) | | LDLPFC | | Yes | 20 Hz (90%) | | Mixed | 5 (1w) | HDRS-24 | Previous analysis^5^ |
|  |  |  | Sham | | 7 |  |  |  | |  |  | |  |  |  |  |
| Boutros, et al. (2002) | 2 | 2 | rTMS | | 12 | 49.5 (8) | | LDLPFC | | Yes | 20 Hz (80%) | | Mixed | 10 (2w) | HDRS-25 | Previous analysis^5^ |
|  |  |  | Sham | | 10 | 52 (7) | |  | |  |  | |  |  |  |  |
| Bulteau, et al. (2019) | 2 | 2 | iTBS | | 12 | 52.7 (10.8) | | LDLPFC | | Yes | TBS (80%) | | Augmentation | 30 (3w) | MADRS | Original article |
|  |  |  | Sham | | 14 | 53.1 (12.5) | |  | |  |  | |  |  |  |  |
| Carpenter, et al. (2017) | 2 | 2 | rTMS | | 47 | 45.6 (12) | | LDLPFC and DMPFC | | Yes | 10 Hz (120%) | | Mixed | 20 (4-6w) | HDRS-24 | Original article |
|  |  |  | Sham | | 45 | 47.6 (13.1) | |  | |  |  | |  |  |  |  |
| Chen, et al. (2013) | 2 | 2 | rTMS | | 10 | 44.1 (4.4) | | LDLPFC | | Yes | 20 Hz (90%) | | Augmentation | 10 (4w) | HDRS-21 | Previous analysis^5^ |
|  |  |  | Sham | | 11 | 47.3 (3.5) | |  | |  |  | |  |  |  |  |
| Chistyakov, et al. (2015) | 2 | 2 | cTBS | | 15 | 52.7 (11.1) | | RDLPFC | | Yes | TBS (100%) | | Mixed | 10 (2w) | HDRS-21 | Previous analysis^5^ |
|  |  |  | Sham | | 14 | 50.9 (17.3) | |  | |  |  | |  |  |  |  |
| Chou, et al. (2020) | 2 | 2 | TBS | | 30 | 43.6 (16.6) | | BLDLPFC | | Yes | iTBS L cTBS R (80%) | | Monotherapy | 10 (4w) | HDRS-21 | Original article |
|  |  |  | Sham | | 30 | 42.3 (11.1) | |  | |  |  | |  |  |  |  |
| Concerto, et al. 2015 | 2 | 2 | rTMS | | 15 | 51 (6.5) | | LDLPFC | | Yes | 10 Hz (120%) | | Augmentation | 20 (4w) | HDRS-21 | Previous analysis^5^ |
|  |  |  | Sham | | 15 | 53 (6.7) | |  | |  |  | |  |  |  |  |
| Dunlop, et al. (2020) | 3 | 3 | rTMS | | 38 | 39.44 (11.62 | | DMPFC | | Yes | 20 Hz (120%) | | Augmentation | 30 (3w) | HDRS-17 | Original article |
|  |  |  | rTMS | | 41 |  |  | DMPFC | |  | 1 Hz (120%) | |  | 30 (3w) |  |  |
|  |  |  | Sham | | 41 |  |  |  | |  |  | |  |  |  |  |
| Duprat, et al. (2016) | 2 | 2 | iTBS | | 22 | 40.1 (11.5) | | LDLPFC | | Yes | TBS (110%) | | Monotherapy | 20 (2w) | HDRS-17 | Previous analysis^5^ |
|  |  |  | Sham | | 25 | 43.2 (12.2) | |  | |  |  | |  |  |  |  |
| Eschweiler, et al. (2000) | 2 | 2 | rTMS | | 5 | NR | | LDLPFC | | No | 10 Hz (90%) | | Augmentation | 5 (1w) | HDRS-21 | Previous analysis^5^ |
|  |  |  | Sham | | 5 | NR | |  | |  |  | |  |  |  |  |
| Eshel, et al. (2020) | 2 | 2 | rTMS | | 20 | 35 (6.3) | | LDLPFC | | Yes | 10 Hz (120%) | | Monotherapy | 20 (4w) | HDRS | Original article |
|  |  |  | Sham | | 13 | 37.1 (11.2) | |  | |  |  | |  |  |  |  |
| Fitzgerald, et al. (2003) | 3 | 3 | TMS | | 20 | 45.6 (11.5) | | RDLPFC | | Yes | 1 Hz (100%) | | Mixed | 10 (2w) | MADRS | Previous analysis^5^ |
|  |  |  | rTMS | | 20 | 42.2 (9.8) | | LDLPFC | |  | 10 Hz (100%) | |  | 10 (2w) |  |  |
|  |  |  | Sham | | 20 | 49.2 (14.2) | |  | |  |  | |  |  |  |  |
| Fitzgerald, et al. (2006) | 2 | 2 | rTMS | | 25 | 46.8 (10.7) | | BLDLPFC | | Yes | 1 Hz R, 10 Hz L (110%, 100%) | | Mixed | 10 (2w) | HDRS-17 | Previous analysis^5^ |
|  |  |  | Sham | | 25 | 43.7 (10.2) | |  | |  |  | |  |  |  |  |
| Fitzgerald (2008)A | 2 | 2 | TMS | | 25 | 46.8 (10.7) | | BLDLPFC | | Yes | 1 Hz R, 10 Hz L (110%, 100%) | | NA | 10 (2w) | MADRS | Original article |
|  |  |  | Sham | | 25 | 43.7 (10.2) | |  | |  |  | |  |  |  |  |
| Fitzgerald, et al. (2012) | 3 | 3 | rTMS | | 24 | 43.4 (12.7) | | LDLPFC | | Yes | 10 Hz (120%) | | Mixed | 15 (3w) | MADRS | Previous analysis^5^ |
|  |  |  | rTMS | | 22 | 40.5 (15.5) | | BLDLPFC | |  | 1Hz R 10 Hz L (120%) | |  | 15 (3w) |  |  |
|  |  |  | Sham | | 20 | 44.9 (15.7) | |  | |  |  | |  |  |  |  |
| Fitzgerald, et al. (2016) | 2 | 2 | rTMS | | 23 | 46.3 (12.6) | | BLDLPFC | | Yes | 1 Hz R, 10 Hz L (110%, 100%) | | Mixed | 20 (4w) | HDRS-17 | Previous analysis^5^ |
|  |  |  | Sham | | 23 | 49.7 (11) | |  | |  |  | |  |  |  |  |
| Fregni, et al. (2006) | 2 | 2 | tDCS | | 5 | 42.7 (10) | | Anode: LDLPFC, Cathode: RSOA | | No | 1 mA | | Monotherapy | 5 (1w) | HDRS | Original article |
|  |  |  | Sham | | 5 |  |  |  | |  |  | |  |  |  |  |
| Garcia-Toro, et al. (2001) | 2 | 2 | rTMS | | 20 | 51.5 (15.9) | | LDLPFC | | Yes | 20 Hz (90%) | | Monotherapy | 10 (2w) | HDRS-21 | Previous analysis^5^ |
|  |  |  | Sham | | 20 | 50 (11) | |  | |  |  | |  |  |  |  |
| Garcia-Toro, et al. (2006) | 3 | 3 | rTMS | | 10 | 47.2 (11.8) | | Individualised PFC | | Yes | - | | Augmentation | 10 (2w) | HDRS-21 | Original article |
|  |  |  | rTMS | | 10 | 48.5 (13.28) | | Primed LDLPFC then RDLPFC | |  | - | |  | 10 (2w) |  |  |
|  |  |  | Sham | | 10 | 51.1 (13.84) | |  | |  |  | |  |  |  |  |
| George, et al. (1997) | 2 | 2 | rTMS | | 7 | 42.4 (15.5) | | LDLPFC | | No | 20 Hz (80%) | | Mixed | 10 (2w) | HDRS-21 | Previous analysis^5^ |
|  |  |  | Sham | | 5 | 41 (8.3) | |  | |  |  | |  |  |  |  |
| George, et al. (2000) | 3^2^ | 3 | rTMS | | 22 | 42.4 (10.8) | | LDLPFC | | Mixed | 20 Hz (100%) | | Monotherapy | 10 (2w) | HDRS-21 | Previous analysis^5^ |
|  |  |  | Sham | | 10 | 48.5 (8) | |  | |  |  | |  |  |  |  |
| George, et al. (2010) | 2 | 2 | rTMS | | 92 | 47.7 (10.6) | | LDLPFC | | Yes | 10 Hz (120%) | | Monotherapy | 15 (3w) | HDRS | Previous analysis^5^ |
|  |  |  | Sham | | 98 | 46.5 (12.3) | |  | |  |  | |  |  |  |  |
| Gögler, et al. (2017) | 2 | 2 | tDCS | | 10 | 34.9 (9.37) | | Anode: LDLPFC, Cathode: RSOA | | NA | 2 mA | | Augmentation | 1 | TVA | Original analysis |
|  |  |  | Sham | | 10 | 35.8 (5.75) | |  | |  |  | |  |  |  |  |
| He, et al. (2011) | 2 | 3 | rTMS | | 55 | 37.5 (12.7) | | BLPFC | | NA | 3-15 Hz (NA) | | Monotherapy | 10 (2w) | HDRS-24 | Previous analysis^5^ |
|  |  |  | Sham | | 52 | 39 (15.2) | |  | |  |  | |  |  |  |  |
| Hernández-Ribas, et al. (2013) | 2 | 2 | rTMS | | 10 | 42.6 (5.6) | | LDLPFC | | Yes | 15 Hz (100%) | | Augmentation | 15 (3w) | HDRS-21 | Previous analysis^5^ |
|  |  |  | Sham | | 11 | 50.1 (8.1) | |  | |  |  | |  |  |  |  |
| Holtzheimer, et al. (2004) | 2 | 2 | rTMS | | 7 | 40.4 (8.5) | | LDLPFC | | Yes | 10 Hz (110%) | | Monotherapy | 10 (2w) | HDRS-17 | Previous analysis^5^ |
|  |  |  | Sham | | 8 | 45.4 (4.9) | |  | |  |  | |  |  |  |  |
| Hoppner, et al. (2003) | 3 | 3 | rTMS | | 10 | 60.4 (7.1) | | LDLPFC | | NA | 20 Hz (90%) | | Augmentation | 10 (2w) | HDRS-21 | Previous analysis^5^ |
|  |  |  | rTMS | | 10 | 52 (11.7) | | RDLPFC | |  | 1 Hz (110%) | |  | 10 (2w) |  |  |
|  |  |  | Sham | | 10 | 56.4 (13.2) | |  | |  |  | |  |  |  |  |
| Januel, et al. (2006) | 2 | 2 | rTMS | | 11 | 38.6 (11.2) | | RDLPFC | | No | 1 Hz (90%) | | Monotherapy | 16 (4w) | HDRS-17 | Previous analysis^5^ |
|  |  |  | Sham | | 16 | 37.2 (11.7) | |  | |  |  | |  |  |  |  |
| Jin and Phillips (2014) | 2 | 2 | sTMS | | 33 | 42.5 (15) | | - | | No | 8 Hz - 13 Hz (IAF) | | Augmentation | 20 (4w) | HDRS-17 | Previous analysis^5^ |
|  |  |  | Sham | | 19 | 46.3 (12.7) | |  | |  |  | |  |  |  |  |
| Kang, et al. (2016) | 2 | 2 | rTMS | | 13 | 42.8 (19.1) | | LDLPFC | | Yes | 10 Hz (120%) | | Augmentation | 10 (2w) | HDRS-17 | Previous analysis^5^ |
|  |  |  | Sham | | 11 | 52.2 (20.1) | |  | |  |  | |  |  |  |  |
| Kaster, et al. (2018) | 2 | 2 | dTMS | | 25 | 65 (5.5) | | LDLPFC | | Yes | 18 Hz (100%, 120%) | | Augmentation | 10 (4w) | HDRS-24 | Original article |
|  |  |  | Sham | | 27 | 65.4 (5.5) | |  | |  |  | |  |  |  |  |
| Kauffmann, et al. (2004) | 2 | 2 | rTMS | | 7 | 51.7 (17.2) | | RDLPFC | | Yes | 1 Hz (110%) | | Augmentation | 10 (2w) | HDRS-21 | Previous analysis^5^ |
|  |  |  | Sham | | 5 |  |  |  | |  |  | |  |  |  |  |
| Kavanaugh, et al. (2018) | 2 | 2 | rTMS | | 43 | 47.95 (12.78) | | RDLPFC | | Yes | 10 Hz (120%) | | Augmentation | 20 (4-6w) | HDRS-24, CDRS attention | Original article |
|  |  |  | Sham | | 41 | 45.86 (11.57) | |  | |  |  | |  |  |  |  |
| Kimbrell, et al. (1999) | 3 | 3 | rTMS | | 5 | 40.2 (15.1) | | LDLPFC | | Yes | 20 Hz (80%) | | Monotherapy | 10 (2w) | HDRS-21 | Previous analysis^5^ |
|  |  |  | rTMS | | 5 | 44 (15.9) | | LDLPFC | |  | 1 Hz (80%) | |  | 10 (2w) |  |  |
|  |  |  | Sham | | 5 | 43.7 (19.1) | |  | |  |  | |  |  |  |  |
| Klein, et al. (1999)A | 2 | 2 | rTMS | | 36 | 60.5 (15.1) | | RDLPFC | | No | 1 Hz (110%) | | Mixed | 10 (2w) | HDRS-17 | Previous analysis^5^ |
|  |  |  | Sham | | 34 | 58.9 (18.3) | |  | |  |  | |  |  |  |  |
| Koerselman, et al. (2004) | 2 | 2 | rTMS | | 29 | 51 (15.4) | | LDLPFC | | Mixed | 20 Hz (80%) | | Augmentation | 10 (2w) | HDRS-17 | Previous analysis^5^ |
|  |  |  | Sham | | 26 | 52 (13.2) | |  | |  |  | |  |  |  |  |
| Kreuzer, et al. (2015) | 2 | 2 | rTMS | | 15 | 46.1 (9.5) | | LDLPFC | | Mixed | 10 Hz (110%) | | Mixed | 15 (3w) | HDRS-21 | Previous analysis^5^ |
|  |  |  | Sham | | 15 | 43.8 (10.5) | |  | |  |  | |  |  |  |  |
| Lee, et al. (2018) | 2 | 2 | rTMS | | 18 | 35.89 (13.01) | | LDLPFC | | No | 10 Hz (110%) | | Mixed | 15 (3w) | HDRS-17 | Original article |
|  |  |  | Sham | | 19 | 35.89 (11.92) | |  | |  |  | |  |  |  |  |
| Lee, et al. (2019) | 3^1^ | 3 | rTMS | | 33 | 34.02 (11.31) | | LDLPFC | | No | 10 Hz (NR) | | Mixed | (15) 3w | HDRS-17 | Original article |
|  |  |  | Sham | | 14 | 36.71 (11.79) | |  | |  |  | |  |  |  |  |
| Leuchter et al. (2015) | 2 | 2 | sTMS | | 103 | 46.7 (11.2) | | - | | Mixed | 8-13 Hz (IAF) | | Monotherapy | 30 (6w) | HDRS-17 | Previous analysis^5^ |
|  |  |  | Sham | | 99 | 45.7 (12.6) | |  | |  |  | |  |  |  |  |
| Levkovitz et al. (2015) | 2 | 2 | dTMS | | 111 | 45.1 (11.7) | | LDLPFC | | Yes | 18 Hz (120%) | | Monotherapy | 20 (16w) | HDRS-21 | Previous analysis^5^ |
|  |  |  | Sham | | 122 | 47.6 (11.6) | |  | |  |  | |  |  |  |  |
| Li, et al. (2014) | 4 | 4 | iTBS | | 15 | 42.4 (NR) | | LDLPFC | | Yes | TBS (80%) | | Mixed | 10 (2w) | HDRS-17 | Previous analysis^5^ |
|  |  |  | cTBS | | 15 | 49.2 (NR) | | RDLPFC | |  | TBS (80%) | |  | 10 (2w) |  |  |
|  |  |  | iTBS + cTBS | | 15 | 42.5 (NR) | | BLDLPFC | |  | iTBS L cTBS R (80%) | |  | 10 (2w) |  |  |
|  |  |  | Sham | | 15 | 46.9 (NR) | |  | |  |  | |  |  |  |  |
| Li, et al. (2020) | 3 | 3 | piTBS | | 35 | 47.1 (14.2) | | LDLPFC | | Yes | TBS (100%) | | Monotherapy | 10 (2w) | HDRS-17 | Original article |
|  |  |  | rTMS | | 35 | 47.1 (13.8) | | LDLPFC | |  | 10 Hz (100%) | |  | 10 (2w) |  |  |
|  |  |  | Sham | | 35 | 47.1 (12.4) | |  | |  |  | |  |  |  |  |
| Lingeswaran (2011) | 2 | 2 | rTMS | | 12 | 34 (10.5) | | LDLPFC | | Mixed | 10 Hz (100%) | | Mixed | 12 (2w) | HDRS-17 | Previous analysis^5^ |
|  |  |  | Sham | | 17 | 37.2 (11.8) | |  | |  |  | |  |  |  |  |
| Loo, et al. (2001) | 2 | 2 | rTMS | | 9 | 48 (NR) | | LDLPFC | | Mixed | 10 Hz (110%) | | Mixed | 10 (2w) | TOL, digit span | Original article |
|  |  |  | Sham | | 7 |  |  |  | |  |  | |  |  |  |  |
| Loo, et al. (2003) | 2 | 2 | rTMS | | 9 | 54.9 (18) | | BLDLPFC | | Yes | 15 Hz (90%) | | Mixed | 15 (3w) | HDRS-17 | Previous analysis^5^ |
|  |  |  | Sham | | 10 | 48.4 (10.9) | |  | |  |  | |  |  |  |  |
| Loo et al. (2007) | 2 | 2 | rTMS | | 19 | 49.8 (2.5) | | LDLPFC | | Yes | 10 Hz (110%) | | Mixed | 10 (2w) | HDRS-17, TMT-A, TMT-B, digit span | Original article |
|  |  |  | Sham | | 21 | 45.7 (15) | |  | |  |  | |  |  |  |  |
| Loo, et al. (2010) | 2 | 2 | tDCS | | 20 | 49 (10) | | Anode: LDLPFC, Cathode: F8^3^ | | Mixed | 1 mA | | Mixed | 10 (2w) | HDRS-17, TMT-A, TMT-B, digit span | Original article |
|  |  |  | Sham | | 20 | 45.6 (12.5) | |  | |  |  | |  |  |  |  |
| Loo, et al. (2012) | 2 | 2 | tDCS | | 33 | 47.8 (12.5) | | Anode: LDLPFC, Cathode: F8^3^ | | Yes | 2 mA | | Mixed | 15 (3w) | MADRS, stroop interference test, digit span, | Original article |
|  |  |  | Sham | | 31 | 48.6 (12.6) | |  | |  |  | |  |  |  |  |
| Loo, et al. (2018) | 2 | 2 | tDCS | | 66 | 18-81 | | Anode: LDLPFC, Cathode: F8^3^ | | Mixed | 2.5 mA | | Mixed | 20 (4w) | MADRS | Previous analysis^5^ |
|  |  |  | Sham | | 64 |  |  |  | |  |  | |  |  |  |  |
| Manes, et al. (2001) | 2 | 2 | rTMS | | 10 | 60.5 (3.4) | | LDLPFC | | Yes | 20 Hz (80%) | | Monotherapy | 5 (1w) | HDRS-17 | Previous analysis^5^ |
|  |  |  | Sham | | 10 | 60.9 (2) | |  | |  |  | |  |  |  |  |
| Matsuda, et al. (2020) | 2 | 2 | dTMS | | 22 | 43.4 (5.5) | | LDLPFC | | NA | 18 Hz (120%) | | Augmentation | 20 (4w) | TMT-A, TMT-B | Original article |
|  |  |  | Sham | | 20 | 45.2 (7) | |  | |  |  | |  |  |  |  |
| McDonald, et al. (2006) | 3^2^ | 3 | TMS | | 50 | NR | | BLDLPFC | | Yes | 10 Hz L 1 Hz R (110%) | | Monotherapy | 10 (2w) | HDRS-21, RBANS attention | Original article |
|  |  |  | Sham | | 12 |  |  |  | |  |  | |  |  |  |  |
| McGirr, et al. (2021) | 2 | 2 | iTBS | | 19 | 43 (14.34) | | LDLPFC | | Yes | TBS (120%) | | Augmentation | 20 (4w) | MADRS | Original article |
|  |  |  | Sham | | 18 | 44.78 (13.71) | |  | |  |  | |  |  |  |  |
| Mogg, et al. (2008) | 2 | 2 | rTMS | | 29 | 55 (18) | | LDLPFC | | Yes | 10 Hz (110%) | | Mixed | 10 (2w) | HDRS-17 | Previous analysis^5^ |
|  |  |  | Sham | | 30 | 52 (15.5) | |  | |  |  | |  |  |  |  |
| Moser, et al. (2002) | 2 | 2 | rTMS | | 9 | 48-78 | | Anterior left middle frontal gyrus | | Yes | 20 Hz (80%) | | Monotherapy | 5 (NR) | TMT-A, TMT-B | Original article |
|  |  |  | Sham | | 10 |  |  |  | |  |  | |  |  |  |  |
| Mosimann, et al. (2004) | 2 | 2 | rTMS | | 15 | 60 (13.4) | | LDLPFC | | Yes | 20 Hz (100%) | | Mixed | 10 (2w) | HDRS-21 | Previous analysis^5^ |
|  |  |  | Sham | | 9 | 64.4 (13) | |  | |  |  | |  |  |  |  |
| Myczkowski, et al. (2018) | 2 | 2 | dTMS | | 20 | 40.6 (9) | | LDLPFC | | Yes | 18 Hz (120%) | | Augmentation | 20 (4w) | TMT-A, TMT-B, digit span | Previous analysis^5^ |
|  |  |  | Sham | | 23 | 41.2 (11.7) | |  | |  |  | |  |  |  |  |
| Nahas, et al. (2003) | 2 | 2 | rTMS | | 11 | 42.4 (7.3) | | LDLPFC | | No | 5 Hz (110%) | | Monotherapy | 10 (2w) | HDRS-28 | Previous analysis^5^ |
|  |  |  | Sham | | 12 | 43.3 (11.6) | |  | |  |  | |  |  |  |  |
| O'Reardon, et al. (2007) | 2 | 2 | rTMS | | 165 | 47.9 (11) | | LDLPFC | | Yes | 10 Hz (120%) | | Monotherapy | 20 (4-6w) | HDRS-17 | Previous analysis^5^ |
|  |  |  | Sham | | 160 | 48.7 (10.6) | |  | |  |  | |  |  |  |  |
| Padberg, et al. (1999) | 3 | 3 | rTMS | | 6 | 46.7 (14.7) | | LDLPFC | | Yes | 0.3 Hz (90%) | | Mixed | 5 (1w) | HDRS-21 | Original article |
|  |  |  | rTMS | | 6 | 63.5 (15.8) | | LDLPFC | |  | 10 Hz (90%) | |  | 5 (1w) |  |  |
|  |  |  | Sham | | 6 | 43.3 (11.6) | |  | |  |  | |  |  |  |  |
| Padberg, et al. (2002) | 3^1^ | 3 | rTMS | | 20 | 61.2 (13.8) | | LDLPFC | | Yes | 10 Hz (100%) | | Augmentation | 10 (2w) | HDRS-21 | Previous analysis^5^ |
|  |  |  | Sham | | 10 | 52.7 (18) | |  | |  |  | |  |  |  |  |
| Paillère-Martinot, et al. (2010) | 2 | 3 | rTMS | | 19 | 48.2 (7.8) | | LDLPFC | | Yes | 10 Hz (90%) | | Augmentation | 10 (2w) | HDRS-21 | Previous analysis^5^ |
|  |  |  | Sham | | 14 | 46.6 (10.3) | |  | |  |  | |  |  |  |  |
| Pallanti, et al. (2010) | 3 | 3 | rTMS | | 20 | 47.6 (12.2) | | RDLPFC | | Yes | 1 Hz (110%) | | Augmentation | 15 (3w) | HDRS-17 | Previous analysis^5^ |
|  |  |  | rTMS | | 20 | 51.2 (12.5) | | BLDLPFC | |  | 1 Hz R, 10 Hz L (110%, 100%) | |  | 15 (3w) |  |  |
|  |  |  | Sham | | 20 | 47.9 (9.1) | |  | |  |  | |  |  |  |  |
| Prasser, et al. (2015) | 3 | 3 | rTMS | | 18 | 50.4 (9.9) | | BLDLPFC | | Mixed | 1 Hz R, 10 Hz L (110%, 100%) | | Augmentation | 15 (3w) | HDRS-21 | Contacted authors |
|  |  |  | TBS | | 20 | 48.2 (10.9) | | BLDLPFC | |  | iTBS L cTBS R (80) | |  | 15 (3w) |  |  |
|  |  |  | Sham | | 18 | 42.6 (12.4) | |  | |  |  | |  |  |  |  |
| Rossini, et al. (2005) | 2 | 2 | rTMS | | 37 | 55.7 (10.1) | | LDLPFC | | Yes | 15 Hz (80%, 100%) | | Augmentation | 10 (2w) | HDRS-21 | Previous analysis^5^ |
|  |  |  | Sham | | 17 | 56.3 (12.6) | |  | |  |  | |  |  |  |  |
| Salehinejad, et al. (2015) | 2 | 2 | tDCS | | 15 | 28.7 (5.9) | | Anode: LDLPFC, Cathode: RDLPFC | | Yes | 2 mA | | Monotherapy | 10 (2w) | HDRS-24 | Previous analysis^5^ |
|  |  |  | Sham | | 15 | 27.9 (5.8) | |  | |  |  | |  |  |  |  |
| Salehinejad, et al. (2017) | 2 | 2 | tDCS | | 12 | 26.8 (7.1) | | Anode: LDLPFC, Cathode: RDLPFC | | Yes | 2 mA | | Monotherapy | 10 (2w) | HDRS-24 | Previous analysis^5^ |
|  |  |  | Sham | | 12 | 25.5 (4.6) | |  | |  |  | |  |  |  |  |
| Sampaio Junior, et al. (2018) | 2 | 2 | tDCS | | 30 | 46.2 (11.8) | | Anode: LDLPFC, Cathode: RDLPFC | | Mixed | 2 mA | | Augmentation | 12 (6w) | HDRS-17 | Previous analysis^5^ |
|  |  |  | Sham | | 29 | 45.7 (10.3) | |  | |  |  | |  |  |  |  |
| Schutter, et al. (2009) | 2 | 2 | rTMS | | 18 | 44.4 (11.8) | | RPL | | No | 2 Hz (90%) | | Augmentation | 10 (2w) | HDRS-17 | Original article |
|  |  |  | Sham | | 18 | 43.8 (12.5) | |  | |  |  | |  |  |  |  |
| Sharafi, et al. (2019) | 2 | 2 | tDCS | | 15 | 50.7 (10.7) | | Anode: LDLPFC, Cathode: RDLPFC | | Yes | 2 mA | | Augmentation | 10 (2w) | HDRS-17 | Original article |
|  |  |  | Sham | | 15 | 43.8 (12.6) | |  | |  |  | |  |  |  |  |
| Song, et al. (2020) | 2 | 2 | rTMS | | 28 | 31.57 (12.65) | | Visual Cortex | | No | 10 Hz (90%) | | Monotherapy | 10 (1w) | HDRS-24, TMT-A, NAB mazes | Original article |
|  |  |  | Sham | | 21 | 30.24 (9.59) | |  | |  |  | |  |  |  |  |
| Speer, et al. (2014) | 3 | 3 | rTMS | | 8 | 39.6 (9) | | LDLPFC | | Yes | 1 Hz (110%) | | Monotherapy | 15 (3w) | HDRS-28 | Previous analysis^5^ |
|  |  |  | rTMS | | 8 | 41.3 (14.5) | | LDLPFC | |  | 20 Hz (110%) | |  | 15 (3w) |  |  |
|  |  |  | Sham | | 8 | 44.9 (9.1) | |  | |  |  | |  |  |  |  |
| Stern, et al. (2007) | 4 | 4 | rTMS | | 10 | 52.8 (9.5) | | LDLPFC | | Yes | 10 Hz (110%) | | Monotherapy | 10 (2w) | HDRS-21 | Previous analysis^5^ |
|  |  |  | rTMS | | 10 | 52.3 (9.4) | | LDLPFC | |  | 1 Hz (110%) | |  | 10 (2w) |  |  |
|  |  |  | rTMS | | 10 | 53.2 (9.4) | | RDLPFC | |  | 1 Hz (110%) | |  | 10 (2w) |  |  |
|  |  |  | Sham | | 15 | 53.3 (9) | |  | |  |  | |  |  |  |  |
| Su, et al. (2005) | 3^1^ | 3 | rTMS | | 22 | 43.4 (11) | | LDLPFC | | Yes | 20 Hz (100%) | | Augmentation | 10 (2w) | HDRS-21 | Previous analysis^5^ |
|  |  |  | Sham | | 11 | 42.6 (11) | |  | |  |  | |  |  |  |  |
| Tavares, et al. (2017) | 2 | 2 | dTMS | | 26 | 43.5 (12) | | LDLPFC | | Yes | 18 Hz (120%) | | Augmentation | 20 (4w) | HDRS-17 | Previous analysis^5^ |
|  |  |  | Sham | | 26 | 41.2 (8.9) | |  | |  |  | |  |  |  |  |
| Taylor, et al. (2018) | 2 | 2 | TMS | | 20 | 46.9 (10.7) | | LDLPFC | | Yes | 10 Hz (120%) | | Mixed | 20 (4w) | HDRS-17 | Previous analysis^5^ |
|  |  |  | Sham | | 20 | 44.1 (11.1) | |  | |  |  | |  |  |  |  |
| Theleritis, et al. (2017) | 4^2^ | 4 | rTMS | | 26 | 39.1 (10.1) | | LDLPFC | | Yes | 20 Hz (100%) | | Mixed | 15 (3w) | HDRS-17 | Previous analysis^5^ |
|  |  |  | rTMS | | 26 | 38.9 (13.9) | | LDLPFC | |  | 20 Hz (100%) | |  | 30 (3w) |  |  |
|  |  |  | Sham | | 44 | 38.8 (9.4) | |  | |  |  | |  |  |  |  |
| Tortella, et al. (2020) | 2 | 2 | tDCS | | 30 | 46.2 (11.8) | | Anode: LDLPFC Cathode: RDLPFC | | Mixed | 2 mA | | Augmentation | 12 (6w) | TMT-A, TMT-B, digit span | Original article |
|  |  |  | Sham | | 29 | 45.7 (10.3) | |  | |  |  | |  |  |  |  |
| Triggs, et al. (2010) | 4^1^ | 4 | rTMS | | 16 | 48.5 (10.8) | | RDLPFC | | Yes | 5 Hz (100%) | | Augmentation | 10 (2w) | HDRS-24 | Previous analysis^5^ |
|  |  |  | rTMS | | 18 | 46.7 (15.3) | | LDLPFC | |  | 5 Hz (100%) | |  | 10 (2w) |  |  |
|  |  |  | Sham | | 14 | 44.3 (17.4) | |  | |  |  | |  |  |  |  |
| Valkonen-Korhonen, et al. (2018) | 2 | 2 | rTMS | | 20 | 37.1 (12.65) | | BLDLPFC | | Yes | 1 Hz R, 10 Hz L (110%, 100%) | | Augmentation | 20 (4w) | HDRS | Original article |
|  |  |  | Sham | | 20 | 30.24 (9.59) | |  | |  |  | |  |  |  |  |
| van Eijndhoven, et al. (2020) | 2 | 2 | rTMS | | 15 | 47.3 (11.5) | | LDLPFC | | Yes | 10 Hz (110%) | | Mixed | 20 (4w) | HDRS-17 | Original article |
|  |  |  | Sham | | 16 | 49.7 (11) | |  | |  |  | |  |  |  |  |
| Wajdik, et al. (2014) | 2 | 2 | rTMS | | 35 | 21-65 | | LDLPFC | | Yes | 10 Hz (110%) | | Mixed | 15 (3-4w) | HDRS-17, TMT-A, TMT-B, digit span | Original article |
|  |  |  | Sham | | 33 |  |  |  | |  |  | |  |  |  |  |
| Yesavage, et al. (2018) | 2 | 2 | rTMS | | 81 | 55.6 (12.2) | | LPFC | | Yes | 10 Hz (120%) | | Augmentation | 20-30 (4-6w) | HDRS-24 | Original article |
|  |  |  | Sham | | 83 | 54.8 (12.6) | |  | |  |  | |  |  |  |  |
| Zavorotnyy, et al. (2020) | 2 | 2 | iTBS | | 27 | 55.6 (12.2) | | LDLPFC | | No | TBS (90%) | | Augmentation | 20 (4w) | HDRS-21 | Original article |
|  |  |  | Sham | | 28 | 54.8 (12.6) | |  | |  |  | |  |  |  |  |
| Zhang, et al. (2020) | 3 | 3 | rTMS | | 24 | 31.71 (12.92) | | Visual Cortex | | No | 10 Hz (90%) | | Monotherapy | 10 (1w) | HDRS-24 | Original article |
|  |  |  | rTMS | | 27 | 31.33 (12.77) | | Individualised Visual Cortex | |  | 10 Hz (90%) | |  | 10 (1w) |  |  |
|  |  |  | Sham | | 23 | 31.61 (10.45) | |  | |  |  | |  |  |  |  |
| Zheng, et al. (2010) | 2 | 2 | rTMS | | 19 | 26.9 (6.2) | | LDLPFC | | Yes | 15 Hz (110%) | | Augmentation | 20 (4w) | HDRS-17 | Previous analysis^5^ |
|  |  |  | Sham | | 15 | 26.7 (4.3) | |  | |  |  | |  |  |  |  |
| **GAD** |  |  |  | |  |  | |  | |  |  | |  |  |  |  |
| de Lima, et al. (2019) | 2 | 2 | tDCS | | 15 | 32.07 (6.5) | | Anode: LDLPFC Cathode: RDLPFC | | No | 2 mA | | Augmentation | 5 (1w) | HRSA | Contacted authors |
|  |  |  | Sham | | 15 | 29 (5.05) | |  | |  |  | |  |  |  |  |
| Diefenbach, et al. (2016) | 2 | 2 | rTMS | | 13 | NR | | RDLPFC | | No | 1 Hz (90%) | | Mixed | 30 (6w) | HRSA | Previous analysis^4^ |
|  |  |  | Sham | | 12 | NR | |  | |  |  | |  |  |  |  |
| Dilkov, et al. (2017) | 2 | 2 | rTMS | | 25 | 34 (7) | | RDLPFC | | No | 20 Hz (110%) | | Mixed | 25 (6w) | HRSA | Previous analysis^4^ |
|  |  |  | Sham | | 25 | 38 (10) | |  | |  |  | |  |  |  |  |
| Huang, et al. (2018) | 2 | 2 | rTMS | | 18 | 44.94 (11.64) | | RPL | | No | 1 Hz (90%) | | Mixed | 10 (2w) | HRSA | Original article |
|  |  |  | Sham | | 18 | 45.22 (10.85) | |  | |  |  | |  |  |  |  |
| Movahed, et al. (2018) | 2 | 3 | tDCS | | 6 | 28.7 (9.6) | | Cathode: LDLPFC Anode: Contralateral deltoid | | No | 2 mA | | Monotherapy | 10 (4w) | HRSA | Original article |
|  |  |  | Sham | | 6 | 28.7 (9.6) | |  | |  |  | |  |  |  |  |
| **OCD** |  |  |  | |  |  | |  | |  |  | |  |  |  |  |
| Alonso, et al. (2001) | 2 | 2 | rTMS | | 10 | 39.2 (13) | | RDLPFC | | Yes | 1 Hz (110%) | | Mixed | 18 (6w) | Y-BOCS | Previous analysis^6^ |
|  |  |  | Sham | | 8 | 30.3 (9.5) | |  | |  |  | |  |  |  |  |
| Arumugham, et al. (2018) | 2 | 2 | rTMS | | 19 | 27.74 (7.88) | | SMA | | Yes | 1 Hz (100%) | | Augmentation | 18 (3w) | Y-BOCS | Previous analysis^6^ |
|  |  |  | Sham | | 17 | 30.7 (10.43) | |  | |  |  | |  |  |  |  |
| Badawy, et al. (2010) | 2 | 2 | rTMS | | 20 | 27.7 (7.83) | | LDLPFC | | Yes | 20 Hz (NR) | | Monotherapy | (3w) | Y-BOCS | Previous analysis^6^ |
|  |  |  | Sham | | 20 | 28.9 (5.7) | |  | |  |  | |  |  |  |  |
| Bation, et al. (2019) | 2 | 2 | tDCS | | 10 | 44.8 (19.9) | | Anode: OFC Cathode: Right Cerebellum | | Yes | 2 mA | | Augmentation | 10 (1w) | Y-BOCS | Original article |
|  |  |  | Sham | | 11 | 41.2 (11.9) | |  | |  |  | |  |  |  |  |
| Carmi, et al. (2019) | 2 | 2 | dTMS | | 47 | 41.1 (11.97) | | MPFC | | Yes | 20 Hz (100%) | | Mixed | 29 (6w) | Y-BOCS | Previous analysis^6^ |
|  |  |  | Sham | | 47 | 36.5 (11.38) | |  | |  |  | |  |  |  |  |
| Dutta, et al. (2021) | 2 | 2 | cTBS | | 18 | 30.5 (12.37) | | OFC | | Yes | 50 Hz (80%) | | Mixed | 10 (1w) | Y-BOCS | Original article |
|  |  |  | Sham | | 15 | 28.33 (7.4) | |  | |  |  | |  |  |  |  |
| Elbeh, et al. (2016) | 3 | 3 | rTMS | | 15 | 28.9 (3.9) | | RDLPFC | | NA | 10 Hz (100%) | | Mixed | 10 (2w) | Y-BOCS | Previous analysis^6^ |
|  |  |  | rTMS | | 15 | 26.8 (5.2) | | RDLPFC | |  | 1 Hz (100%) | |  | 10 (2w) |  |  |
|  |  |  | Sham | | 15 | 25.5 (4) | |  | |  |  | |  |  |  |  |
| Gomes, et al. (2012) | 2 | 2 | rTMS | | 12 | 35.5 (7.5) | | SMA | | Yes | 1 Hz (100%) | | Augmentation | 10 (2w) | Y-BOCS | Previous analysis^6^ |
|  |  |  | Sham | | 10 | 37.5 (16) | |  | |  |  | |  |  |  |  |
| Gowda, et al. (2019) | 2 | 2 | tDCS | | 12 | 30.83 (5.87) | | Anode: Pre-SMA Cathode: RSOA | | Yes | 2 mA | | Mixed | 10 (1w) | Y-BOCS | Original article |
|  |  |  | Sham | | 13 | 25.92 (5.15) | |  | |  |  | |  |  |  |  |
| Haghighi, et al. (2015) | 2 | 2 | rTMS | | 10 | 34.9 (5.91) | | BLDLPFC | | Yes | 20 Hz (100%) | | Mixed | 20 (4w) | Y-BOCS | Previous analysis^6^ |
|  |  |  | Sham | | 11 | 36.6 (3.95) | |  | |  |  | |  |  |  |  |
| Harika-Germaneau, et al. (2019) | 2 | 2 | cTBS | | 14 | 46.3 (10.1) | | SMA | | Yes | TBS (80%) | | Mixed | 30 (6w) | Y-BOCS | Previous analysis^6^ |
|  |  |  | Sham | | 14 | 48.2 (12.9) | |  | |  |  | |  |  |  |  |
| Hawken, et al. (2016) | 2 | 2 | TMS | | 10 | 33 (10) | | SMA | | Yes | 1 Hz (110%) | | Mixed | 25 (6w) | Y-BOCS | Previous analysis^6^ |
|  |  |  | Sham | | 12 | 31 (14) | |  | |  |  | |  |  |  |  |
| Jahangard, et al. (2016) | 2 | 2 | rTMS | | 5 | 32.4 (8.97) | | BLDLPFC | | Yes | 20 Hz (100%) | | Augmentation | 10 (2w) | Y-BOCS | Previous analysis^6^ |
|  |  |  | Sham | | 5 | 33.8 (5.81) | |  | |  |  | |  |  |  |  |
| Kang, et al. (2009) | 2 | 2 | rTMS | | 10 | 28.6 (12.66) | | RDLPFC | | Yes | 1 Hz (110%) | | Mixed | 10 (2w) | Y-BOCS | Previous analysis^6^ |
|  |  |  | Sham | | 10 | 26.2 (10.52) | |  | |  |  | |  |  |  |  |
| Ma, et al. (2014) | 2 | 2 | rTMS | | 25 | 27.12 (8.97) | | BLDLPFC | | Yes | 8-12 Hz (80%) | | Augmentation | 10 (2w) | Y-BOCS | Previous analysis^6^ |
|  |  |  | Sham | | 21 | 29.86 (9.42) | |  | |  |  | |  |  |  |  |
| Mansur, et al. (2011) | 2 | 2 | rTMS | | 13 | 42.1 (11.9) | | RDLPFC | | Yes | 10 Hz (110%) | | Mixed | 30 (6w) | Y-BOCS | Previous analysis^6^ |
|  |  |  | Sham | | 14 | 39.3 (13.9) | |  | |  |  | |  |  |  |  |
| Mantovani, et al. (2010) | 2 | 2 | rTMS | | 9 | 39.7 (8.6) | | SMA | | Yes | 1 Hz (100%) | | Mixed | 20 (4w) | Y-BOCS | Previous analysis^6^ |
|  |  |  | Sham | | 9 | 39.4 (10.2) | |  | |  |  | |  |  |  |  |
| Naro, et al. (2019) | 2 | 2 | iTBS | | 5 | 52 (5) | | LDLPFC | | NA | 20 Hz (80%) | | Mixed | 20 (4w) | Y-BOCS | Previous analysis^6^ |
|  |  |  | Sham | | 5 |  |  |  | |  |  | |  |  |  |  |
| Nauczyciel, et al. (2014) | 2 | 2 | rTMS | | 9 | 40 (NR) | | OFC | | Yes | 1 Hz (120%) | | Mixed | 10 (1w) | Y-BOCS | Previous analysis^6^ |
|  |  |  | Sham | | 10 | 39 (NR) | |  | |  |  | |  |  |  |  |
| Pelissolo, et al. (2016) | 2 | 2 | rTMS | | 20 | 39.1 (10.4) | | SMA | | Yes | 1 Hz (100%) | | Mixed | 20 (4w) | Y-BOCS | Previous analysis^6^ |
|  |  |  | Sham | | 16 | 42.3 (10.6) | |  | |  |  | |  |  |  |  |
| Prasko, et al. (2006) | 2 | 2 | rTMS | | 18 | 28.9 (7.7) | | RDLPFC | | Yes | 1 Hz (110%) | | Augmentation | 10 (2w) | Y-BOCS | Previous analysis^6^ |
|  |  |  | Sham | | 12 | 33.4 (8.7) | |  | |  |  | |  |  |  |  |
| Ruffini, et al. (2009) | 2 | 2 | rTMS | | 16 | 41.5 (NR) | | OFC | | Yes | 1 Hz (80%) | | Augmentation | 15 (3w) | Y-BOCS | Previous analysis^6^ |
|  |  |  | Sham | | 7 | 39.3 (NR) | |  | |  |  | |  |  |  |  |
| Sachdev, et al. (2007) | 2 | 2 | rTMS | | 10 | 29.5 (9.9) | | LDLPFC | | Yes | 10 Hz (110%) | | Mixed | 10 (2w) | Y-BOCS | Previous analysis^6^ |
|  |  |  | Sham | | 8 | 35.8 (8.2) | |  | |  |  | |  |  |  |  |
| Sarkhel, et al. (2010) | 2 | 2 | rTMS | | 21 | 29.38 (6.55) | | RDLPFC | | NA | 10 Hz (110%) | | Mixed | 10 (2w) | Y-BOCS | Previous analysis^6^ |
|  |  |  | Sham | | 21 | 31.95 (7.81) | |  | |  |  | |  |  |  |  |
| Seo, et al. (2016) | 2 | 2 | rTMS | | 14 | 34.6 (9.8) | | RDLPFC | | NA | 1 Hz (100%) | | Augmentation | 15 (2w) | Y-BOCS | Previous analysis^6^ |
|  |  |  | Sham | | 13 | 36.3 (12.5) | |  | |  |  | |  |  |  |  |
| Shayganfard, et al. (2016) | 2 | 2 | rTMS | | 5 | 33.8 (9.55) | | BLDLPFC | | NA | 20 Hz (100%) | | Augmentation | 10 (2w) | Y-BOCS | Previous analysis^6^ |
|  |  |  | Sham | | 5 | 33.2 (7.86) | |  | |  |  | |  |  |  |  |
| Zhang, et al. (2019) | 2 | 2 | rTMS | | 25 | 32.2 (13.25) | | Pre-SMA | | No | 1 Hz (100%) | | Monotherapy | 20 (4w) | Y-BOCS | Previous analysis^6^ |
|  |  |  | Sham | | 24 | 39.4 (17.04) | |  | |  |  | |  |  |  |  |
| **PTSD** |  |  |  | |  |  | |  | |  |  | |  |  |  |  |
| Ahmadizadeh and Rezaei (2018) | 3 | 3 | rTMS | | 19 | 52.1 (7.62) | | BLDLPFC | | No | 20 Hz (100%) | | Mixed | 10 (4w) | PCL-M | Previous analysis^7^ |
|  |  |  | rTMS | | 19 | 51.89 (7.93) | | RDLPFC | |  | 20 Hz (100%) | |  | 10 (4w) |  |  |
|  |  |  | Sham | | 20 | 47.5 (5.61) | |  | |  |  | |  |  |  |  |
| Ahmadizadeh, et al. (2019) | 2 | 2 | tDCS | | 18 | 44.5 (2.34) | | Anode: LDLPFC Cathode: RDLPFC | | No | 2 mA | | Mixed | 10 (2w) | PCL-5 | Original article |
|  |  |  | Sham | | 16 | 43 (2.42) | |  | |  |  | |  |  |  |  |
| Cohen, et al. (2004) | 3 | 3 | rTMS | | 10 | 42.8 (14.8) | | RDLPFC | | No | 10 Hz (80%) | | NI | 10 (2w) | PTSD Checklist | Previous analysis^7^ |
|  |  |  | rTMS | | 8 | 40.8 (9.9) | | RDLPFC | |  | 1 Hz (80%) | |  | 10 (2w) |  |  |
|  |  |  | Sham | | 6 | 41.8 (11.4) | |  | |  |  | |  |  |  |  |
| Leong, et al. (2020) | 3 | 3 | rTMS | | 10 | 43.5 (12.4) | | RDLPFC | | No | 10 Hz (120%) | | Augmentation | 10 (2w) | PCL-C | Original article |
|  |  |  | rTMS | | 11 | 39.2 (13.5) | | RDLPFC | |  | 1 Hz (120%) | |  | 10 (2w) |  |  |
|  |  |  | Sham | | 10 | 49.5 (6.9) | |  | |  |  | |  |  |  |  |
| Nam, et al. (2013) | 2 | 2 | rTMS | | 7 | 36.29 (8.79) | | RDLPFC | | No | 1 Hz (100%) | | Augmentation | 15 (3w) | CAPS | Previous analysis^7^ |
|  |  |  | Sham | | 9 | 32.78 (6.89) | |  | |  |  | |  |  |  |  |
| Philip, et al. (2019)A | 2 | 2 | iTBS | | 25 | 48 (13) | | RDLPFC | | No | TBS (80%) | | Mixed | 10 (2w) | CAPS | Previous analysis^7^ |
|  |  |  | Sham | | 25 | 53 (12) | |  | |  |  | |  |  |  |  |
| Philip, et al. (2019)B | 2 | 2 | sTMS | | 10 | 54.2 (12) | | - | | Mixed | 8-13 Hz (IAF) | | Mixed | 20 (4w) | PCL-5 | Previous analysis^7^ |
|  |  |  | Sham | | 13 |  |  |  | |  |  | |  |  |  |  |
| Watts, et al. (2012) | 2 | 2 | rTMS | | 10 | 54 (12.3) | | RDLPFC | | No | 1 Hz (90%) | | Mixed | 10 (2w) | CAPS | Previous analysis^7^ |
|  |  |  | Sham | | 10 | 57.8 (11.8) | |  | |  |  | |  |  |  |  |
| **Schizophrenia** | |  |  | |  |  | |  | |  |  | |  |  |  |  |
| Bais, et al. (2014) | 3 | 3 | rTMS | | 18 | 37.2 (14.9) | | LTPJ | | Yes | 1 Hz (90%) | | Augmentation | 12 (2w) | PANSS, AHRS | Original article |
|  |  |  | rTMS | | 17 | 33.9 (9.2) | | Primed LTPJ then RTPJ | | Yes | 1 Hz (90%) | | Augmentation | 12 (2w) |  |  |
|  |  |  | Sham | | 16 | 37.3 (11.6) | |  | |  |  | |  |  |  |  |
| Barr, et al. (2011) | 2 | 2 | rTMS | | 12 | 47.21 (12.8) | | BLDLPFC | | NA | 20 Hz (90%) | | Augmentation | 1s | N-back task | Original article |
|  |  |  | Sham | | 12 |  |  |  | |  |  | |  |  |  |  |
| Barr, et al. (2012) | 2 | 2 | rTMS | | 13 | 40.46 (12.21) | | BLDLPFC | | NA | 20 Hz (90%) | | Augmentation | 20 (4w) | PANSS | Original article |
|  |  |  | Sham | | 14 | 47.92 (12.78) | |  | |  |  | |  |  |  |  |
| Barr, et al. (2013) | 2 | 2 | rTMS | | 13 | 41.15 (12.01) | | BLDLPFC | | NA | 20 Hz (90%) | | Augmentation | 20 (4w) | N-back task | Original article |
|  |  |  | Sham | | 15 | 49 (12.42) | |  | |  |  | |  |  |  |  |
| Blumberger et al. (2012)C | 3 | 3 | rTMS | | 17 | 36.6 (8.2) | | LTPJ | | Yes | 1 Hz (115%) | | Augmentation | 20 (4w) | PANSS, PSYRATS | Original article |
|  |  |  | rTMS | | 17 | 43.8 (11.7) | | Primed LTPJ | |  | 6 Hz, 1 Hz (90%, 115%) | |  | 20 (4w) |  |  |
|  |  |  | Sham | | 17 | 40.8 (12.1) | |  | |  |  | |  |  |  |  |
| Bose, et al. (2018) | 2 | 2 | tDCS | | 12 | 31.25 (8.32) | | Anode: LDLPFC, Cathode: LTPJ | | Yes | 2 mA | | Augmentation | 10 (1w) | AHRS, SANS, SAPS | Original article |
|  |  |  | Sham | | 14 | 31.38 (7.56) | |  | |  |  | |  |  |  |  |
| Brunelin, et al. (2006) | 2 | 2 | rTMS | | 14 | 34.9 (8) | | LTPJ | | Yes | 1 Hz (90%) | | Augmentation | 10 (1w) | AHRS | Original article |
|  |  |  | Sham | | 10 | 34 (7) | |  | |  |  | |  |  |  |  |
| Brunelin, et al. (2012) | 2 | 2 | tDCS | | 15 | 40.4 (9.9) | | Anode: LDLPFC Cathode: LTPJ | | Yes | 2 mA | | Augmentation | 10 (1w) | AHRS, PANSS | Original article |
|  |  |  | Sham | | 15 | 35.1 (7) | |  | |  |  | |  |  |  |  |
| Chang, et al. (2018) | 2 | 2 | tDCS | | 30 | 46.4 (10.29) | | Anode: LDLPFC, Cathode: LTPJ | | Yes | 2 mA | | Augmentation | 10 (1w) | AHRS | Original article |
|  |  |  | Sham | | 30 | 42.17 (10.29) | |  | |  |  | |  |  |  |  |
| Chang, et al. (2019) | 2 | 2 | tDCS | | 30 | 46.4 (10.29) | | Anode: LDLPFC, Cathode: LTPJ | | Yes | 2 mA | | Augmentation | 10 (1w) | PANSS, TMT-A, TMT-B, digit span, Conner’s CPT | Original article |
|  |  |  | Sham | | 30 | 42.17 (10.29) | |  | |  |  | |  |  |  |  |
| Chang, et al. (2020) | 2 | 2 | tDCS | | 30 | 44.7 (10.7) | | Anode: BLDLPFC Cathode: Ipsilateral forearm | | Yes | 2 mA | | Augmentation | 10 (1w) | TMT-A, TMT-B, digit span, Conner’s CPT | Original article |
|  |  |  | Sham | | 30 | 45.03 (10.91) | |  | |  |  | |  |  |  |  |
| Chauhan, et al. (2020) | 2 | 2 | iTBS | | 19 | 41.74 (8.85) | | Midline cerebellum | | Yes | TBS (80%) | | Augmentation | 10 (2w) | PANSS | Original article |
|  |  |  | Sham | | 17 | 39.35 (8.22) | |  | |  |  | |  |  |  |  |
| Cordes, et al. (2010) | 2 | 2 | rTMS | | 18 | 34.2 (9.7) | | LDLPFC | | No | 10 Hz (120%) | | Augmentation | 10 (2w) | PANSS | Original article |
|  |  |  | Sham | | 14 | 34.4 (10.5) | |  | |  |  | |  |  |  |  |
| de Jesus, et al. (2011) | 2 | 2 | rTMS | | 8 | 46 (9.84) | | LTPJ | | Yes | 1 Hz (90%) | | Augmentation | 20 (4w) | AHRS | Original article |
|  |  |  | Sham | | 9 | 36.5 (6.36) | |  | |  |  | |  |  |  |  |
| Dlabac-de Lange, et al. (2015) | 2 | 2 | rTMS | | 16 | 41.8 (11.6) | | BLDLPFC | | No | 10 Hz (90%) | | Augmentation | 30 (3w) | PANSS | Original article |
|  |  |  | Sham | | 14 | 32.3 (9.7) | |  | |  |  | |  |  |  |  |
| Dollfus, et al. (2018) | 2 | 2 | rTMS | | 35 | 35.3 (8.3) | | Individualized Left temporal region | | No | 20 Hz (80%) | | Augmentation | 4 (1w) | AHRS | Original article |
|  |  |  | Sham | | 39 | 39.6 (11.4) | |  | |  |  | |  |  |  |  |
| Fitzgerald, et al. (2005) | 2 | 2 | rTMS | | 17 | NR | | LTPJ | | Yes | 1 Hz (90%) | | Augmentation | 20 (4w) | PANSS | Original article |
|  |  |  | Sham | | 16 | NR | |  | |  |  | |  |  |  |  |
| Fitzgerald, et al. (2008)B | 2 | 2 | rTMS | | 10 | 37.2 (10.4) | | BLPFC | | Yes | 10 Hz (110%) | | Augmentation | 15 (3w) | PANSS | Original article |
|  |  |  | Sham | | 10 | 33.2 (9.8) | |  | |  |  | |  |  |  |  |
| Francis, et al. (2019) | 2 | 2 | rTMS | | 9 | 23.4 (3.1) | | BLDLPFC | | NA | 20 Hz (110%) | | Augmentation | 10 (2w) | TOL, BACS digit sequencing | Original article |
|  |  |  | Sham | | 10 | 22.3 (2) | |  | |  |  | |  |  |  |  |
| Fröhlich, et al. (2016) | 2 | 2 | tDCS | | 13 | 43.38 (12.64) | | Anode: LDLPFC, Cathode: LTPJ | | Yes | 2 mA | | Augmentation | 5 (1w) | AHRS, PANSS | Original article |
|  |  |  | Sham | | 13 | 40 (10.74) | |  | |  |  | |  |  |  |  |
| Gan, et al. (2015) | 2 | 2 | TMS | | 32 | 28 (9) | | LDLPFC | | No | 10 Hz (NI) | | Augmentation | 20 (2w) | PANSS | Original article |
|  |  |  | Sham | | 35 |  |  |  | |  |  | |  |  |  |  |
| Garg, et al. (2016) | 2 | 2 | rTMS | | 20 | 32.4 (8.44) | | Cerebellar Vermis | | No | 5/6/7 Hz (NI) | | Augmentation | 10 (2w) | PANSS | Original article |
|  |  |  | Sham | | 20 | 30.75 (7.9) | |  | |  |  | |  |  |  |  |
| Gomes, et al. (2015) | 2 | 2 | tDCS | | 7 | 43.29 (9.72) | | Anode: LDLPFC, Cathode: RDLPFC | | No | 2 mA | | NA | 10 (2w) | PANSS | Original article |
|  |  |  | Sham | | 8 | 34.25 (11.21) | |  | |  |  | |  |  |  |  |
| Gomes, et al. (2018) | 2 | 2 | tDCS | | 12 | 39.17 (9.34) | | Anode: LDLPFC, Cathode: RDLPFC | | No | 2 mA | | NA | 10 (1w) | PANSS, NAB mazes, MCCB | Original article |
|  |  |  | Sham | | 12 | 33.75 (12.08) | |  | |  |  | |  |  |  |  |
| Guan, et al. (2020) | 2 | 2 | rTMS | | 28 | 55.5 (7.3) | | LDLPFC | | No | 20 Hz (110%) | | Augmentation | 40 (8w) | PANSS, RBANS attention | Original article |
|  |  |  | Sham | | 28 | 49.3 (10.2) | |  | |  |  | |  |  |  |  |
| Guleken, et al. (2020) | 2 | 2 | rTMS | | 12 | 36.45 (8.58) | | BLDLPFC | | No | 20 Hz (90%) | | Mixed | 20 (1w) | Stroop interference test, digit span forwards | Original |
|  |  |  | Sham | | 12 | 34.4 (12.1) | |  | |  |  | |  |  |  |  |
| Guse, et al. (2013) | 2 | 2 | rTMS | | 13 | 37 (NR) | | LDLPFC | | No | 10 Hz (110%) | | Augmentation | 15 (3w) | TMT-A, TMT-B, N-back task, selective attention accuracy | Original article |
|  |  |  | Sham | | 12 | 36 (NR) | |  | |  |  | |  |  |  |  |
| Holi, et al. (2004) | 2 | 2 | rTMS | | 11 | 38.5 (10.2) | | LDLPFC | | No | 10 Hz (100%) | | Augmentation | 10 (2w) | PANSS | Original article |
|  |  |  | Sham | | 11 | 34.8 (9.8) | |  | |  |  | |  |  |  |  |
| Huang, et al. (2016) | 2 | 2 | rTMS | | 21 | 40.58 (3.01) | | LDLPFC | | No | 10 Hz (110%) | | Augmentation | 21 (3w) | PANSS | Original article |
|  |  |  | Sham | | 20 | 39.39 (3.03) | |  | |  |  | |  |  |  |  |
| Jeon, et al. (2018) | 2 | 2 | tDCS | | 26 | 40 (9.41) | | Anode: LDLPFC, Cathode: RDLPFC | | No | 2 mA | | Augmentation | 10 (2w) | PANSS, MCCB | Original article |
|  |  |  | Sham | | 30 | 39.86 (12.42) | |  | |  |  | |  |  |  |  |
| Kantrowitz, et al. (2019) | 2 | 2 | tDCS | | 47 | 38.2 (9.9) | | Anode: LDLPFC Cathode: LTPJ | | Yes | 2 mA | | Mixed | 10 (2w) | AHRS | Original article |
|  |  |  | Sham | | 42 | 40.1 (8.6) | |  | |  |  | |  |  |  |  |
| Kimura, et al. (2016) | 2 | 2 | rTMS | | 16 | 44.6 (10.5) | | LTPJ | | Yes | 20 Hz (80%) | | Augmentation | 4 (1w) | AHRS | Original article |
|  |  |  | Sham | | 14 | 40.7 (9) | |  | |  |  | |  |  |  |  |
| Klein, et al. (1999)B | 2 | 2 | rTMS | | 18 | 30.2 (10) | | RDLPFC | | No | 1 Hz (110%) | | Augmentation | 10 (2w) | PANSS | Original article |
|  |  |  | Sham | | 17 | 29.5 (9.3) | |  | |  |  | |  |  |  |  |
| Koops, et al. (2016) | 2 | 2 | cTBS | | 37 | 38 (15) | | LTPJ | | Yes | TBS (NR) | | Augmentation | 10 (1w) | AHRS, PANSS | Original article |
|  |  |  | Sham | | 34 | 42 (13) | |  | |  |  | |  |  |  |  |
| Koops, et al. (2018) | 2 | 2 | tDCS | | 30 | 44 (11) | | Anode: LDLPFC, Cathode: LTPJ | | Yes | 2 mA | | Mixed | 10 (1w) | AHRS | Original article |
|  |  |  | Sham | | 34 | 44 (12) | |  | |  |  | |  |  |  |  |
| Kumar, et al. (2020) | 2 | 2 | rTMS | | 50 | 32.4 (9.2) | | LDLPFC | | No | 20 Hz (100%) | | Mixed | 20 (4w) | PANSS | Original article |
|  |  |  | Sham | | 50 | 30.8 (9.34) | |  | |  |  | |  |  |  |  |
| Lee, et al. (2005) | 3 | 3 | rTMS | | 13 | 41.3 (10.3) | | LTPJ | | Yes | 1 Hz (100%) | | Augmentation | 10 (2w) | AHRS, PANSS | Original article |
|  |  |  | rTMS | | 12 | 39.7 (6.9) | | RTPJ | |  | 1 Hz (100%) | |  | 10 (2w) |  |  |
|  |  |  | Sham | | 14 | 39.9 (8.9) | |  | |  |  | |  |  |  |  |
| Lindenmayer, et al. (2019) | 2 | 2 | tDCS | | 15 | 40.2 (10.69) | | Anode: LDLPFC, Cathode: LTPJ | | Yes | 2 mA | | Augmentation | 40 (4w) | PANSS, MCCB | Original article |
|  |  |  | Sham | | 13 |  |  |  | |  |  | |  |  |  |  |
| Meiron, et al. (2021) | 2 | 2 | tDCS | | 11 | 38.48 (14.1) | | Anode: Brodmann area Cathode: Cz^3^ | | No | 2 mA | | Augmentation | 10 (1w) | PANSS | Original article |
|  |  |  | Sham | | 8 |  |  |  | |  |  | |  |  |  |  |
| Mogg, et al. (2007) | 2 | 2 | rTMS | | 8 | 50.8 (14.5) | | LDLPFC | | NA | 10 Hz (110%) | | Augmentation | 10 (2w) | COWAT | Original article |
|  |  |  | Sham | | 9 | 33.6 (9.8) | |  | |  |  | |  |  |  |  |
| Paillère-Martinot, et al. (2017) | 2 | 2 | rTMS | | 15 | 32.07 (6.79) | | Individualized Left temporal region | | Yes | 1 Hz (100%) | | Augmentation | 10 (2w) | AHRS, SAPS, SANS | Original article |
|  |  |  | Sham | | 12 | 31.25 (7.78) | |  | |  |  | |  |  |  |  |
| Palm, et al. (2016) | 2 | 2 | tDCS | | 10 | 38.4 (12.9) | | Anode: LDLPFC, Cathode: ROFC | | No | 2 mA | | Augmentation | 10 (2w) | PANSS | Original article |
|  |  |  | Sham | | 10 | 34.1 (10.7) | |  | |  |  | |  |  |  |  |
|  |  |  |  | |  |  | |  | |  |  | |  |  |  |  |
| Prikryl, et al. (2007) | 2 | 2 | rTMS | | 11 | 31.36 (8.43) | | LDLPFC | | No | 10 Hz (110%) | | Augmentation | 15 (3w) | PANSS | Original article |
|  |  |  | Sham | | 11 | 36.46 (10.74) | |  | |  |  | |  |  |  |  |
| Prikryl, et al. (2012) | 2 | 2 | rTMS | | 19 | 30.47 (9.19) | | LDLPFC | | No | 10 Hz (110%) | | Augmentation | 15 (3w) | PANSS | Original article |
|  |  |  | Sham | | 11 | 34.55 (10.57) | |  | |  |  | |  |  |  |  |
| Prikryl, et al. (2013) | 2 | 2 | rTMS | | 23 | 31.6 (8.04) | | LPFC | | No | 10 Hz (110%) | | Augmentation | 15 (3w) | PANSS | Original article |
|  |  |  | Sham | | 17 | 33.94 (9.98) | |  | |  |  | |  |  |  |  |
| Prikryl, et al. (2014) | 2 | 2 | rTMS | | 18 | 30.4 (6.56) | | LDLPFC | | No | 10 Hz (110%) | | Augmentation | 21 (3w) | PANSS | Original article |
|  |  |  | Sham | | 17 | 34.58 (10.66) | |  | |  |  | |  |  |  |  |
| Quan, et al. (2015) | 2 | 2 | rTMS | | 78 | 46.87 (7.87) | | LDLPFC | | No | 10 Hz (80%) | | Augmentation | 10 (2w) | PANSS | Original article |
|  |  |  | Sham | | 39 | 46.87 (9.07) | |  | |  |  | |  |  |  |  |
| Rabany, et al. (2014) | 2 | 2 | dTMS | | 20 | 33.1 (11.31) | | LDLPFC | | No | 20 Hz (120%) | | Augmentation | 20 (3w) | PANSS | Original article |
|  |  |  | Sham | | 10 | 35.9 (11) | |  | |  |  | |  |  |  |  |
| Ren, et al. (2010) | 2 | 2 | rTMS | | 12 | 32 (7) | | BLDLPFC | | Yes | 1 Hz (80%) | | Augmentation | 10 (2w) | PANSS | Original article |
|  |  |  | Sham | | 13 |  |  |  | |  |  | |  |  |  |  |
| Saba, et al. (2006) | 2 | 2 | rTMS | | 8 | 30.7 (7.95) | | LTPJ | | No | 1 Hz (80%) | | Augmentation | 10 (2w) | PANSS | Original article |
|  |  |  | Sham | | 8 | 30.6 (8) | |  | |  |  | |  |  |  |  |
| Singh, et al. (2020) | 2 | 2 | rTMS | | 15 | 33.3 (9.8) | | LDLPFC | | No | 20 Hz (100%) | | Augmentation | 20 (4w) | PANSS | Original article |
|  |  |  | Sham | | 15 | 29.8 (5.7) | |  | |  |  | |  |  |  |  |
| Slotema, et al. (2011) | 3 | 3 | rTMS | | 22 | 38 (9.6) | | LTPJ | | Yes | 1 Hz (90%) | | Augmentation | 15 (3w) | AHRS, PANSS | Original article |
|  |  |  | rTMS | | 20 | 36 (10) | | Individualized Left temporal region | |  | 1 Hz (90%) | |  |  |  |  |
|  |  |  | Sham | | 20 | 41 (10.30) | |  | |  |  | |  |  |  |  |
| Smith, et al. (2015) | 2 | 2 | tDCS | | 17 | 46.76 (11.06) | | Anode: LDLPFC Cathode: ROFC | | NA | 2 mA | | Augmentation | 5 (1w) | MCCB | Original article |
|  |  |  | Sham | | 16 | 44.88 (9.19) | |  | |  |  | |  |  |  |  |
| Valiengo, et al. (2020) | 2 | 2 | tDCS | | 50 | 34.6 (8.4) | | Anode: LDLPFC Cathode: LTPJ | | Mixed | 2 mA | | Augmentation | 10 (1w) | AHRS, PANSS | Original article |
|  |  |  | Sham | | 50 | 35.9 (10.1) | |  | |  |  | |  |  |  |  |
| Wobrock, et al. (2015) | 2 | 2 | rTMS | | 76 | 36.2 (10.5) | | LDLPFC | | No | 10 Hz (110%) | | Augmentation | 15 (3w) | PANSS | Original article |
|  |  |  | Sham | | 81 | 34.9 (9.1) | |  | |  |  | |  |  |  |  |
| Xiu, et al. (2020) | 3 | 3 | rTMS | | 40 | 50.7 (9) | | LDLPFC | | No | 10 Hz (110%) | | Augmentation | 40 (8w) | PANSS | Original article |
|  |  |  | rTMS | | 40 | 52 (10.1) | | LDLPFC | |  | 20 Hz (110%) | |  | 40 (8w) |  |  |
|  |  |  | Sham | | 40 | 54.7 (6.4) | |  | |  |  | |  |  |  |  |
| Zhao, et al. (2014) | 4 | 4 | rTMS | | 24 | 48 (12.2) | | LDLPFC | | No | 10 Hz (80-110%) | | Mixed | 20 (4w) | PANSS | Original article |
|  |  |  | rTMS | | 23 | 49.1 (10.6) | | LDLPFC | |  | 20 Hz (80-110%) | |  | 20 (4w) |  |  |
|  |  |  | rTMS | | 24 | 47.7 (11.8) | | LDLPFC | |  | TBS (80-110%) | |  | 20 (4w) |  |  |
|  |  |  | Sham | | 22 | 46.7 (13.1) | |  | |  |  | |  |  |  |  |
| Zheng, et al. (2012) | 4 | 4 | rTMS | | 19 | 56.5 (7.4) | | LDLPFC | | No | 10 Hz (80%) | | Augmentation | 5 (1w) | PANSS, visual spatial working memory | Original article |
|  |  |  | rTMS | | 19 | 56.8 (5.4) | | LDLPFC | |  | 20 Hz (80%) | |  | 5 (1w) |  |  |
|  |  |  | iTBS | | 18 | 56.4 (9.3) | | LDLPFC | |  | 50 Hz (80%) | |  | 5 (1w) |  |  |
|  |  |  | Sham | | 17 | 55.6 (5.8) | |  | |  |  | |  |  |  |  |
| Zhuo, et al. (2019) | 2 | 2 | rTMS | | 35 | 28.97 (7.4) | | LDLPFC | | No | 20 Hz (90%) | | Augmentation | 20 (4w) | NAB mazes, WMS: working memory | Original article |
|  |  |  | Sham | | 35 | 30.63 (8.25) | |  | |  |  | |  |  |  |  |
| **SUD** |  |  |  | |  |  | |  | |  |  | |  |  |  |  |
| Alizadehgoradel, et al. (2020) | 2 | 2 | tDCS | | 19 | 34.31 (9.62) | | Anode: LDLPFC Cathode: RDLPFC | | No | 2 mA | | Monotherapy | 10 (5w) | DDQ | Original article |
|  |  |  | Sham | | 20 | 35.35 (8.71) | |  | |  |  | |  |  |  |  |
| Batista, et al. (2015) | 2 | 2 | tDCS | | 19 | 30.4 (9.8) | | Cathode: LDLPDC Anode: RDLPFC | | No | 2 mA | | Monotherapy | 9 (1w) | Craving score | Previous analysis^8^ |
|  |  |  | Sham | | 17 | 30.3 (8.4) | |  | |  |  | |  |  |  |  |
| Gaudreault, et al. (2021) | 2 | 2 | tDCS | | 8 | 40.4 (10.2) | | Anode: RDLPFC Cathode: LDLPFC | | No | 2 mA | | Augmentation | 15 (5w) | OCCS | Original article |
|  |  |  | Sham | | 6 | 46.7 (13.9) | |  | |  |  | |  |  |  |  |
| Holla, et al. (2020) | 2 | 2 | tDCS | | 11 | 38.6 (7.1) | | Anode: LDLPFC Cathode: RDLPFC | | No | 2 mA | | Monotherapy | 5 (1w) | ACQ-SF-R | Original article |
|  |  |  | Sham | | 10 | 39.4 (7.9) | |  | |  |  | |  |  |  |  |
| Klauss, et al. (2018)A | 2 | 2 | tDCS | | 23 | 46.3 (12) | | Anode: RDLPFC Cathode: LDLPFC | | No | 2 mA | | Monotherapy | 10 (3w) | OCDS | Original article |
|  |  |  | Sham | | 22 | 43.5 (10.2) | |  | |  |  | |  |  |  |  |
| Klauss, et al. (2018)B | 2 | 2 | tDCS | | 19 | 35.1 (8.2) | | Anode: RDLPFC Cathode: LDLPFC | | No | 2 mA | | Mixed | 19 (2w) | OCCS | Previous analysis^8^ |
|  |  |  | Sham | | 14 | 35 (9.6) | |  | |  |  | |  |  |  |  |
| Liang, et al. (2018) | 2 | 2 | rTMS | | 24 | 31.8 (1.9) | | LDLPFC | | No | 10 Hz (100%) | | Monotherapy | 12 (2w) | Craving core | Previous analysis^8^ |
|  |  |  | Sham | | 22 | 34.4 (2.3) | |  | |  |  | |  |  |  |  |
| Martinez, et al. (2018) | 3^1^ | 2 | TMS | | 12 | 43 (6) | | Individualized PFC | | No | 10 Hz / 1 Hz (110%) | | Monotherapy | 13 (3w) | Craving score | Previous analysis^8^ |
|  |  |  | Sham | | 6 | 44 (6) | |  | |  |  | |  |  |  |  |
| Martinotti, et al. (2019) | 2 | 2 | tDCS | | 18 | 40.3 (10.1) | | Anode: LDLPFC Cathode: RDLPFC | | No | 1.5 mA | | Mixed | 5 (1w) | Craving score | Original article |
|  |  |  | Sham | | 16 | 37.6 (10.9) | |  | |  |  | |  |  |  |  |
| Su, et al. (2017) | 2 | 2 | rTMS | | 15 | 31.85 (5.25) | | LDLPFC | | No | 10 Hz (80%) | | Monotherapy | 1 | Craving score | Previous analysis^8^ |
|  |  |  | Sham | | 15 | 32.84 (4.79) | |  | |  |  | |  |  |  |  |
| **Tics/Tourette’s Syndrome** |  | | |  | | |  | |  | | |  |  |  |  |  |
| Landeros-Weisenberger, et al. (2015) |  |  | rTMS | | 9 | 29.1 (7.4) | | SMA | | Mixed | 1Hz (110%) | | Mixed | 15 (3w) | YGTSS | Original article |
|  |  |  | Sham | | 11 | 37.5 (14.2) | |  | |  |  | |  |  |  |  |
| Wu, et al. (2014) |  |  | cTBS | |  | 13.5 (3.9) | | SMA | | No | 30Hz (90%) | | Mixed | 2 (2d) | YGTSS | Original article |
|  |  |  | Sham | |  | 15.5 (4) | |  | |  |  | |  |  |  |  |

*Note.* TR = Lifetime treatment resistance, %MT – Percentage of motor threshold. Stimulation techniques; cTBS = Continuous theta-burst stimulation; dTMS = Deep transcranial magnetic stimulation; iTBS = Intermittent theta-burst stimulation; piTBS = Prolonged intermittent theta-burst stimulation; rTMS = repetitive transcranial magnetic stimulation; sTMS = Synchronised transcranial magnetic stimulation; TDCS = Transcranial direct current stimulation. Stimulation sites; BL = Bilateral dorsolateral prefrontal cortex: DLPFC = Dorsolateral prefrontal cortex; DMPFC = Dorsomedial prefrontal cortex; MPFC = Medial prefrontal cortex; OFC = Orbitofrontal cortex; PL = Parietal lobe; SMA = Supplementary motor area; SOA = Supraorbital area; TPJ = Temporal parietal junction; VLPFC = Ventrolateral prefrontal cortex. Outcome scales/tasks; ACQ-SF-R = Alcohol Craving Questionnaire – Short Form – Revised; AHRS = Auditory Hallucination Rating Scale; ASRS: Adult ADHD Scale Symptom Checklist; BACS = Brief Assessment of Cognition; CAARS = Conner’s Adult ADHD Rating Scale; CAPS = Clinician Administered PTSD Scale for DSM-5; CDRS = Cognitive Drug Computerised Assessment System; Conner’s CPT = Conner’s Continuous Performance Task; COWAT = Controlled Oral Word Association Task; DDQ = Desires for Drug Questionnaire; HDRS = Hamilton Depression Rating Scale; HRSA = Hamilton Anxiety Rating Scale; MADRS = Montgomery Asberg Depression Rating Scale; MCCB = MATRICS Consensus Cognitive Battery; NAB = Neuropsychological Assessment Battery; OCCS = Obsessive-Compulsive Cocaine Scale; OCDS = Obsessive-Compulsive Drinking Scale; PANSS = Positive and Negative Symptom Scale; PCL-C = Posttraumatic Checklist – Civilian; PCL-M = Posttraumatic Checklist – Military; RBANS = Repeatable Battery for the Assessment of Neuropsychological Status; SANS = Scale for the Assessment of Negative Symptoms; SAPS = Scale for the Assessment of Positive Symptoms; TMT-A = Trail making task – part A; TMT-B = Trail making task – part B; TOL = Tower of London Task; TVA = Theory of visual attention; WMS = Weschler Memory Scale; Y-BOCS = Yale-Brown Obsessive Compulsive Scale; YGTSS = Yale Global Tic Severity Scale. ^1^ treatment groups were combined. ^2^ sham groups were combined. ^3^ electrode placement based on 10-20 EEG system. ^4^ Cirillo P, Gold AK, Nardi AE, et al. Transcranial magnetic stimulation in anxiety and trauma-related disorders: A systematic review and meta-analysis. *Brain Behav.* 2019;9(6):e01284. ^5^ Mutz J, Vipulananthan V, Carter B, Hurlemann R, Fu CHY, Young AH. Comparative efficacy and acceptability of non-surgical brain stimulation for the acute treatment of major depressive episodes in adults: systematic review and network meta-analysis. *BMJ.* 2019;364:l1079. ^6^ Perera MPN, Mallawaarachchi S, Miljevic A, Bailey NW, Herring SE, Fitzgerald PB. Repetitive Transcranial Magnetic Stimulation for Obsessive-Compulsive Disorder: A Meta-analysis of Randomized, Sham-Controlled Trials. Biol Psychiatry Cogn Neurosci Neuroimaging. 2021;6(10):947-960. ^7^ Kan RLD, Zhang BBB, Zhang JJQ, Kranz GS. Non-invasive brain stimulation for posttraumatic stress disorder: a systematic review and meta-analysis. Transl Psychiatry. 2020;10(1):168. ^8^ Ma T, Sun Y, Ku Y. Effects of Non-invasive Brain Stimulation on Stimulant Craving in Users of Cocaine, Amphetamine, or Methamphetamine: A Systematic Review and Meta-Analysis. Front Neurosci. 2019;13:10. ^9^ Beam W, Brockardt JJ, Reeves ST, George MS. An efficient and accurate new method for locating the F3 position for prefrontal TMS applications. Brain Stimul 2009;2(1):50–54. [PubMed: 20539835]

**Supplementary Table 2. Risk of bias assessments**

|  | **Risk of bias arising from the randomisation process** | **Risk of bias due deviations from the intended interventions** | **Missing outcome data** | **Risk of bias in measurement of outcome** | **Risk of bias in selection of the reported result** | **Overall risk of bias** |
| --- | --- | --- | --- | --- | --- | --- |
| Ahmadizadeh 2018 | Low | Low | Low | Low | Some concerns | Some concerns |
| Ahmadizadeh 2019 | Low | High | High | Low | Some concerns | High |
| Alizadegoradel 2020 | Low | Low | Low | Low | Some concerns | Some concerns |
| Alonso 2001 | Some concerns | Low | Low | Low | Some concerns | Some concerns |
| Alyagon 2020 | Low | Low | High | Low | Some concerns | High |
| Anderson 2007 | Low | High | Some concerns | Low | Some concerns | High |
| Arumugham 2018 | Low | Low | Low | Low | Low | Low |
| Avery 1999 | Some concerns | Low | Low | Low | Some concerns | Some concerns |
| Avery 2006 | Low | High | High | Low | Some concerns | High |
| Badawy 2010 | Some concerns | Low | Low | Low | Some concerns | Some concerns |
| Baeken 2013 | Some concerns | Low | Low | Low | Some concerns | Low |
| Bais 2014 | Low | Low | Low | Low | Low | Low |
| Bakim 2012 | Low | Low | Some concerns | Low | Some concerns | Some concerns |
| Barr 2011 | Low | Low | Low | Low | Some concerns | Some concerns |
| Barr 2012 | Some concerns | Low | Some concerns | Low | Some concerns | Some concerns |
| Barr 2013 | Some concerns | Low | Some concerns | Low | Some concerns | Some concerns |
| Bation 2019 | Low | Low | Low | Low | Some concerns | Some concerns |
| Batista 2015 | Low | Some concerns | Some concerns | High | Some concerns | High |
| Berman 2000 | Some concerns | Low | Low | Low | Some concerns | Some concerns |
| Beynel 2014 | Low | Low | Low | Low | Some concerns | Some concerns |
| Blumberger 2012A | Some concerns | Low | Low | Low | Some concerns | Some concerns |
| Blumberger 2012B | Some concerns | Low | Low | Low | Low | Some concerns |
| Blumberger 2012C | Low | Some concerns | Low | Low | Some concerns | Some concerns |
| Blumberger 2016 | Low | Low | Low | Low | Some concerns | Some concerns |
| Bortolomasi 2007 | Low | Low | Low | Low | Some concerns | Some concerns |
| Bose 2018 | Low | Low | Low | Low | Some concerns | Some concerns |
| Boutros 2002 | Low | Low | Low | Low | Some concerns | Some concerns |
| Brunelin 2006 | Low | Low | Low | Low | Some concerns | Some concerns |
| Brunelin 2012 | Low | Low | Low | Low | Some concerns | Some concerns |
| Bulteau 2019 | Low | Low | Low | High | High | High |
| Carmi 2019 | Low | Low | Low | Low | Low | Low |
| Carpenter 2017 | Low | High | High | Low | Some concerns | High |
| Chang 2018 | Some concerns | Low | Some concerns | Low | Some concerns | Some concerns |
| Chang 2019 | Some concerns | Low | Low | Low | Low | Some concerns |
| Chang 2020 | Low | High | High | Low | Some concerns | High |
| Chauhan 2020 | Low | Low | Low | Low | Low | Low |
| Chen 2013 | Low | Low | Low | Low | Some concerns | Some concerns |
| Chistyahov 2015 | Low | Some concerns | Low | Low | Some concerns | Some concerns |
| Chou 2020 | Low | Low | Low | Low | Some concerns | Some concerns |
| Cohen 2004 | Low | High | Some concerns | Low | Some concerns | High |
| Concerto 2015 | Low | Low | Low | Low | Some concerns | Some concerns |
| Cordes 2010 | Low | Low | Low | Low | Some concerns | Some concerns |
| de Jesus 2011 | Some concerns | Low | Low | Low | Some concerns | Some concerns |
| de Lima 2019 | Low | Low | Low | Low | Low | Low |
| Diefenbach 2016 | Some concerns | Low | High | Low | Low | High |
| Dilkov 2017 | Low | Low | Low | Low | Low | Low |
| Dlabac 2015 | Some concerns | Low | Low | Low | Some concerns | Some concerns |
| Dollfus 2018 | Low | Low | Some concerns | Low | Some concerns | Some concerns |
| Dunlop 2020 | Low | Low | Some concerns | Low | Some concerns | Some concerns |
| Duprat 2016 | Some concerns | Low | Low | Low | Low | Some concerns |
| Dutta 2021 | Low | Low | Low | Low | High | High |
| Elbeh 2016 | Low | Low | Low | Low | Some concerns | Some concerns |
| Eschweiler 2000 | Some concerns | Some concerns | High | Low | Some concerns | High |
| Eshel 2020 | Low | Some concerns | Some concerns | Low | Low | Some concerns |
| Fitzgerald 2003 | Low | Low | Low | Low | Some concerns | Some concerns |
| Fitzgerald 2005 | Low | High | High | Low | Some concerns | High |
| Fitzgerald 2006 | Low | Low | Some concerns | Low | Some concerns | Some concerns |
| Fitzgerald 2008A | Low | Some concerns | Low | Low | Some concerns | Some concerns |
| Fitzgerald 2008B | Low | High | High | Low | Some concerns | Some concerns |
| Fitzgerald 2012 | Low | High | High | Low | Some concerns | High |
| Fitzgerald 2016 | Low | Low | Some concerns | Low | Low | Some concerns |
| Francis 2019 | Low | Some concerns | Low | Low | Some concerns | Some concerns |
| Fregni 2006 | Some concerns | Low | Low | Low | Some concerns | Some concerns |
| Frohlich 2016 | Low | Low | Low | Low | Some concerns | Some concerns |
| Gan 2015 | Low | Low | Low | Some concerns | Some concerns | Some concerns |
| Garcia-toro 2001 | Low | High | High | Low | Some concerns | High |
| Garcia-toro 2006 | Low | Low | Low | Low | Some concerns | Some concerns |
| Garg 2016 | Low | High | High | Low | Some concerns | High |
| Gaudreault 2021 | Low | High | High | Low | Some concerns | High |
| George 2010 | Low | High | High | Low | Some concerns | High |
| George 1997 | Low | Low | Low | Low | Some concerns | Some concerns |
| George 2000 | Low | Low | Low | Low | Some concerns | Some concerns |
| Gogler 2017 | Low | Low | Low | Low | Some concerns | Some concerns |
| Gomes 2012 | Low | Low | Low | Low | Some concerns | Some concerns |
| Gomes 2015 | High | High | High | Low | Some concerns | High |
| Gomes 2018 | Some concerns | Some concerns | Low | Low | Some concerns | Some concerns |
| Gowda 2019 | Low | Low | Low | Low | Some concerns | Some concerns |
| Guan 2020 | Low | Low | High | Low | Some concerns | Some concerns |
| Guleken 2020 | Some concerns | Low | Low | Low | Low | Some concerns |
| Guse 2013 | Low | High | High | Low | Some concerns | High |
| Haghigi 2015 | Low | Low | Low | Low | Some concerns | Some concerns |
| Harika-Germaneau 2019 | Low | Low | Low | Low | Some concerns | Some concerns |
| Hawken 2016 | Low | High | High | Low | Some concerns | Some concerns |
| He 2011 | Low | High | Some concerns | Low | Some concerns | High |
| Hernandez-Ribas 2013 | Some concerns | Low | Low | Low | Some concerns | Some concerns |
| Holi 2004 | Low | Some concerns | Low | Low | Some concerns | Some concerns |
| Holla 2020 | Low | Low | Low | Low | Some concerns | Some concerns |
| Holtzheimer 2004 | Low | Low | Some concerns | Low | Some concerns | Some concerns |
| Hoppner 2003 | Some concerns | Low | Low | Low | Some concerns | Some concerns |
| Huang 2016 | Low | Low | Some concerns | Low | Some concerns | Some concerns |
| Huang 2018 | Some concerns | Low | Low | Low | Some concerns | Some concerns |
| Jahangard 2016 | Low | Low | Low | Low | Some concerns | Some concerns |
| Januel 2006 | Some concerns | Low | Low | Low | Some concerns | Some concerns |
| Jeon 2018 | Low | Some concerns | Low | Low | Some concerns | Some concerns |
| Jin 2014 | Some concerns | High | High | Low | Some concerns | High |
| Kang 2009 | Low | Low | Low | Low | Some concerns | Some concerns |
| Kang 2016 | Low | Some concerns | Low | Low | High | High |
| Kantrowitz 2019 | Low | Low | Low | Low | Low | Low |
| Kaster 2018 | Low | Low | Low | Low | Low | Low |
| Kauffmann 2004 | Some concerns | Low | Low | Low | Some concerns | Some concerns |
| Kavanaugh 2018 | Low | High | High | Low | Some concerns | High |
| Kimbrell 1999 | Some concerns | High | High | Low | Some concerns | High |
| Kimura 2016 | Some concerns | Low | Low | Low | Some concerns | Some concerns |
| Klauss 2018A | Low | Low | Low | Low | Low | Low |
| Klauss 2018B | Low | Low | Low | Low | Low | Low |
| Klein 1999A | Some concerns | Low | Low | Low | Some concerns | Some concerns |
| Klein 1999B | Low | Low | Low | Low | Some concerns | Some concerns |
| Koerselman 2004 | Low | Low | Low | Low | Some concerns | Some concerns |
| Koops 2016 | Low | Low | Low | Low | Some concerns | Some concerns |
| Koops 2018 | Low | Low | Low | Low | Low | Low |
| Kreuzer 2015 | Low | Low | High | Low | Low | High |
| Kumar 2020 | Low | High | Low | Low | Some concerns | High |
| Lee 2005 | Some concerns | Low | Low | Low | Some concerns | Some concerns |
| Lee 2018 | Low | High | High | Low | Some concerns | High |
| Lee 2019 | Some concerns | Low | High | Low | Some concerns | High |
| Leong 2020 | Low | Low | Low | Low | Low | Low |
| Leuchter 2015 | Low | High | Some concerns | Low | Low | High |
| Levkovitz 2015 | Low | Low | Some concerns | Low | Some concerns | Some concerns |
| Li 2014 | Low | Low | Low | Low | Some concerns | Some concerns |
| Li 2020 | Low | Low | Low | Low | Low | Low |
| Liang 2018 | Low | Low | Low | Low | Low | Low |
| Lindenmayer 2019 | Low | Low | Low | Low | Some concerns | Some concerns |
| Lingeswaran 2011 | Low | Low | Some concerns | Low | Some concerns | Some concerns |
| Loo 2001 | Some concerns | Low | Low | Low | Some concerns | Some concerns |
| Loo 2003 | Low | Low | Low | Low | Some concerns | Some concerns |
| Loo 2007 | Low | Some concerns | Low | Low | Some concerns | Some concerns |
| Loo 2010 | Low | High | High | Low | Low | High |
| Loo 2012 | Low | Low | Low | Low | Low | Low |
| Loo 2018 | Low | Low | Low | Low | Low | Low |
| Ma 2014 | Low | Low | Some concerns | Low | Some concerns | Some concerns |
| Manes 2001 | Low | Low | Low | Low | Some concerns | Some concerns |
| Mansur 2011 | Low | Low | High | Low | Some concerns | High |
| Mantovani 2010 | Low | Low | Low | Low | Some concerns | Some concerns |
| Martinez 2018 | Low | Low | Low | Some concerns | Some concerns | Some concerns |
| Martinotti 2019 | Low | Low | Low | Low | Some concerns | Some concerns |
| Matsuda 2020 | Low | Some concerns | Low | Low | Some concerns | Some concerns |
| McDonald 2006 | Some concerns | Low | High | Low | Some concerns | High |
| McGirr 2021 | Low | Low | Low | Low | Low | Low |
| Meiron 2021 | Low | Low | Low | Low | Some concerns | Some concerns |
| Mogg 2007 | Low | Low | Low | Low | Some concerns | Some concerns |
| Mogg 2008 | Low | Some concerns | Low | Low | Some concerns | Some concerns |
| Moser 2002 | Low | Low | Low | Low | Some concerns | Some concerns |
| Mosimann 2004 | Low | Low | Low | Low | Some concerns | Some concerns |
| Movahed 2018 | Some concerns | Low | Low | Low | Some concerns | Some concerns |
| Myczkowski 2018 | Low | Low | Low | Low | Low | Low |
| Nahas 2003 | Low | Some concerns | Some concerns | Low | Some concerns | Some concerns |
| Nam 2013 | Low | Low | Low | Low | Some concerns | Some concerns |
| Naro 2019 | Some concerns | Low | Low | Low | Some concerns | Some concerns |
| Nauczyciel 2014 | Some concerns | High | Low | Low | Some concerns | High |
| O'reardon 2007 | Low | High | Low | Low | Some concerns | High |
| Padberg 2002 | Low | Low | Low | Low | Some concerns | Some concerns |
| Padberg 1999 | Low | Low | Low | Low | Some concerns | Some concerns |
| Paillere-Martinot 2010 | Low | Low | Low | Low | Some concerns | Some concerns |
| Paillere-Martinot 2017 | Low | Low | Low | Low | Some concerns | Some concerns |
| Palm 2016 | Low | Low | Low | Low | Some concerns | Some concerns |
| Pallanti 2010 | Low | Low | Low | Low | High | High |
| Paz 2018 | Low | Low | High | Low | Some concerns | High |
| Pelissolo 2016 | Low | Low | Low | Low | Some concerns | Some concerns |
| Philip 2019A | Low | Some concerns | Low | Low | Some concerns | Some concerns |
| Philip 2019B | Low | Some concerns | Low | Low | Some concerns | Some concerns |
| Prasko 2006 | Low | Low | Low | Low | Some concerns | Some concerns |
| Prasser 2015 | Low | Some concerns | Low | Low | Some concerns | Some concerns |
| Prikryl 2007 | Low | Low | Low | Low | Some concerns | Some concerns |
| Prikryl 2012 | Low | Low | Low | Low | Some concerns | Some concerns |
| Prikryl 2013 | Low | Low | Low | Low | Some concerns | Some concerns |
| Prikryl 2014 | Low | High | High | Low | Some concerns | High |
| Quan 2015 | Some concerns | Low | Low | Low | Some concerns | Some concerns |
| Rabany 2014 | Some concerns | Some concerns | Some concerns | Low | Some concerns | Some concerns |
| Ren 2010 | Low | Low | Low | Low | Some concerns | Some concerns |
| Rossini 2005 | Low | Some concerns | Low | Low | Some concerns | Some concerns |
| Ruffini 2009 | Low | Low | Low | Low | Some concerns | Some concerns |
| Saba 2006 | Low | Low | Some concerns | Low | Some concerns | Some concerns |
| Sachdev 2007 | Low | Low | Low | Low | Some concerns | Some concerns |
| Salehinejad 2015 | Some concerns | Low | Low | Low | Some concerns | Some concerns |
| Salehinejad 2017 | Low | Low | Low | Low | Some concerns | Some concerns |
| Sampaio Junior 2018 | Low | Low | Low | Low | Low | Low |
| Sarkhel 2010 | High | Some concerns | Low | Some concerns | Some concerns | High |
| Schutter 2009 | Low | Some concerns | Low | Low | Low | Some concerns |
| Seo 2016 | Some concerns | Low | Low | Low | Some concerns | Some concerns |
| Sharafi 2019 | Low | Low | Low | Low | Some concerns | Some concerns |
| Shayganfard 2016 | Low | Low | Low | Low | Some concerns | Some concerns |
| Singh 2020 | Low | High | Low | Low | Low | High |
| Slotema 2011 | Low | Low | Some concerns | Low | Some concerns | Some concerns |
| Smith 2015 | Low | Low | Low | Low | Some conerns | Some concerns |
| Song 2020 | Some concerns | High | High | Low | Some concerns | High |
| Speer 2014 | Some concerns | Some concerns | Low | Low | Some concerns | Some concerns |
| Stern 2007 | Low | Some concerns | Low | Low | Some concerns | Some concerns |
| Su 2005 | Some concerns | Low | Some concerns | Low | Some concerns | Some concerns |
| Su 2017 | Low | Low | Low | Low | Some concerns | Some concerns |
| Tavares 2017 | Low | High | High | Low | Some concerns | High |
| Taylor 2018 | Some concerns | Some concerns | High | Low | Some concerns | High |
| Theleritis 2017 | Low | High | Some concerns | Low | Some concerns | High |
| Tortella 2020 | Low | Low | Low | Low | Low | Low |
| Triggs 2010 | Low | Low | Low | Low | Some concerns | Some concerns |
| Valiengo 2020 | Low | Low | Low | Low | Low | Low |
| Valkonen-Korhonen 2018 | Low | Low | Some concerns | Low | Some concerns | Some concerns |
| Van Eijndhoven 2020 | Some concerns | Low | Low | Low | Some concerns | Some concerns |
| Wajdik 2014 | Some concerns | Low | High | Low | Some concerns | High |
| Watts 2012 | Low | Low | Low | Low | Some concerns | Some concerns |
| Wobrock 2015 | Low | Some concerns | Some concerns | Low | Some concerns | Some concerns |
| Xiu 2020 | Low | Low | High | Low | Low | High |
| Yeasavage 2018 | Some concerns | High | Some concerns | Low | Low | High |
| Zavorotnyy 2020 | Low | Some concerns | Some concerns | Low | Some concerns | Some concerns |
| Zhang 2019 | Low | Low | Low | Low | Some concerns | Some concerns |
| Zhang 2020 | Low | Some concerns | Some concerns | Low | Some concerns | Some concerns |
| Zhao 2014 | Low | Low | Low | Some concerns | Some concerns | Some concerns |
| Zheng 2010 | Low | Low | Low | Low | Some concerns | Some concerns |
| Zheng 2012 | Low | Low | Low | Low | Some concerns | Some concerns |
| Zhuo 2019 | Low | Low | Some concerns | Low | Low | Some concerns |

Supplementary table 3 shows the results of risk of bias assessments conducted on each study using the Revised Cochrane risk of bias tool for randomized trial.

**Supplementary Figure 1.** Forest plot showing effect sizes for TMS treating attention deficit hyperactivity disorder (ADHD).

| **Q** | ***p*** | **I^2^** |
| --- | --- | --- |
| 2.11 | .146 | 52 |

**Supplementary Figure 2.** Forest plot showing effect sizes for TMS treating depressive episodes (overall).

| **Q** | ***p*** | **I^2^** |
| --- | --- | --- |
| 2.11 | .146 | 52 |

**Supplementary Figure 3.** Forest plot showing effect sizes for TMS treating depressive episodes (unipolar).

| **Q** | ***p*** | **I^2^** |
| --- | --- | --- |
| 154.91 | < .001 | 74 |

**Supplementary Figure 4.** Forest plot showing effect sizes for TMS treating depressive episodes (bipolar).

| **Q** | ***p*** | **I^2^** |
| --- | --- | --- |
| 6.09 | .107 | 51 |

**Supplementary Figure 5.** Forest plot showing effect sizes for TMS treating generalized anxiety disorder (GAD).

| **Q** | ***p*** | **I^2^** |
| --- | --- | --- |
| 5.37 | .068 | 63 |

**Supplementary Figure 6.** Forest plot showing effect sizes for TMS treating obsessive compulsive disorder (OCD).

| **Q** | ***p*** | **I^2^** |
| --- | --- | --- |
| 67.05 | < .001 | 67 |

**Supplementary Figure 7.** Forest plot showing effect sizes for TMS treating posttraumatic stress disorder (PTSD).

| **Q** | ***p*** | **I^2^** |
| --- | --- | --- |
| 42.44 | < .001 | 79 |

**Supplementary Figure 8.** Forest plot showing effect sizes for TMS treating positive symptoms of schizophrenia (SCZ).

| **Q** | ***p*** | **I^2^** |
| --- | --- | --- |
| 153.20 | < .001 | 77 |

**Supplementary Figure 9.** Forest plot showing effect sizes for TMS treating negative symptoms of schizophrenia (SCZ).

| **Q** | ***p*** | **I^2^** |
| --- | --- | --- |
| 133.98 | < .001 | 78 |

**Supplementary Figure 10.** Forest plot showing effect sizes for TMS treating total symptoms of schizophrenia (SCZ).

| **Q** | ***p*** | **I^2^** |
| --- | --- | --- |
| 58.67 | < .001 | 52 |

**Supplementary Figure 11.** Forest plot showing effect sizes for TMS treating total symptoms of schizophrenia (SCZ).

| **Q** | ***p*** | **I^2^** |
| --- | --- | --- |
| 12.62 | < .001 | 0 |

**Supplementary Figure 12.** Forest plot showing effect sizes for TMS treating substance use disorders (SUD).

| **Q** | ***p*** | **I^2^** |
| --- | --- | --- |
| 49.44 | < .001 | 92 |

**Supplementary Figure 13.** Funnel plot for TMS treating depressive episodes (overall).

**Supplementary Figure 14.** Funnel plot for TMS treating depressive episodes (unipolar).

**Supplementary Figure 15.** Funnel plot for TMS treating obsessive compulsive disorder (OCD).

**Supplementary Figure 16.** Funnel plot for TMS treating posttraumatic stress disorder (PTSD).

**Supplementary Figure 17.** Funnel plot for TMS treating positive symptoms of schizophrenia (SCZ).

**Supplementary Figure 18.** Funnel plot for TMS treating negative symptoms of schizophrenia (SCZ).

**Supplementary Figure 19.** Funnel plot for TMS treating total symptoms of schizophrenia (SCZ).

**Supplementary Figure 20.** Funnel plot for TMS treating auditory hallucination symptoms of schizophrenia (SCZ).

**Supplementary Figure 21.** Forest plot showing effect sizes for tDCS treating depression episodes (overall).

| **Q** | ***p*** | **I^2^** |
| --- | --- | --- |
| 67.89 | < .001 | 88 |

**Supplementary Figure 22.** Forest plot showing effect sizes for tDCS treating generalized anxiety disorder (GAD).

| **Q** | ***p*** | **I^2^** |
| --- | --- | --- |
| 0.55 | .457 | 0 |

**Supplementary Figure 23.** Forest plot showing effect sizes for tDCS treating obsessive compulsive disorder (OCD).

| **Q** | ***p*** | **I^2^** |
| --- | --- | --- |
| 0.003 | .953 | 0 |

**Supplementary Figure 24.** Forest plot showing effect sizes for tDCS treating positive symptoms of schizophrenia (SCZ).

| **Q** | ***p*** | **I^2^** |
| --- | --- | --- |
| 3.59 | .826 | 0 |

**Supplementary Figure 25.** Forest plot showing effect sizes for tDCS treating negative symptoms of schizophrenia (SCZ).

| **Q** | ***p*** | **I^2^** |
| --- | --- | --- |
| 14.98 | .020 | 60 |

**Supplementary Figure 26.** Forest plot showing effect sizes for tDCS treating negative symptoms of schizophrenia (SCZ).

| **Q** | ***p*** | **I^2^** |
| --- | --- | --- |
| 26.14 | .001 | 69 |

**Supplementary Figure 27.** Forest plot showing effect sizes for tDCS treating auditory hallucination symptoms of schizophrenia (SCZ).

| **Q** | ***p*** | **I^2^** |
| --- | --- | --- |
| 16.50 | .011 | 64 |

**Supplementary Figure 28.** Forest plot showing effect sizes for tDCS treating substance use disorders (SUD).

| **Q** | ***p*** | **I^2^** |
| --- | --- | --- |
| 2.95 | .815 | 0 |

**Supplementary Figure 29.** Forest plot showing effect of TMS treatment as compared to sham treatment on performance in attention tasks in patients with depression.

| **Q** | ***p*** | **I^2^** |
| --- | --- | --- |
| 0.97 | .617 | 0 |

**Supplementary Figure 30.** Forest plot showing effect of TMS treatment as compared to sham treatment on performance in attention tasks in patients with schizophrenia.

| **Q** | ***p*** | **I^2^** |
| --- | --- | --- |
| 3.26 | .196 | 39 |

**Supplementary Figure 31.** Forest plot showing effect of TMS treatment as compared to sham treatment on performance in executive functioning tasks in patients with depression.

| **Q** | ***p*** | **I^2^** |
| --- | --- | --- |
| 7.46 | .383 | 6 |

**Supplementary Figure 32.** Forest plot showing effect of TMS treatment as compared to sham treatment on performance in executive functioning tasks in patients with schizophrenia.

| **Q** | ***p*** | **I^2^** |
| --- | --- | --- |
| 6.82 | .233 | 41 |

**Supplementary Figure 33.** Forest plot showing effect of TMS treatment as compared to sham treatment on performance in processing speed tasks in patients with depression.

| **Q** | ***p*** | **I^2^** |
| --- | --- | --- |
| 4.71 | .582 | 0 |

**Supplementary Figure 34.** Forest plot showing effect of TMS treatment as compared to sham treatment on performance in processing speed tasks in patients with schizophrenia.

| **Q** | ***p*** | **I^2^** |
| --- | --- | --- |
| 1.84 | .765 | 0 |

**Supplementary Figure 35.** Forest plot showing effect of TMS treatment as compared to sham treatment on performance in working memory tasks in patients with depression.

| **Q** | ***p*** | **I^2^** |
| --- | --- | --- |
| 3.88 | .694 | 0 |

**Supplementary Figure 36.** Forest plot showing effect of TMS treatment as compared to sham treatment on performance in working memory tasks in patients with schizophrenia.

| **Q** | ***p*** | **I^2^** |
| --- | --- | --- |
| 9.18 | .156 | 2 |

**Supplementary Figure 37.** Forest plot showing effect of TMS treatment as compared to sham treatment on performance in working memory tasks in patients with substance use disorders (SUD).

| **Q** | ***p*** | **I^2^** |
| --- | --- | --- |
| 5.95 | .015 | 83 |

**Supplementary Figure 38.** Forest plot showing effect of tDCS treatment as compared to sham treatment on performance in attention tasks in patients with schizophrenia.

| **Q** | ***p*** | **I^2^** |
| --- | --- | --- |
| 6.15 | .292 | 19 |

**Supplementary Figure 39.** Forest plot showing effect of tDCS treatment as compared to sham treatment on performance in executive functioning tasks in patients with depression.

| **Q** | ***p*** | **I^2^** |
| --- | --- | --- |
| 0.12 | .942 | 0 |

**Supplementary Figure 40.** Forest plot showing effect of tDCS treatment as compared to sham treatment on performance in executive functioning tasks in patients with schizophrenia.

| **Q** | ***p*** | **I^2^** |
| --- | --- | --- |
| 3.76 | .710 | 0 |

**Supplementary Figure 41.** Forest plot showing effect of tDCS treatment as compared to sham treatment on performance in processing speed tasks in patients with depression.

| **Q** | ***p*** | **I^2^** |
| --- | --- | --- |
| 1.34 | .512 | 0 |

**Supplementary Figure 42.** Forest plot showing effect of tDCS treatment as compared to sham treatment on performance in processing speed tasks in patients with schizophrenia.

| **Q** | ***p*** | **I^2^** |
| --- | --- | --- |
| 14.23 | .027 | 58 |

**Supplementary Figure 43.** Forest plot showing effect of tDCS treatment as compared to sham treatment on performance in working memory tasks in patients with depression.

| **Q** | ***p*** | **I^2^** |
| --- | --- | --- |
| 4.54 | .338 | 12 |

**Supplementary Figure 44.** Forest plot showing effect of tDCS treatment as compared to sham treatment on performance in working memory tasks in patients with schizophrenia.

| **Q** | ***p*** | **I^2^** |
| --- | --- | --- |
| 9.86 | .131 | 39 |
